# A Flexible Synthetic Strategy for the Preparation of Heteroleptic Metallacycles of Porphyrins

**DOI:** 10.1021/acs.inorgchem.1c01511

**Published:** 2021-07-15

**Authors:** Alessio Vidal, Federica Battistin, Gabriele Balducci, Elisabetta Iengo, Enzo Alessio

**Affiliations:** Department of Chemical and Pharmaceutical Sciences, University of Trieste, Via L. Giorgieri 1, 34127 Trieste, Italy

## Abstract

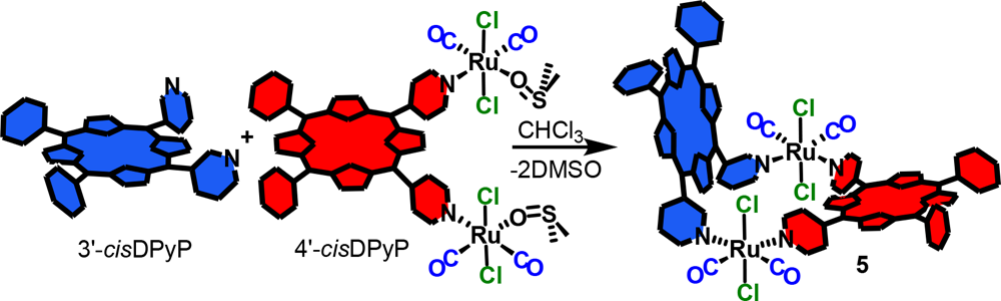

We present a stepwise synthetic strategy for the preparation of the unprecedented heteroleptic 2+2 neutral metallacycle [{*t,c,c*-RuCl_2_(CO)_2_}_2_(4′*cis*DPyP)(3′*cis*DPyP)] (**5**), in which two different 5,10-*meso*-dipyridylporphyrins, 4′*cis*DPyP [i.e., 5,10-bis(4′-pyridyl)-15,20-diphenylporphyrin] and 3′*cis*DPyP [i.e., 5,10-bis(3′-pyridyl)-15,20-diphenylporphyrin], are joined through equal 90°-angular Ru(II) connectors. The synthesis of **5** was accomplished through the preparation of a reactive ditopic intermediate in which one of the two pyridylporphyrins is linked to two neutral ruthenium fragments, each having one residual readily available coordination site (a dmso-O). Thus, compound **5** was obtained under mild conditions through two complementary routes: either by treatment of [{*t*,*c*,*c*-RuCl_2_(CO)_2_(dmso-O)}_2_(4′*cis*DPyP)] (**3**) with 1 equiv of 3′*cis*DPyP or, alternatively, by treatment of [{*t,c,c*-RuCl_2_(CO)_2_(dmso-O)}_2_(3′*cis*DPyP)] (**4**) with 1 equiv of 4′*cis*DPyP. Heteroleptic metallacycle **5** was isolated in pure form in acceptable yield and fully characterized. Spectroscopic data and a molecular model show that **5** has an L-shaped geometry, with the two porphyrins almost orthogonal to one another. The modular approach that we established is highly flexible and opens the way to several possible exciting developments.

## Abstract 2

The unprecedented heteroleptic 2+2 neutral metallacycle [{*t,c,c*-RuCl_2_(CO)_2_}_2_(4′*cis*DPyP)(3′*cis*DPyP)] (**5**), which features two different 5,10-*meso*-dipyridylporphyrins and has an L-shaped geometry, was isolated in pure form through a flexible stepwise synthetic approach.

## Introduction

Nature uses sophisticated arrays of tetrapyrrolic macrocycles (e.g., chlorophyll, cytochromes, etc.) to perform precise energy and electron transfer processes. In addition to the specific nature of the macrocycles, their number and relative orientations are of paramount importance for determining the properties of such assemblies. The development of simple procedures for preparing synthetic arrays of tetrapyrrole macrocycles with full stereocontrol is one of the challenges of supramolecular chemistry.

The metal-mediated self-assembly approach, which exploits the formation of coordination bonds between peripheral basic site(s) on the porphyrins and suitable metal centers, has afforded a variety of discrete two-dimensional (2D) and three-dimensional (3D) arrays of porphyrins of the type M_*x*_(porp)_*y*_, including several 2+2 and 4+4 metallacycles (M can be a naked ion or bear ancillary ligands).^1−15^ In this context, *meso*-pyridylporphyrins, PyPs,^16^ which feature one to four pyridyl moieties in *meso* positions, have been largely exploited.^1,17−24^

**Figure 1:**
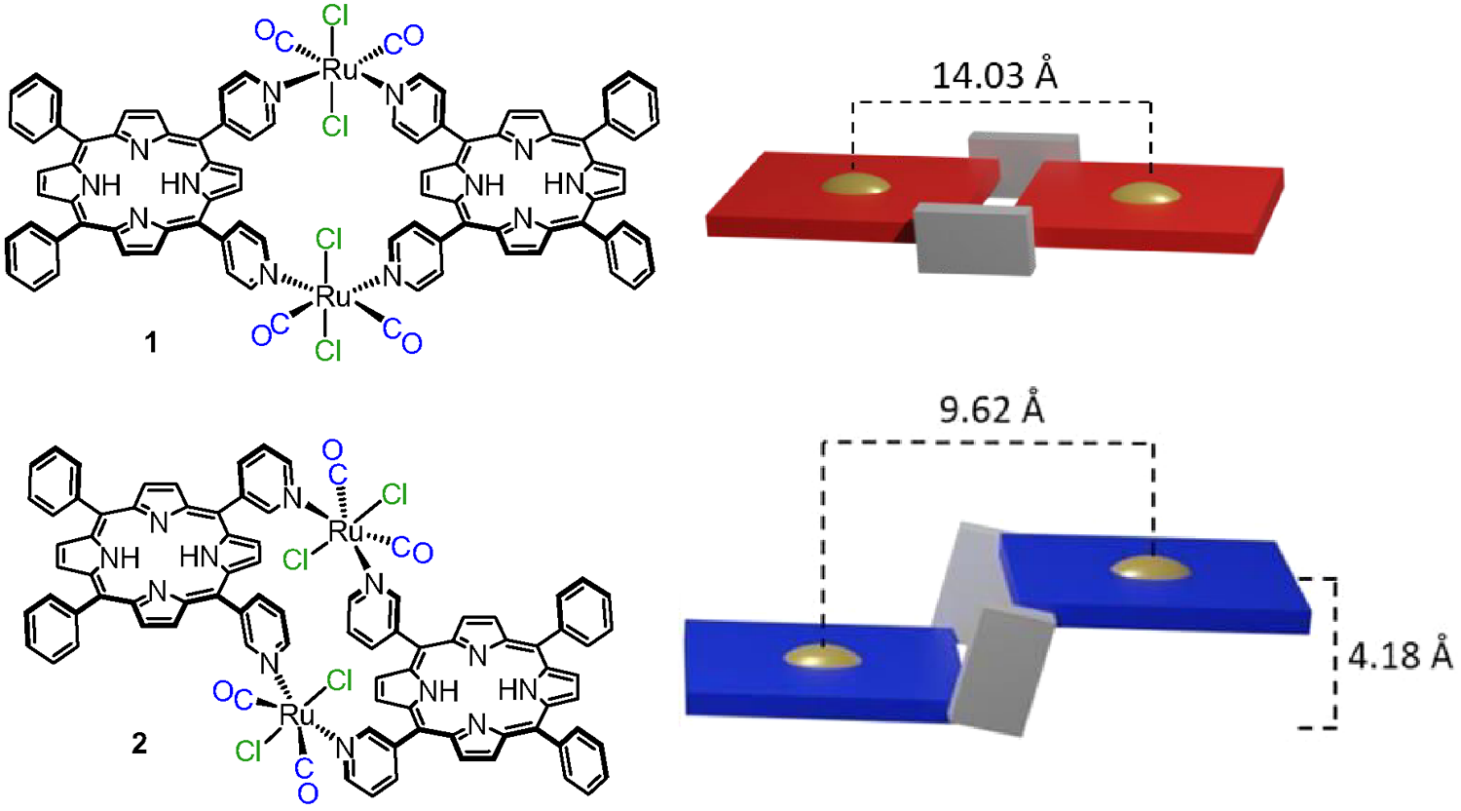
Homoleptic 2+2 metallacycles **1** (top) and **2** (bottom) with their schematic representations as molecular panels (right). Distances from the X-ray structures.^26^ Color code: red for 4′*cis*DPyP, blue for 3′*cis*DPyP, gray for {*t,c,c*-RuCl_2_(CO)_2_}, and gold for the centroid of the porphyrin.

In the past, we prepared in good yields and fully characterized the neutral 2+2 metallacycles [*t,c,c*-RuCl_2_(CO)_2_(4′*cis*DPyP)]_2_ (**1**) and [*t,c,c*-RuCl_2_(CO)_2_(3′*cis*DPyP)]_2_ (**2**), in which two identical 5,10-*meso*-dipyridylporphyrins, either 5,10-bis(4′-pyridyl)-15,20-diphenylporphyrin (i.e., 4′*cis*DPyP) or 5,10-bis(3′-pyridyl)-15,20-diphenylporphyrin (i.e., 3′*cis*DPyP), are connected through two 90°-angular {*t,c,c*-RuCl_2_(CO)_2_} fragments (Figure 1);^25,26^ the neutral Ru linkers, in addition to affording stable and inert bonds, are highly symmetric and thus generate no stereoisomers. X-ray structural characterization showed that whereas **1** is perfectly flat in the solid state, **2** has a staggered geometry with the two chromophores rigidly held in a slipped-cofacial arrangement by the Ru(II) fragments (Figure 1). For each porphyrin in **2**, (i) the two pyridyl rings are in a *syn* conformation and (ii) the plane of the heterocycle is almost orthogonal to the equatorial coordination plane (N, N, C, and C) of each Ru linker.

The corresponding zincated metallacycles **1Zn** and **2Zn**, in which each embedded metal center is capable of forming an additional axial bond, were exploited by us as two-point molecular panels (Figure 1) for the preparation of molecular sandwiches, boxes, and prisms.^25,27,28^

**Figure 2:**
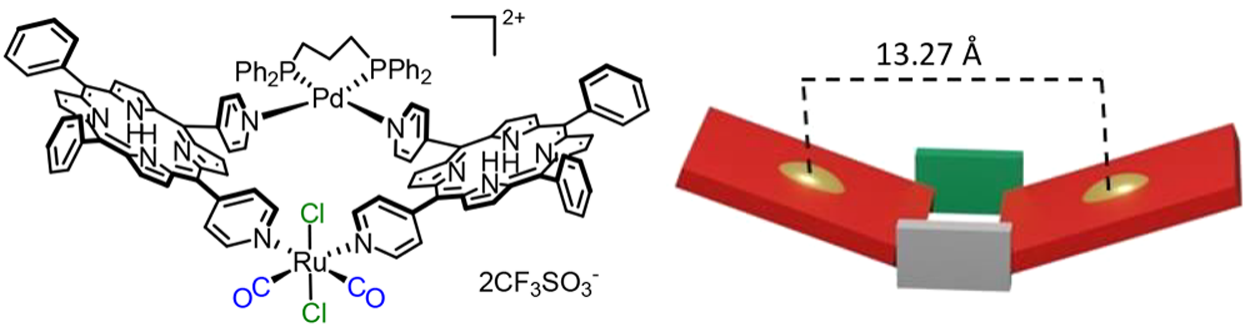
Heterobimetallic 2+2 metallacycle of porphyrins [Pd(dppp){*t*,*c*,*c*-RuCl_2_(CO)_2_(4′*cis*DPyP)_2_}](CF_3_SO_3_)_2_ (dppp = 1,3-bis(diphenylphosphanyl)propane) with its schematic representation shown as a molecular panel (right). Distances from the X-ray structure.^29^ Color code: red for 4′*cis*DPyP, gray for {*t,c,c*-RuCl_2_(CO)_2_}, green for {Pd(dppp)}^2+^, and gold for the centroid of the porphyrin.

A vast majority of the metal-mediated arrays of porphyrins described in the literature, including metallacycles **1** and **2**, are homoleptic systems, as they contain a single type of porphyrin. With few exceptions, they are also homometallic, because most of them contain a single type of metal connector. In the past, we described the stepwise preparation of the heterobimetallic 2+2 metallacycle of porphyrins [Pd(dppp){*t*,*c*,*c*-RuCl_2_(CO)_2_(4′*cis*DPyP)_2_}](CF_3_SO_3_)_2_ [dppp = 1,3-bis(diphenylphosphanyl)propane] that features an octahedral neutral Ru(II) complex at one corner and a square planar cationic Pd(II) linker at the other (Figure 2).^29^ In the solid state, the metallacycle, obtained by treatment of the reactive 90° metal-containing ligand *t*,*c*,*c*-[RuCl_2_(CO)_2_(4′-*cis*DPyP)_2_] with the *cis*-protected complex [Pd(dppp)(CF_3_SO_3_)_2_], was found to have a folded geometry in which the two porphyrins form a dihedral angle of 41.7(1)°.

More recently, we described new stereoisomers of homoleptic molecular square **1**, in which one or both {*t,c,c*-RuCl_2_(CO)_2_} corners are replaced by the stereoisomeric {*c,c,c*-RuCl_2_(CO)_2_} fragment, which can have a *C* or *A* configuration, namely, [{*t,c,c*-RuCl_2_(CO)_2_}(4′*cis*DPyP)_2_{*c,c,c*-RuCl_2_(CO)_2_}] and [*c,c,c*-RuCl_2_(CO)_2_(4′*cis*DPyP)]_2_.^30^

The examples of metal-mediated assemblies containing different porphyrins are even rarer. In 1998, Drain and co-workers described the high-yield one-pot formation of a nonameric neutral square-shaped grid obtained by addition of 12 equiv of [PdCl_2_(NCPh)_2_], a precursor of the linear {*trans*-PdCl_2_} linker, to a 1:4:4 mixture of three different *meso*-4′-pyridylporphyrins:^16^ one X-shaped 4′-TPyP unit (taking the central position; 4′-TPyP = 5,10,15,20-tetrapyridylporphyrin), four T-shaped 4′-TrPyP units [making the sides of the array; 4′-TrPyP = 5,10,15-tris(4′-pyridyl)-20-phenylporphyrin], and four angular 4′*cis*DPyP units (making the corners).^31^ The adduct, which was not isolated, was believed to be the thermodynamic product, and the reversibility of the pyridyl–Pd(II) bond was apparently a critical feature for the high selectivity of the preparation. On the contrary, with a similar one-pot approach, Pd(II)- or Pt(II)-mediated one-dimensional (1D) and 2D “tapes” of pyridylporphyrins were obtained with much lower selectivities, as mixtures of adducts with different nuclearities.^31^ The same group later described a mixed porphyrin/porphyrazine assembly obtained by postsynthetic modification.^32,33^ More recently, Schmittel and co-workers, exploiting the HETTAP concept for controlling the coordination equilibrium at the metal ion,^34^ prepared Cu-mediated assemblies containing two different cofacial metalloporphyrins.^35,36^ It should be noted that in this case the two porphyrins have very different peripheral binding sites, one being a pyridyl ring and the other a sterically shielded phenanthroline, and the strategy exploits steric and electronic effects originating from the latter.

Given these premises, we aimed to develop a new flexible synthetic strategy that might be used for the construction of ruthenium-mediated heteroleptic systems of *meso*-pyridylporphyrins containing PyPs that differ in the number of peripheral pyridyl rings (from two to four) and/or in the position of the pyridyl N atom (3′ or 4′). This would open the way to new extended arrays as well as to unprecedented geometries. The formation of Ru-mediated metallacycles of PyPs typically occurs under kinetic control; therefore, a one-pot synthetic approach does not seem to be suitable for our aim. In fact, because all PyPs share the pyridyl ring as a common binding motif, no stereoelectronic discrimination upon coordination to ruthenium can be expected to occur. Thus, we decided to develop a new stepwise modular approach that requires the initial preparation of a reactive polytopic “acceptor” intermediate, i.e., a pyridylporphyrin bound to at least two ruthenium fragments, each having one residual readily available coordination site, that is then reacted with the second porphyrin to yield the final heteroleptic assembly. Dimetallic acceptors, not porphyrin-based and typically with a 2+ charge, have been extensively used by several groups for self-assembly purposes. For example, Stang and co-workers exploited di-Pt(II) acceptors of different shapes for the preparation of many metallacycles and metallacages.^37^ Dimetallic molecular clips were used as pillars for the preparation of metallacages featuring two equal face-to-face flat organic linkers, including porphyrins.^1,37−44^

**Scheme 1:**
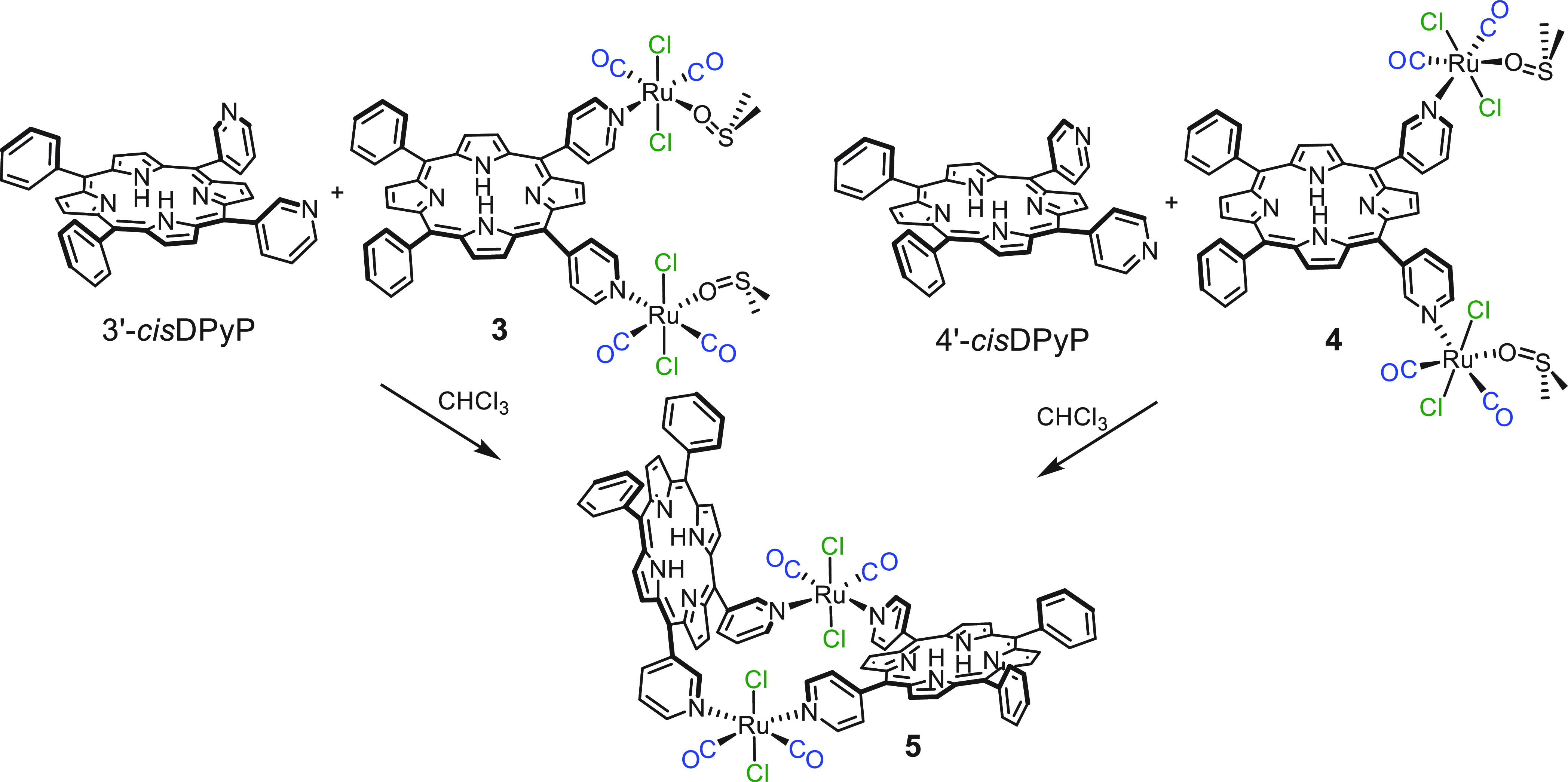
Two Alternative Routes for the Stepwise Preparation of Neutral Metallacycle [{*t,c,c*-RuCl_2_(CO)_2_}_2_(4′*cis*DPyP)(3′*cis*DPyP)] (**5**), either through the Ditopic Reactive Intermediate [{*t*,*c*,*c*-RuCl_2_(CO)_2_(dmso-O)}_2_(4′*cis*DPyP)] (**3**, left) or through [{*t,c,c*-RuCl_2_(CO)_2_(dmso-O)}_2_(3′*cis*DPyP)] (**4**, right).

As a proof of concept, this strategy was here first used for the stepwise preparation of the heteroleptic 2+2 neutral metallacycle [{*t,c,c*-RuCl_2_(CO)_2_}_2_(4′*cis*DPyP)(3′*cis*DPyP)] (**5**), in which two different *cis*-dipyridylporphyrins, 4′*cis*DPyP and 3′*cis*DPyP, are joined through equal 90°-angular Ru(II) connectors. The synthesis of **5** required the preparation of the reactive ditopic intermediate [{*t*,*c*,*c*-RuCl_2_(CO)_2_(dmso-O)}_2_(4′*cis*DPyP)] (**3**), which already contains one of the two porphyrins, that was then treated with 1 equiv of 3′*cis*DPyP (Scheme 1). Alternatively, compound **5** was obtained by treatment of the reactive intermediate [{*t,c,c*-RuCl_2_(CO)_2_(dmso-O)}_2_(3′*cis*DPyP)] (**4**) with 1 equiv of 4′*cis*DPyP (Scheme 1). Heteroleptic metallacycle **5** was isolated in pure form in reasonable yield and fully characterized.^45^ Spectroscopic data and the molecular model are consistent with **5** having an unprecedented L-shaped geometry, with the two porphyrins almost orthogonal to one another.

## Results and Discussion

### Model Systems

**Scheme 2:**
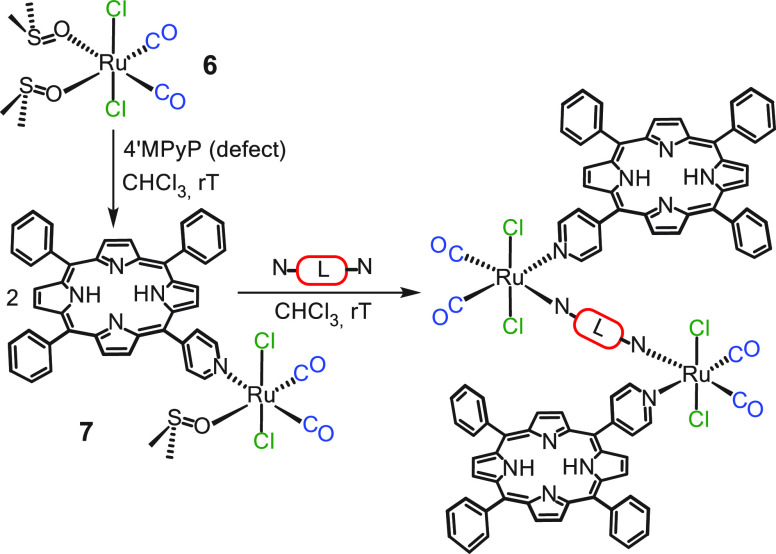
Stepwise Preparation of Metal-Mediated Assemblies [{*t,c,c*-RuCl_2_(CO)_2_(4′MPyP)}_2_(μ-L)] (with N–L–N = 4,4′-bpy or 4′*trans*DPyP) through Reactive Intermediate *t,c,c*-[RuCl_2_(CO)_2_(dmso-O)(4′MPyP)] (**7**)

Because the purification of *cis*DPyPs is a laborious process, we first ascertained that the two dmso-O ligands in the Ru(II)-dmso carbonyl complex *t,c,c*-[RuCl_2_(CO)_2_(dmso-O)_2_] (**6**), precursor of the metal corners in **1** and **2**,^46^ can be indeed replaced in a stepwise manner by pyridyl ligands using 5-(4′-pyridyl)-10,15,20-triphenylporphyrin (4′MPyP) as a model. As described in the Supporting Information, we first isolated *t,c,c*-[RuCl_2_(CO)_2_(dmso-O)(4′MPyP)] (**7**) in good yield^47^ and then demonstrated that treatment of **7** at room temperature with 0.5 equiv of linear linkers 4,4′-bpy and 4′*trans*DPyP afforded the corresponding adducts [{*t,c,c*-RuCl_2_(CO)_2_(4′MPyP)}_2_(μ-4,4′-bpy)] and [{*t,c,c*-RuCl_2_(CO)_2_(4′MPyP)}_2_(μ-4′*trans*DPyP)], respectively (Scheme 2).

### Synthesis and Characterization of Reactive Ditopic Intermediate [{*t*,*c*,*c*-RuCl_2_(CO)_2_(dmso-O)}_2_(4′*cis*DPyP)] (**3**)

Treatment of 4′*cis*DPyP with an excess (typically 6 equiv) of **6** in chloroform at room temperature smoothly afforded ditopic intermediate [{*t,c,c*-RuCl_2_(CO)_2_(dmso-O)}_2_(4′*cis*DPyP)] (**3**) in excellent yield (no unreacted 4′*cis*DPyP detected via TLC within 1 h). After solvent removal, the dark purple solid was washed with water to remove the unreacted ruthenium complex and the released DMSO (the product is soluble in all of the other solvents tested). The washing also removed most of the bound dmso-O, thus affording mainly the aqua species [{*t,c,c*-RuCl_2_(CO)_2_(OH_2_)}_2_(4′*cis*DPyP)] (**3H**_**2**_**O**). The ^1^H NMR spectrum of the crude reaction product in DMSO*-d*_6_ (Supporting Information) presents only one main set of resonances consistent with 4′*cis*DPyP being symmetrically coordinated to two equal Ru(II) complexes; for example, the resonances of both pyridyl rings are equally shifted to higher frequencies compared to the free porphyrin. The spectral features of the Ru fragments confirm that the original geometry remained unchanged (Supporting Information). (i) Consistent with being *trans* to a CO,^48^ the residual dmso-O resonates as a singlet at 2.97 ppm in the NMR spectrum in CDCl_3_; (ii) two clear CO stretching bands are found in the infrared (IR) spectrum at 2072 and 2002 cm^–1^, as expected for a *cis*-{Ru(CO)_2_} fragment.

**Figure 3:**
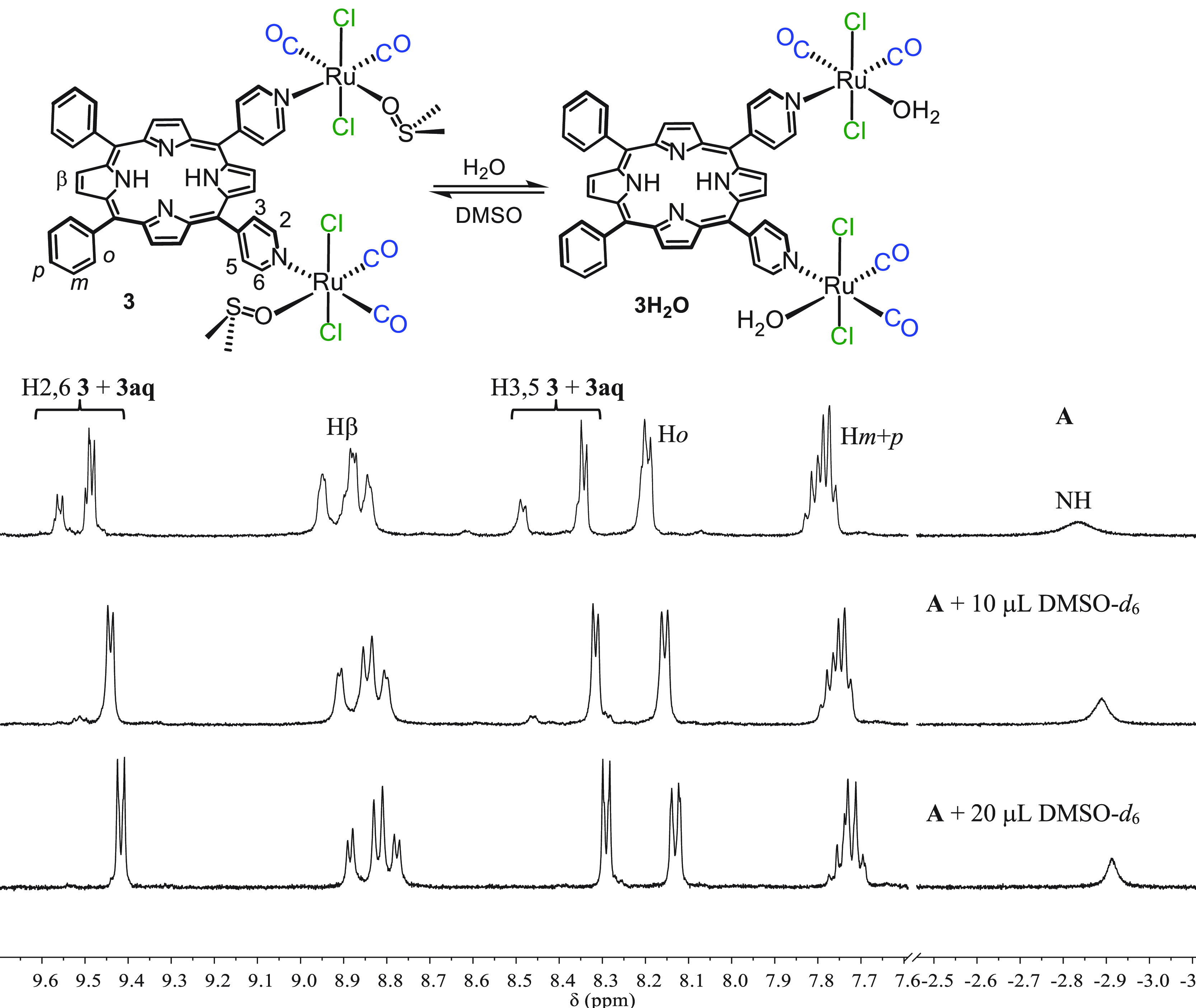
^1^H NMR spectrum of recrystallized **3** in CDCl_3_ (top, A). The other spectra were recorded after progressive addition of 10 μL aliquots of DMSO-*d*_6_ (that also induces the slight shift of all resonances).

Compound **3** can be isolated in almost pure form by dissolving **3H**_**2**_**O** in a chloroform/DMSO mixture followed by precipitation with diethyl ether. Also in this case, the coordinated water is not fully replaced by dmso-O. In fact, the NMR spectrum of this species recorded in CDCl_3_ (Figure 3) shows, in addition to the major set resonances of **3**, a minor set of partially resolved signals, attributed to the residual aqua species **3H**_**2**_**O**. The equilibrium between these two species is shifted toward **3** by the addition of DMSO-*d*_6_, and the resonances of **3H**_**2**_**O** concomitantly disappear (Figure 3). Because from the point of view of the further reactivity **3H**_**2**_**O** and **3** behave equally, and the influence on the MW and stoichiometric ratio of the reactions is marginal, for the sake of brevity in the following no distinction between them will be made and only **3** will be used.

The corresponding ditopic intermediates with 5,10-bis(4′-pyridyl)-15,20-di-*p*-(tolyl)porphyrin (4′*cis*DPyMP, **3Me**) and with 3′*cis*DPyP (**4**) were prepared with the same procedure (Supporting Information).^49^

### Synthesis and Characterization of Heteroleptic 2+2 Metallacycle [{*t,c,c*-RuCl_2_(CO)_2_}_2_(4′*cis*DPyP)(3′*cis*DPyP)] (**5**)

Treatment of 3′*cis*DPyP with a slight excess of **3** at 40 °C in dried CH_2_Cl_2_ afforded heteroleptic 2+2 metallacycle [{*t,c,c*-RuCl_2_(CO)_2_}_2_(4′*cis*DPyP)(3′*cis*DPyP)] (**5**). The reaction was monitored by TLC and stopped when depletion of 3′*cis*DPyP was complete (∼1 h). The crude was purified by flash chromatography, and a single fraction was collected, with an isolated yield of pure product of 26%. To the best of our knowledge, this is the first example of an isolated heteroleptic metallacycle that features two porphyrins with almost indistinguishable binding sites.

**Figure 4:**
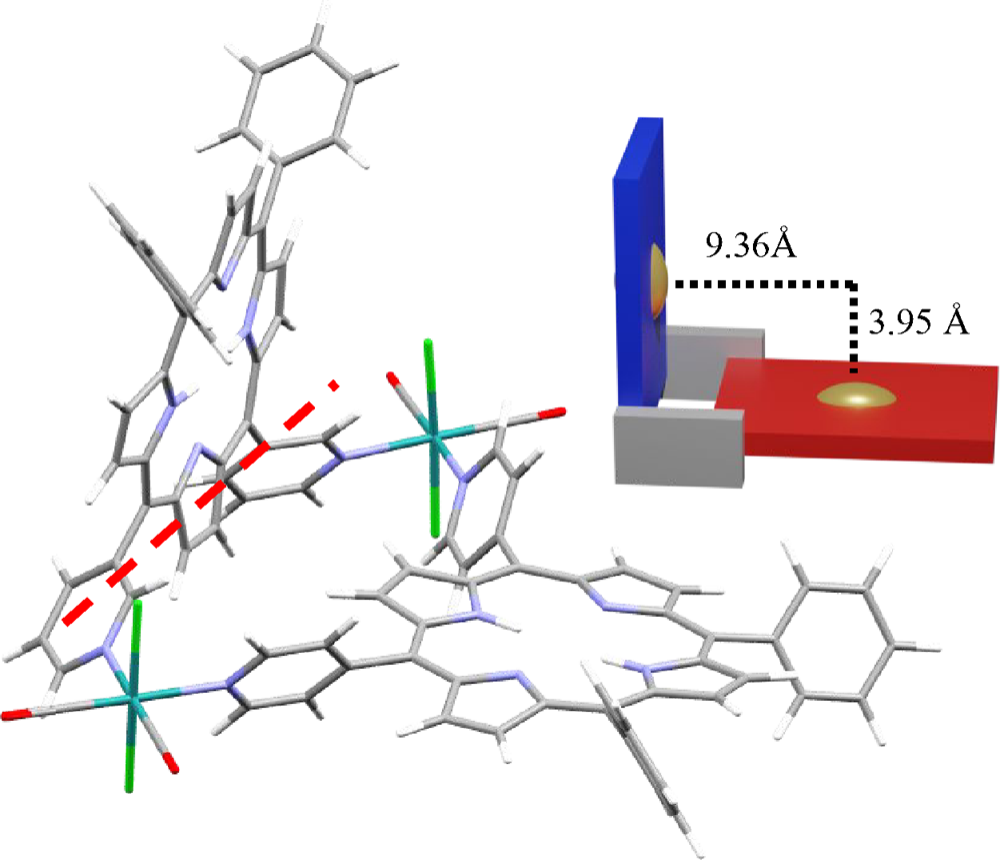
Energy-minimized model of [{*t,c,c*-RuCl_2_(CO)_2_}_2_(4′*cis*DPyP)(3′*cis*DPyP)] (**5**). The red dotted line represents the intersection of the average planes of the two porphyrins. On the right is a schematic representation of **5** as a molecular panel. Color code: red for 4′*cis*DPyP, blue for 3′*cis*DPyP, gray for {*t,c,c*-RuCl_2_(CO)_2_}, and gold for the centroid of the porphyrin.

In the reasonable hypothesis that each porphyrin maintains a coordination geometry similar to that found in homoleptic metallacycles **1** and **2**,^25,26^ i.e., either coplanar (4′*cis*DPyP) or perpendicular (3′*cis*DPyP) to the equatorial coordination plane (N, N, C, and C) of each Ru linker, 2+2 metallacycle **5** is expected to have a folded, L-shaped geometry in which the planes of the two porphyrins are almost orthogonal to one another. Even though we were unable to grow crystals of **5** suitable for X-ray analysis, its energy-minimized model (Figure 4) is perfectly consistent with this hypothesis. The average planes of the porphyrins form a dihedral angle of 92.6°. The model also shows that the 4′*cis*DPyP side of the L is significantly longer than the 3′*cis*DPyP side; in fact, the calculated distance from the intersection between the average planes of the two porphyrins (that falls into a pyrrole ring of 3′*cis*DPyP) to the centroid of 4′*cis*DPyP, 9.36 Å, is more than double that to the centroid of 3′*cis*DPyP, 3.95 Å (Figure 4).

**Figure 5:**
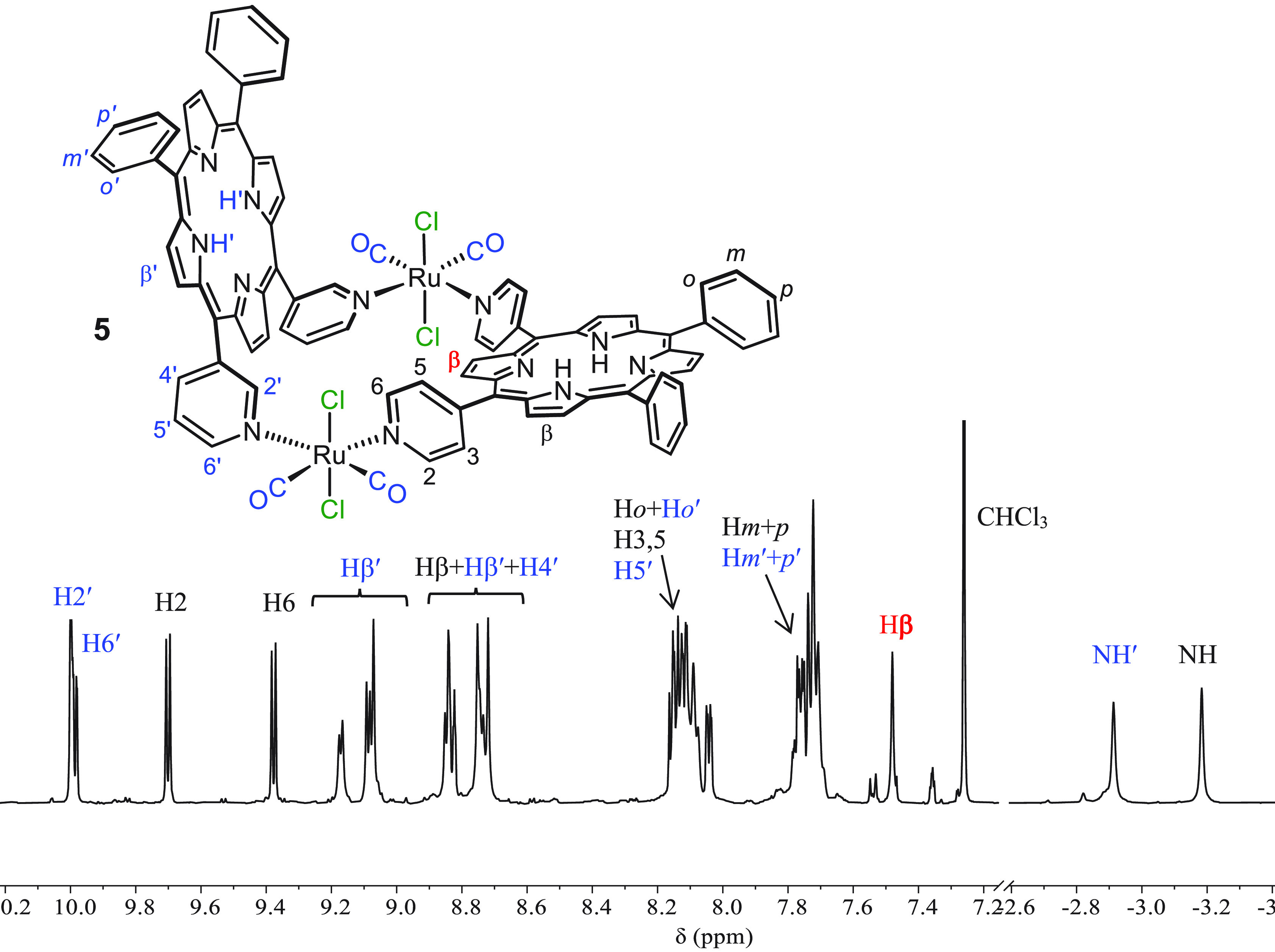
^1^H NMR spectrum (CDCl_3_) of [{*t,c,c*-RuCl_2_(CO)_2_}_2_(4′*cis*DPyP)(3′*cis*DPyP)] (**5**), with the labeling scheme. The protons of 3′*cis*DPyP are labeled in blue.

The ^1^H NMR spectrum of **5** is reported in Figure 5. Assignments were made according to 2D spectra (Supporting Information) and by comparison with homoleptic metallacycles **1** and **2**.

**Figure 6:**
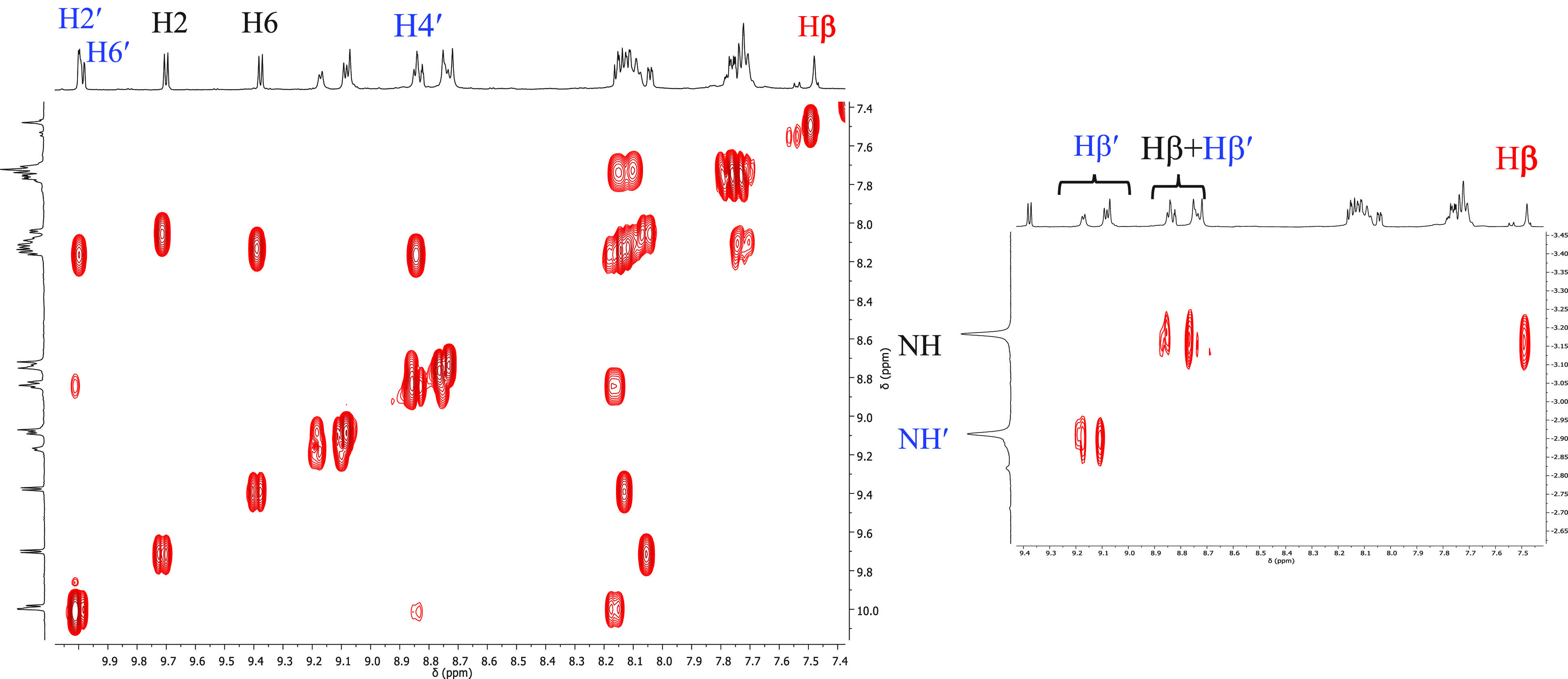
^1^H–^1^H COSY spectrum of **5** (CDCl_3_), with the aromatic region and enlargement showing the cross peaks between βH and NH signals. See Figure 5 for the labeling scheme.

In the high-frequency region, the spectrum displays two sets of equally intense signals typical of 4′*cis*DPyP and 3′*cis*DPyP coordinated in a symmetrical fashion to two {*t,c,c*-RuCl_2_(CO)_2_} fragments. The two doublets at 9.70 and 9.38 ppm were assigned to H2 and H6, respectively, of 4′*cis*DPyP. In fact, the *meso* six-membered rings typically lay approximately perpendicular to the porphyrin ring and are in slow rotation about the C_*meso*_–C_ring_ bond; therefore, when the plane of the porphyrin is not a plane of symmetry for the molecule, as in the case of **5**, the protons on the two sides have distinct resonances. Consistent with this hypothesis, the two doublets are correlated by a clear exchange cross peak in the ^1^H–^1^H ROESY spectrum (Supporting Information). The doublet at 9.38 ppm was attributed to H6, i.e., the proton that in our scheme lays above the average plane of 4′*cis*DPyP and partially falls into the shielding cone of the adjacent 3′*cis*DPyP. Protons H3 and H5 are more distant from the other porphyrin, so their signals [identified through the COSY spectrum (Figure 6)] fall closer to one other, together with other phenyl signals in a multiplet centered at 8.13 ppm.

The resonances of protons H2′ and H6′ of 3′*cis*DPyP, a singlet and a doublet, respectively, are partially overlapped at ∼10 ppm. The resonances of the 16 pyrrole protons (singlets and doublets, 2H each) partially overlap between 8.7 and 9.2 ppm with one notable exception. One singlet falls at 7.47 ppm and was assigned to the two equivalent Hβ protons of 4′*cis*DPyP located between the pyridyl rings [i.e., in positions 7 and 8 (red label in Figure 5)]: they point toward 3′*cis*DPyP and partially fall into its shielding cone. Consistent with the model structure (see above), the corresponding β-protons on 3′*cis*DPyP are more removed from 4′*cis*DPyP and thus less shielded. Lastly, the NH protons of the two porphyrins resonate as two well-resolved sharp singlets (2H each) at −2.91 and −3.18 ppm. On the basis of the consideration of mutual shielding described above, the singlet at −3.18 ppm (which falls at a frequency significantly lower than those of both **1** and **2**) was assigned to 4′*cis*DPyP. Consistently, in the COSY spectrum (Figure 6) it has a cross peak with the Hβ singlet at 7.47 ppm.

The ultraviolet–visible (UV–vis) spectrum of **5** is similar to those of the component porphyrins, and consistent with the nearly orthogonal orientation between the two porphyrins, no exciton splitting is observed in the Soret band (contrary to what is found in **2**).^26^ In the IR spectrum, the two CO stretching bands (2074 and 2014 cm^–1^) are similar to those measured for precursor **3**.

Heteroleptic metallacycle **5** was also successfully prepared (even though on only a small scale, in an NMR tube) following the complementary synthetic procedure, i.e., upon treating reactive ditopic intermediate **4** with 4′*cis*DPyP (Supporting Information).

**Figure 7:**
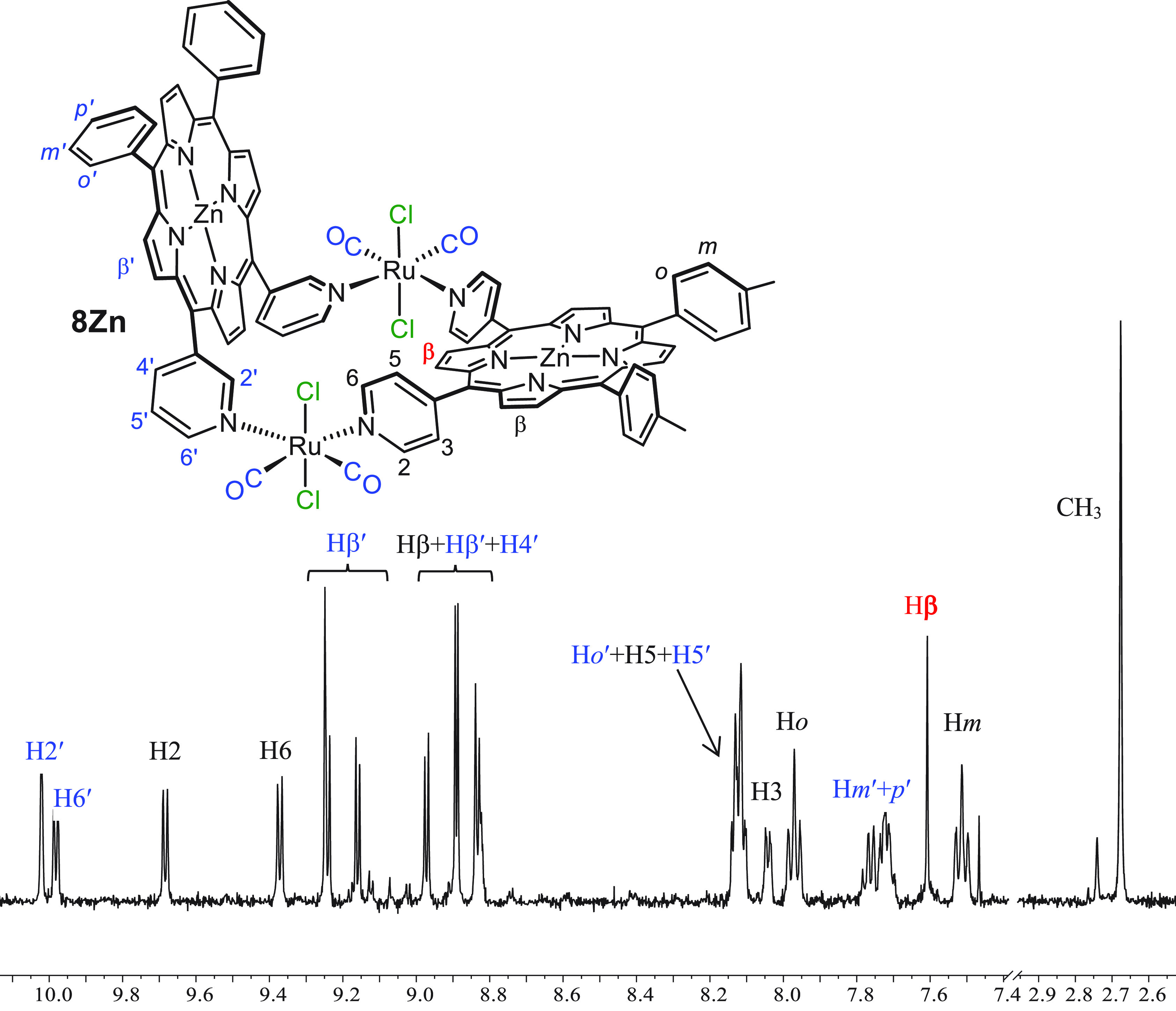
^1^H NMR spectrum (CDCl_3_) of heteroleptic 2+2 metallacycle [{*t*,*c*,*c*-RuCl_2_(CO)_2_}_2_(Zn·4′*cis*DPyMP)(Zn·3′*cis*DPyP)] (**8Zn**) with a labeling scheme. The protons of 3′*cis*DPyP are labeled in blue.

Strictly similar heteroleptic 2+2 metallacycle [{*t,c,c*-RuCl_2_(CO)_2_}_2_(4′*cis*DPyMP)(3′*cis*DPyP)] (**8**), which contains 4′*cis*DPyMP in place of 4′*cis*DPyP, was obtained in 20% isolated yield with the same synthetic approach. Ditopic intermediate [{*t,c,c*-RuCl_2_(CO)_2_(dmso-O)}_2_(4′*cis*DPyMP)] (**3Me**) was treated with a stoichiometric amount of 3′*cis*DPyP in chloroform at 40 °C, followed by flash chromatography purification of the crude product. The ^1^H NMR spectrum of **8** (Supporting Information) is similar to that of **5**, with the addition of the singlet for the methyl groups at 2.65 ppm and a better resolution of the signals in the crowded aromatic region. Treatment of **8** with an excess of zinc acetate in a CHCl_3_/MeOH mixture afforded the corresponding fully zincated metallacycle **8Zn**. The insertion of zinc involves the expected decrease in the number of Q bands in the UV–vis spectrum from four to two (Supporting Information). Even though, aside from the absence of the NH signals, the ^1^H NMR spectrum of **8Zn** in CDCl_3_ is very similar to that of **8** (Figure 7), several resonances are better resolved, for example, those of protons H2′ and H6′ of 3′*cis*DPyP and of the pyrrole protons (see also the Supporting Information).

By virtue of its better resolved ^1^H NMR spectrum, **8Zn** was well suited for a DOSY investigation to obtain more information about the size of these heteroleptic metallacyles (Supporting Information). The measured diffusion coefficient (*D*_t_) for **8Zn** was (6.06 ± 0.10) × 10^–6^ cm^2^ s^–1^, which corresponds to a hydrodynamic radius (*r*_H_) for the molecule of 6.7 Å. These data compare well with those of homoleptic 2+2 molecular square **1Zn**, measured under the same conditions [*D*_t_ = (5.55 ± 0.01) × 10^–6^ cm^2^ s^–1^, and *r*_H_ = 7.3 Å].

Finally, we compared the stepwise synthetic approach with the one-pot preparation, which consists of treating a 1:1 mixture of 4′*cis*DPyMP and 3′*cis*DPyP with 2 equiv of Ru(II) precursor **6**. A careful analysis of the TLC spots of the mixture as well as of the low-frequency region (NH resonances) of the otherwise extremely crowded ^1^H NMR spectrum allowed us to establish that the crude product was a mixture of the three possible 2+2 metallacycles **8**, [*t*,*c*,*c*-RuCl_2_(CO)_2_(4′*cis*DPyMP)]_2_ (**1Me**),^50^ and [*t*,*c*,*c*-RuCl_2_(CO)_2_(3′*cis*DPyP)]_2_ (**2**) in almost equal amounts (in addition to oligomeric species, whose presence is suggested by some broad resonances), as expected for a reaction under kinetic control (Supporting Information). Given the very similar chromatographic mobility of the three metallacycles (Experimental Section), this statistical mixture was not easily amenable to separation by column chromatography. In conclusion, even for this relatively simple 2+2 heteroleptic metallacycle, the one-pot approach turned out to be less suitable than the stepwise one.

## Conclusions

We described a modular stepwise approach for the preparation of heteroleptic metallaycles of porphyrins linked through neutral 90°-angular Ru(II) fragments. As a proof of concept, the synthetic route was successfully applied to the preparation of unprecedented 2+2 heteroleptic metallacycle [{*t,c,c*-RuCl_2_(CO)_2_}_2_(4′*cis*DPyP)(3′*cis*DPyP)] (**5**). The preparation of **5** was accomplished through the isolation of a reactive ditopic intermediate, in which one of the two porphyrins is linked to two ruthenium fragments, each having one residual readily available coordination site (a dmso-O). Thus, metallacycle **5** was obtained under mild conditions through two complementary routes: by treatment of [{*t*,*c*,*c*-RuCl_2_(CO)_2_(dmso-O)}_2_(4′*cis*DPyP)] (**3**) with 1 equiv of 3′*cis*DPyP or, alternatively, by treatment of [{*t,c,c*-RuCl_2_(CO)_2_(dmso-O)}_2_(3′*cis*DPyP)] (**4**) with 1 equiv of 4′*cis*DPyP.

**Figure 8:**
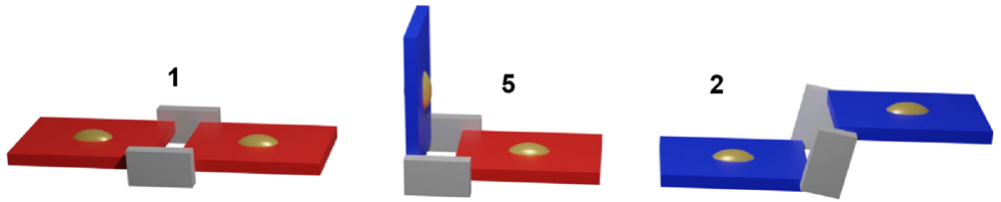
Schematic representation of the 2+2 metallacycles of porphyrins, homoleptic **1** and **2**, and heteroleptic **5**. Color code: red for 4′*cis*DPyP, blue for 3′*cis*DPyP, gray for {*t,c,c*-RuCl_2_(CO)_2_}, and gold for the centroid of the porphyrin.

Compound **5**, which was obtained in pure form in acceptable isolated yield, fills the gap in the series of the corresponding homoleptic metallacycles with 4′*cis*DPyP (**1**) and 3′*cis*DPyP (**2**) (Figure 8). It has an L-shaped geometry, with the 4′*cis*DPyP side of the L being more than twice as long as the 3′*cis*DPyP side.

With the same synthetic approach, the strictly similar [{*t,c,c*-RuCl_2_(CO)_2_}_2_(4′*cis*DPyMP)(3′*cis*DPyP)] (**8**), which contains 4′*cis*DPyMP in place of 4′*cis*DPyP, was obtained. The one-pot approach, tested for this heteroleptic metallacycle, turned out to be unpractical, affording a mixture of **8** with the corresponding homoleptic metallacycles, **1Me** and **2**, of difficult chromatographic separation.

The modular approach that we established is highly flexible and opens the way to several possible exciting developments, such as the preparation of extended metallacycles. For example, treatment of reactive intermediate **3** with 0.5 equiv of a tetrapyridylporphyrin is expected to lead to the formation of 3+4 extended metallacycles (i.e., three porphyrins and four Ru linkers) featuring two different porphyrins. Combinations of 4′- and 3′PyPs will afford unprecedented geometries that, after metalation, like **1** and **2** might be further exploited as molecular panels for the hierarchical construction of 3D assemblies. In addition, because the porphyrins are introduced in a stepwise manner, it is possible, in principle, to place different metal ions in selected positions inside the metallacycle, thus opening the way to currently unavailable perspectives. For example, it might become possible to investigate how the rate of photoinduced processes (e.g., energy or electron transfer) depends not only on the relative orientation of the porphyrins (orthogonal vs parallel) but also on the specific direction (e.g., center to periphery or vice versa).

Lastly, other reactive intermediates, in which the porphyrin has a different geometry (e.g., 4′*trans*DPyP) or bears reactive metal fragments different from {*t,c,c*-RuCl_2_(CO)_2_(dmso-O)} (or both), might be prepared and be used as precursors of new heteroleptic metallacycles.

## Experimental Section

### Materials

The reference compounds [*t*,*c*,*c*-RuCl_2_(CO)_2_(4′*cis*DPyP)]_2_ (**1**) and [*t*,*c*,*c*-RuCl_2_(CO)_2_(3′*cis*DPyP)]_2_ (**2**) were prepared as described in refs (25) and (26). [*t*,*c*,*c*-RuCl_2_(CO)_2_(4′*cis*DPyMP)]_2_ (**1Me**) was prepared and purified following the same procedure described in ref (25) for **1**. Crystals suitable for X-ray analysis were obtained by layering *n*-hexane on top of a CH_2_Cl_2_ solution of **1Me**. Elemental analysis for [*t*,*c*,*c*-RuCl_2_(CO)_2_(4′*cis*DPyMP)]_2_·2CH_2_Cl_2_·7.3H_2_O (**1M**e·2CH_2_Cl_2_·7.3H_2_O) and [C_92_H_64_Cl_4_N_12_O_4_Ru_2_]·2CH_2_Cl_2_·7.3H_2_O (2046.9). Calcd: C, 55.16; H, 4.07; N, 8.21. Found: C, 54.93; H, 4.16; N, 8.16. ^1^H NMR (CDCl_3_): δ 9.81 (d, 8H, H2,6), 9.09 (d, 4H, Hβ), 9.03 (d, 4H, Hβ), 9.03 (s, 4H, Hβ), 8.92 (s, 4H, Hβ), 8.57 (d, 8H, H3,5), 8.14 (d, 8H, H*o*), 7.63 (d, 8H, H*m*), 2.76 (s, 12H, Me), −2.72 (s, 4H, NH).

### Instrumental Methods

Mono- and bidimensional (^1^H–^1^H COSY and ^1^H–^13^C HSQC) NMR spectra were recorded at room temperature, unless stated otherwise, on a Varian 400 or 500 spectrometer (^1^H, 400 or 500 MHz; ^13^C{^1^H}, 100.5 or 125.7 MHz). ^1^H DOSY experiments were performed on the Varian 500 spectrometer at −5 °C (CDCl_3_), using the Bipolar Pulse Pair Stimulated Echo with Convection Compensation Sequence implemented in the VnmrJ software. ^1^H chemical shifts were referenced to the peak of residual nondeuterated solvent (δ 7.26 for CDCl_3_, δ 5.32 for CD_2_Cl_2_). ESI mass spectra were recorded in positive mode on a PerkinElmer APII spectrometer at 5600 eV. However, as is typical for such neutral systems,^45^ the ESI-MS spectra of the model complexes and metallacycles of porphyrins showed only peaks deriving from the fragmentation. The UV–vis spectra were recorded on an Agilent Cary 60 spectrophotometer, using 1.0 cm path-length quartz cuvettes (3.0 mL). Infrared spectra of chloroform solutions in the CO stretching region were recorded between CaF_2_ windows (0.5 mm spacer) on a PerkinElmer Fourier-transform IR/Raman 2000 instrument in transmission mode. Elemental analyses were performed on a Thermo Flash 2000 CHNS/O analyzer in the Department of Chemistry of the University of Bologna (Bologna, Italy).^45^

### X-ray Diffraction

Data were collected at the X-ray diffraction beamline (XRD1) of the Elettra Synchrotron of Trieste, Italy, equipped with a Pilatus 2M image plate detector. The collection temperature was 100 K (nitrogen stream supplied through an Oxford Cryostream 700 instrument); the wavelength of the monochromatic X-ray beam was 0.700 Å, and the diffractograms were obtained with the rotating crystal method. The crystals were dipped in N-paratone and mounted on the goniometer head with a nylon loop. The diffraction data were indexed, integrated, and scaled using the XDS code.^51^ The structure was determined by the dual-space algorithm implemented in the SHELXT code.^52^ Fourier analysis and refinement were performed by the full-matrix least-squares methods based on *F*^2^ implemented in SHELXL.^53^ The Coot and SHELXLE programs were used for modeling.^54,55^ Anisotropic thermal motion was allowed for all non-hydrogen atoms. Hydrogen atoms were placed at calculated positions with isotropic factors *U* = 1.2*U*_eq_, where *U*_eq_ is the equivalent isotropic thermal factor of the bonded non-hydrogen atom. Crystal data and details of refinements are given in Supporting Information.

### Computational Methods

The model of compound **5** was set up by connecting the corresponding fragments taken from existing published X-ray structures of **1** and **2**.^26^ The so obtained starting geometry was then relaxed with the Quantum-Espresso suite of codes,^56,57^ in the frame of density functional theory with the Kohn–Sham orbitals expanded in plane waves and the effects of atomic core regions accounted for by projector-augmented wave (PAW) pseudopotentials.^58^ The exchange-correlation part of the energy functional was modeled with the (spin-unpolarized) generalized gradient approximation (GGA), in the PBE parametrization.^59^ The plane wave expansion of the crystalline orbitals was truncated at a cutoff energy of 80 Ry (as determined by convergence of the total energy with a stepwise increase in the cutoff), and a corresponding cutoff of 800 Ry was used for the expansion of charge density and potential. Due to the large unit cell used for the calculation, the first Brillouin zone was sampled at the Γ point only. A “molecule in the box” methodology was applied, where a single molecule is (periodically) simulated in a unit cell, large enough to minimize any interactions between the molecule and its periodic images. A cubic cell with a cell constant of 31.00 Å was found to give a minimum separation distance of >10 Å between nearest atoms of any two contiguous periodic images. The correction of Makov and Payne to the total energy for isolated systems when simulated with large unit cells, as implemented in the Quantum-Espresso suite, was also applied.^60^ Convergence thresholds for geometry relaxation were 1 × 10^–4^ Ry for total energy and 1 × 10^–3^ Ry/Å for the maximum force component acting on atoms.

### Synthesis of the Complexes

#### [{*t,c,c*-RuCl_2_(CO)_2_(OH_2_)}_2_(4′*cis*DPyP)] (**3H**_**2**_**O**)

To a 87.0 mg (0.486 mmol) amount of *t,c,c*-[RuCl_2_(CO)_2_(dmso-O)_2_] (**6**), dissolved in 20 mL of CHCl_3_, was added 50.0 mg (0.081 mmol) of 4′*cis*DPyP (**6**:4′*cis*DPyP ratio of 6). The purple solution was stirred at room temperature for 50 min, and afterward, the solvent was removed under reduced pressure. The recovered purple solid was washed with H_2_O to remove the unreacted complex and the free DMSO and dried under vacuum. The solid was redissolved in chloroform and dried on Na_2_SO_4_. The solution was recovered, and the solvent was removed by evaporation at reduced pressure and dried under vacuum. Yield: 89.6 mg, 98%. ^1^H NMR (DMSO-*d*_6_): δ 9.41 (d, 4H, H2,6), 8.88 (d, 2H, Hβ), 8.82 (s, 2H, Hβ), 8.80 (s, 2H, Hβ), 8.77 (d, 2H, Hβ), 8.28 (d, 4H, H3,5), 8.12 (d, 4H, Ho), 7.72 (m, 6H, Hm+p), −2.92 (s, 2H, NH).

The dmso compound [{*t,c,c*-RuCl_2_(CO)_2_(dmso-O)}_2_(4′*cis*DPyP)] (**3**) was obtained by dissolving 50.0 mg of the product in 10 mL of CHCl_3_ in the presence of anhydrous Na_2_SO_4_. The mixture was stirred for 10 min and then filtered, and 500 μL of DMSO was added to the mother liquor. The solution was concentrated to 5 mL, and complex **3** was precipitated by addition of diethyl ether. After recrystallization, the ^1^H NMR spectrum of **4** (CDCl_3_) shows the dmso-O singlet at 2.97 ppm. UV–vis [CHCl_3_; λ_max_ (nm), relative intensity (%)]: 425 (100, Soret band), 519 (5.97), 556 (2.96), 591 (2.39), 647 (1.94). IR (selected bands in CHCl_3_, cm^–1^): 2072 (νCO), 2002 (νCO).

#### [{*t,c,c*-RuCl_2_(CO)_2_(OH_2_)}_2_(4′*cis*DPyMP)] (**3Me**)

The procedure is similar to that leading to **3**. To a 178.8 mg (0.465 mmol) amount of **6**, dissolved in 20 mL of CHCl_3_, was added 50.0 mg (0.078 mmol) of 4′*cis*DPyMP (**6**:4′*cis*DPyMP ratio of 6), and the purple solution was stirred for 50 min at room temperature. The solvent was removed under reduced pressure, and the purple solid was washed with water and dried under vacuum. The solid was redissolved in chloroform and dried with Na_2_SO_4_. Yield: 71.2 mg, 81%. ^1^H NMR (DMSO-*d*_6_): δ 9.37 (d, 4H, H2,6), 8.92 (m, 8H, Hβ), 8.62 (d, 4H, H3, H5), 8.12 (d, 4H, Ho), 7.66 (d, 4H, Hm), 2.68 (s, 6H, Me), −2.95 (s, 2H, NH). UV–vis [CHCl_3_; λ_max_ (nm), relative intensity (%)]: 424 (100, Soret band), 526 (5.97), 560 (2.96), 600 (2.39), 650 (1.94). IR (selected bands in CHCl_3_, cm^–1^): 2072 (νCO), 2002 (νCO).

#### [{*t,c,c*-RuCl_2_(CO)_2_(OH_2_)}_2_(3′*cis*DPyP)] (**4**)

To a 186.9 mg (0.486 mmol) amount of **6**, dissolved in 50 mL of CHCl_3_, was added 50.0 mg (0.081 mmol) of 3′*cis*DPyP (**6**:3′*cis*DPyP ratio of 6), and the purple solution was stirred at room temperature for 50 min; then, the solvent was removed under reduced pressure. The recovered purple solid was washed with H_2_O to remove the unreacted complex and the free DMSO and dried under vacuum. The solid was redissolved in chloroform and dried on Na_2_SO_4_. The solution was recovered, the solvent removed by evaporation at reduced pressure, and the residue dried under vacuum. Yield: 84.7 mg, 94%. ^1^H NMR (CDCl_3_ + DMSO-*d*_6_): δ 9.67, 9.62 (2s, 2H, H2), 9.24 (d, 2H, H6), 8.68 (m, 8H, Hβ), 8.55 (m, 2H, H4), 7.97 (m, 4H, Ho), 7.78 (t, 2H, H5), 7.56 (m, 6H, Hm+p), −3.08 (s, 2H, NH). IR (selected bands in CHCl_3_, cm^–1^): 2073 (νCO), 2003 (νCO).

#### [{*t,c,c-*RuCl_2_(CO)_2_}_2_(4′*cis*DPyP)(3′*cis*DPyP)] (**5**)

To a 15.0 mg (0.014 mmol) amount of **3**, dissolved in 4 mL of anhydrous CH_2_Cl_2_ and 50 μL of DMSO, was added 5.6 mg (0.009 mmol) of 3′*cis*DPyP (**3**:3′*cis*DPyP ratio of 1.5). The purple solution was stirred at 40 °C for 50 min. The solvent was removed under reduced pressure, and the purple solid was washed first with H_2_O, then with MeOH, and finally with diethyl ether; part of the raw material, most likely attributable to oligomeric open-chain species, remained stuck on the top of the column as a purple band. The solid was dried under vacuum and purified by chromatography on a short column (silica gel, CHCl_3_). A single fraction, containing the desired product, was collected (TLC, CHCl_3_, *R_f_* = 0.31). Yield: 4 mg, 26%. ^1^H NMR (CDCl_3_; the protons of 3′*cis*DPyP are primed): δ 9.99 (m, 2H, H2′+H6′), 9.70 (d, 2H, H2), 9.38 (d, 2H, H6), 9.17 (d, 2H, Hβ), 9.08 (s+d, 4H, Hβ), 8.83 (m, 4H, Hβ′+H4′), 8.74 (m, 6H, Hβ′), 8.13 (m, 8H, H5+Ho+Ho′), 8.04 (m, 2H, H3), 7.74 (m, 12H, Hm+Hp+Hm′+Hp′), 7.48 (s, 2H, Hβ′), −2.91 (s, 2H, NH′), −3.18 (s, 2H, NH). UV–vis [CHCl_3_; λ_max_ (nm), relative intensity (%)]: 423 (100, Soret band), 520 (6.83), 555 (3.37), 592 (2.61), 648 (1.76). IR (selected bands in CHCl_3_, cm^–1^): 2074 (νCO), 2014 (νCO). TLC (CHCl_3_): *R_f_* = 0.31.

#### [{*t,c,c-*RuCl_2_(CO)_2_}_2_(4′*cis*DPyMP)(3′*cis*DPyP)] (**8**)

To a 50.0 mg amount of **3Me** (0.044 mmol), dissolved in 13 mL of CHCl_3_ and 0.17 mL of DMSO, was added 21.0 mg (0.034 mmol) of 3′*cis*DPyP (**3Me**:3′*cis*DPyP ratio of 1.3). The purple solution was stirred at 40 °C for 50 min, then the solvent removed under reduced pressure, and the crude purple solid washed first with H_2_O, then with MeOH, and finally with diethyl ether. The crude was purified by column chromatography (silica gel CHCl_3_:*n*-hexane ratio of 94:6), collecting a fraction that, according to TLC analysis (silica gel, CHCl_3_), contained three species with *R_f_* values of 0.23, 0.15, and 0.09, respectively. This fraction was purified through another chromatographic column (silica gel, CHCl_3_:*n*-hexane ratio of 80:20) collecting two fractions: the first containing 2+2 homoleptic metallacycle **1Me** (TLC, *R_f_* = 0.23) and the second containing **8** (TLC, *R_f_* = 0.15). Yield: 12 mg, 20%. ^1^H NMR (CDCl_3_; the protons of 3′*cis*DPyP are primed): δ 9.99 (s, 2H, H2′+H6′), 9.69 (d, 2H, H2), 9.36 (d, 2H, H6), 9.17 (d, 2H, Hβ′), 9.08 (d, 2H, Hβ′), 9.07 (s, 2H, Hβ′), 8.86 (d, 2H, Hβ), 8.83 (dt, 2H, H4′), 8.77 (s, 2H, Hβ), 8.72 (m, 4H, Hβ′+Hβ), 8.13 (m, 8H, H5′+H5+Ho′), 8.04 (dd, 2H, H3), 7.97 (m, 4H, Ho), 7.74 (m, 6H, Hm′+Hp′), 7.51 (m, 4H, Hm), 7.47 (s, 2H, Hβ), 2.67 (s, 6H, Me), −2.92 (s, 2H, NH′), −3.19 (s, 2H, NH). UV–vis [CHCl_3_; λ_max_ (nm), relative intensity (%)]: 423 (100, Soret band), 520 (6.45), 555 (2.97), 591 (2.22), 647 (1.32). IR (selected bands in CHCl_3_, cm^–1^): 2075 (νCO), 2015 (νCO).

#### [{*t*,*c*,*c*-RuCl_2_(CO)_2_}_2_(Zn·4′*cis*DPyMP)(Zn·3′*cis*DPyP)] (**8Zn**)

To a 12.0 mg (0.0096 mmol) amount of **8** dissolved in 20 mL of CHCl_3_ was added a 6.5 mg amount (0.030 mmol) of Zn(CH_3_COO)_2_·2H_2_O, dissolved in 1 mL of MeOH (CHCl_3_:MeOH ratio of 20, Zn:**8** ratio of 4). The purple solution was stirred at room temperature in the dark for 48 h. The solvent was removed under reduced pressure, and the purple solid was washed with H_2_O, then with MeOH, and finally with diethyl ether and dried under vacuum (TLC, CHCl_3_, *R_f_* = 0.17). Yield: 6.4 mg, 50%. ^1^H NMR (CDCl_3_; the protons of 3′*cis*DPyP are primed): δ 10.20 (s, 2H, H2′), 9.98 (m, 2H, H6′), 9.69 (d, 2H, H6), 9.37 (d, 2H, H2), 9.24 (s, 2H, Hβ), 9.24 (d, 2H, Hβ), 6.19 (d, 2H, Hβ), 8.97 (d, 2H, Hβ′), 8.89 (s, 2H, Hβ), 8.89 (s, 2H, Hβ′), 8.83 (d, 2H, Hβ′), 8.83 (m, 2H, H4′), 8.12 (m, 8H, H5+H5′+Ho′), 8.04 (m, 2H, H3), 7.97 (m, 4H, Ho), 7.74 (m, 6H, Hm′+Hp′), 7.61 (s, 2H, Hβ), 7.52 (m, 4H, Hm), 2.68 (s, 6H, Me). UV–vis [CHCl_3_; λ_max_ (nm), relative intensity (%)]: 427 (100, Soret band), 559 (6.97), 600 (2.39).

#### One-Pot Synthesis of [{*t,c,c-*RuCl_2_(CO)_2_}_2_(4′*cis*DPyMP)(3′*cis*DPyP)] (**8**)

The reaction was carried out on a small scale in an NMR tube. A 1.2 mg amount (3.1 × 10^–3^ mmol) of **6** was dissolved in 700 μL of CDCl_3_, and 1.0 mg of 4′*cis*DPyMP (1.5 × 10^–3^ mmol) and 0.9 mg of 3′*cis*DPyP (1.5 × 10^–3^ mmol) (4′*cis*DPyMP:**6**:3′*cis*DPyP ratio of 1:2:1) were added. The purple solution was monitored by ^1^H NMR during a 24 h period at room temperature. At the end of the reaction, the solvent was removed by evaporation at reduced pressure and the purple solid obtained was thoroughly washed with diethyl ether to remove DMSO (it was partially soluble in the most suitable acetone). TLC analysis (silica gel, CHCl_3_) showed the presence of multiple spots, the most intense belonging to **1Me** (*R_f_* = 0.23), **8** (*R_f_* = 0.15), and **2** (*R_f_* = 0.13). The presence of the three metallacycles in almost equal amounts was confirmed by the diagnostic signals of the NH protons in the CDCl_3_^1^H NMR spectrum of the mixture: **1Me** (−2.70 ppm), **8** (−2.90 and −3.17 ppm), and **2** (−3.00 ppm).

## Supporting Information Available

Additional comments on reactive intermediate **7**, one- and two-dimensional NMR spectra, and IR and UV–vis spectra for the reported compounds (PDF)

## Accession Codes

CCDC 2078029 contains the supplementary crystallographic data for this paper. These data can be obtained free of charge via www.ccdc.cam.ac.uk/data_request/cif, or by emailing data_request@ccdc.cam.ac.uk, or by contacting The Cambridge Crystallographic Data Centre, 12 Union Road, Cambridge CB2 1EZ, UK; fax: +44 1223 336033.

## Notes

The University of Trieste is a partner of the CRUI/ACS 2020–2023 agreement.

## References

[ref1] Percástegui E. G.; Jancik V. Coordination-driven assemblies based on *meso*-substituted porphyrins: Metal-organic cages and a new type of *meso*-metallaporphyrin macrocycles. Coord. Chem. Rev. 2020, 407, 213165 10.1016/j.ccr.2019.213165.

[ref2] Durot S.; Taesch J.; Heitz V. Multiporphyrinic Cages: Architectures and Functions. Chem. Rev. 2014, 114, 8542–8578. 10.1021/cr400673y. 25026396

[ref3] Iengo E.; Cavigli P.; Milano D.; Tecilla P. Metal mediated self-assembled porphyrin metallacycles: Synthesis and multipurpose applications. Inorg. Chim. Acta 2014, 417, 59–78. 10.1016/j.ica.2014.02.018.

[ref4] Wytko J. A.; Ruppert R.; Jeandon C.; Weiss J. Metal-mediated linear self-assembly of porphyrins. Chem. Commun. 2018, 54, 1550–1558. 10.1039/C7CC09650J. 29363684

[ref5] Ercolani G. Thermodynamics of Metal-Mediated Assemblies of Porphyrins. In Non-Covalent Multi-Porphyrin Assemblies; Alessio E., Ed.; Structure and Bonding Series; Springer: Berlin, 2006; Vol. 121. 10.1007/430_019

[ref6] Hupp J. T. Rhenium-Linked Multiporphyrin Assemblies: Synthesis and Properties. In Non-Covalent Multi-Porphyrin Assemblies; Alessio E., Ed.; Structure and Bonding Series; Springer: Berlin, 2006; Vol. 121. 10.1007/430_027

[ref7] Bar A. K.; Chakrabarty R.; Mostafa G.; Mukherjee P. S. Self-Assembly of a Nanoscopic Pt_12_Fe_12_ Heterometallic Open Molecular Box Containing Six Porphyrin Walls. Angew. Chem., Int. Ed. 2008, 47, 8455–8459. 10.1002/anie.200803543. 18830951

[ref8] Hernández L. P.; González-Álvarez A.; Oliva A. I.; Ballester P. Metal-mediated multiporphyrin functional assemblies. J. Porphyrins Phthalocyanines 2009, 13, 481–493. 10.1142/S1088424609000693.

[ref9] Meng W. J.; Breiner B.; Rissanen K.; Thoburn J. D.; Clegg J. K.; Nitschke J. R. A Self-Assembled M_8_L_6_ Cubic Cage that Selectively Encapsulates Large Aromatic Guests. Angew. Chem., Int. Ed. 2011, 50, 3479–3483. 10.1002/anie.201100193. 21394866

[ref10] Bar A. K.; Mohapatra S.; Zangrando E.; Mukherjee P. S. A Series of Trifacial Pd_6_ Molecular Barrels with Porphyrin Walls. Chem. - Eur. J. 2012, 18, 9571–9579. 10.1002/chem.201201077. 22744754

[ref11] Ronson T. K.; Zarra S.; Black S. P.; Nitschke J. R. Metal–organic container molecules through subcomponent self-assembly. Chem. Commun. 2013, 49, 2476–2490. 10.1039/c2cc36363a. 23289097

[ref12] Wood D. M.; Meng W.; Ronson T. K.; Stefankiewicz A. R.; Sanders J. K.; Nitschke J. R. Guest-induced transformation of a porphyrin-edged Fe^II^_4_L_6_ capsule into a Cu^I^Fe^II^_2_L_4_ fullerene receptor. Angew. Chem., Int. Ed. 2015, 54, 3988–3992. 10.1002/anie.201411985. 25655272

[ref13] Rizzuto F. J.; Nitschke J. R. Stereochemical plasticity modulates cooperative binding in a Co^II^_12_L_6_ cuboctahedron. Nat. Chem. 2017, 9, 903–908. 10.1038/nchem.2758. 28837174

[ref14] Zhang D.; Ronson T. K.; Nitschke J. R. Functional Capsules via Subcomponent Self-Assembly. Acc. Chem. Res. 2018, 51, 2423–2436. 10.1021/acs.accounts.8b00303. 30207688

[ref15] Rizzuto F. J.; Ramsay W. J.; Nitschke J. R. Otherwise Unstable Structures Self-Assemble in the Cavities of Cuboctahedral Coordination Cages. J. Am. Chem. Soc. 2018, 140, 11502–11509. 10.1021/jacs.8b07494. 30114908

[ref16] Abbreviations: 4′MPyP, 5-(4′-pyridyl)-10,15,20-triphenylporphyrin; 4′*cis*DPyP, 5,10-bis(4′-pyridyl)-15,20-diphenylporphyrin; 4′*cis*DPyMP, 5,10-bis(4′-pyridyl)-15,20-di-*p*-(tolyl)porphyrin; 3′*cis*DPyP, 5,10-bis(3′-pyridyl)-15,20-diphenylporphyrin; 4′TrPyP, 5,10,15-tris(4′-pyridyl)-20-phenylporphyrin; 4′TPyP, 5,10,15,20-tetrapyridylporphyrin. For the sake of brevity, abbreviated versions of the geometrical descriptors for the metal centers are used in the formulas: *c* for *cis* and *t* for *trans*.

[ref17] Drain C. M.; Lehn J.-M. Self-assembly of Square Multiporphyrin Arrays by Metal Ion Coordination. J. Chem. Soc., Chem. Commun. 1994, 2313–2315. 10.1039/c39940002313.

[ref18] Slone R. V.; Hupp J. T. Synthesis, Characterization, and Preliminary Host–Guest Binding Studies of Porphyrinic Molecular Squares Featuring *fac*-Tricarbonylrhenium(I) Chloro Corners. Inorg. Chem. 1997, 36, 5422–5423. 10.1021/ic9703991.

[ref19] Stang P. J.; Fan J.; Olenyuk B. Molecular architecture via coordination: self-assembly of cyclic cationic porphyrin aggregates via transition-metal bisphosphane auxiliaries. Chem. Commun. 1997, 1453–1454. 10.1039/a700506g.

[ref20] Schmitz M.; Leininger S.; Fan J.; Arif A. M.; Stang P. J. Preparation and Solid-State Properties of Self-Assembled Dinuclear Platinum(II) and Palladium(II) Rhomboids from Carbon and Silicon Tectons. Organometallics 1999, 18, 4817–4824. 10.1021/om990567s.

[ref21] Fan J.; Whiteford J. A.; Olenyuk B.; Levin M. D.; Stang P. J.; Fleischer E. B. Preparation and Solid-State Properties of Self-Assembled Dinuclear Platinum(II) and Palladium(II) Rhomboids from Carbon and Silicon Tectons. J. Am. Chem. Soc. 1999, 121, 2741–2752. 10.1021/ja9839825.

[ref22] Fujita N.; Biradha K.; Fujita M.; Sakamoto S.; Yamaguchi K. A porphyrin prism: Structural switching triggered by guest inclusion. Angew. Chem., Int. Ed. 2001, 40, 1718–1721. 10.1002/1521-3773(20010504)40:9<1718::AID-ANIE17180>3.0.CO;2-7. 11353490

[ref23] Iengo E.; Zangrando E.; Alessio E. Synthetic Strategies and Structural Aspects of Metal-Mediated Multi-Porphyrin Assemblies. Acc. Chem. Res. 2006, 39, 841–851. 10.1021/ar040240+. 17115724

[ref24] Casanova M.; Zangrando E.; Iengo E.; Alessio E.; Indelli M. T.; Scandola F.; Orlandi M. Structural and Photophysical Characterization of Multichromophoric Pyridylporphyrin-Rhenium(I) Conjugates. Inorg. Chem. 2008, 47, 10407–10418. 10.1021/ic800971e. 18947175

[ref25] Iengo E.; Zangrando E.; Minatel R.; Alessio E. Metallacycles of porphyrins as building blocks in the construction of higher order assemblies through axial coordination of bridging ligands: solution and solid state characterization of molecular sandwiches and molecular wires. J. Am. Chem. Soc. 2002, 124, 1003–1013. 10.1021/ja016162s. 11829609

[ref26] Iengo E.; Zangrando E.; Bellini M.; Alessio E.; Prodi A.; Chiorboli C.; Scandola F. Pyridylporphyrin metallacycles with a slipped cofacial geometry: spectroscopic, X-ray and photophysical characterization. Inorg. Chem. 2005, 44, 9752–9762. 10.1021/ic051210l. 16363844

[ref27] Iengo E.; Gatti T.; Zangrando E.; Indelli M. T.; Scandola F.; Alessio E. Concerted motions in supramolecular systems: metal-mediated assemblies of porphyrins that behave like nanometric step-machines. Chem. Commun. 2011, 47, 1616–1618. 10.1039/C0CC03513K. 21109900

[ref28] Alessio E.; Casanova M.; Zangrando E.; Iengo E. Modular self-assembled multiporphyrin cages with tunable shape. Chem. Commun. 2012, 48, 5112 10.1039/c2cc31420g. 22514084

[ref29] Iengo E.; Milani B.; Zangrando E.; Geremia S.; Alessio E. Novel Ruthenium Building Blocks for the Efficient Modular Construction of Heterobimetallic Molecular Squares of Porphyrins. Angew. Chem., Int. Ed. 2000, 39, 1096–1099. 10.1002/(SICI)1521-3773(20000317)39:6<1096::AID-ANIE1096>3.0.CO;2-A. 10760932

[ref30] Vidal A.; Battistin F.; Balducci G.; Demitri N.; Iengo E.; Alessio E. The rare example of stereoisomeric 2 + 2 metallacycles of porphyrins featuring chiral-at-metal octahedral ruthenium corners. Inorg. Chem. 2019, 58, 7357–7367. 10.1021/acs.inorgchem.9b00487. 31090413

[ref31] Drain C. M.; Nifiatis F.; Vasenko A.; Batteas J. D. Porphyrin Tessellation by Design: Metal-Mediated Self-Assembly of Large Arrays and Tapes. Angew. Chem., Int. Ed. 1998, 37, 2344–2347. 10.1002/(SICI)1521-3773(19980918)37:17<2344::AID-ANIE2344>3.0.CO;2-B. 29710946

[ref32] Cheng K. F.; Thai N. A.; Grohmann K.; Teague L. C.; Drain C. M. Tessellation of Porphyrazines with Porphyrins by Design. Inorg. Chem. 2006, 45, 6928–6932. 10.1021/ic060448m. 16903751

[ref33] In a somehow symmetrical approach, we described 2+2 cationic metallacycle [Pd(dppp)(4′*cis*TPyP[Ru]_2_)]_2_(CF_3_SO_3_)_4_ in which two adjacent pyridyl rings of each 4′TPyP are bound to the Pd corners and the other two are decorated with neutral Ru complexes {[Ru] = *c,c,c*-[RuCl_2_(dmso-S)_2_(CO)]}: Iengo E.; Minatel R.; Milani B.; Marzilli L. G.; Alessio E. Metal-mediated self-assembly of molecular squares of porphyrins rimmed with coordination compounds. Eur. J. Inorg. Chem. 2001, 2001, 609–612. 10.1002/1099-0682(200103)2001:3<609::AID-EJIC609>3.0.CO;2-V.

[ref34] De S.; Mahata K.; Schmittel M. Metal-coordination-driven dynamic heteroleptic architectures. Chem. Soc. Rev. 2010, 39, 1555–1575. 10.1039/b922293f. 20419210

[ref35] Samanta S. K.; Samanta D.; Bats J. W.; Schmittel M. DABCO as a dynamic hinge between cofacial porphyrin panels and its tumbling inside a supramolecular cavity. J. Org. Chem. 2011, 76, 7466–7473. 10.1021/jo201252q. 21863790

[ref36] Saha S.; Biswas P. K.; Schmittel M. Reversible Interconversion of a Static Metallosupramolecular Cage Assembly into a High-Speed Rotor: Stepless Adjustment of Rotational Exchange by Nucleophile Addition. Inorg. Chem. 2019, 58, 3466–3472. 10.1021/acs.inorgchem.8b03567. 30789716

[ref37] Sun Y.; Chen C.; Liu J.; Stang P. J. Recent developments in the construction and applications of platinum-based metallacycles and metallacages via coordination. Chem. Soc. Rev. 2020, 49, 3889–3919. 10.1039/D0CS00038H. 32412574 PMC7846457

[ref38] Han Y.-F.; Lin Y.-J.; Weng L.-H.; Berke H.; Jin G.-X. Stepwise formation of ‘’organometallic boxes’’ with half-sandwich Ir, Rh and Ru fragments. Chem. Commun. 2008, 350–352. 10.1039/B711809K. 18399204

[ref39] Barry N. P. E.; Austeri M.; Lacour J.; Therrien B. Highly Efficient NMR Enantiodiscrimination of Chiral Octanuclear Metalla-Boxes in Polar Solvent. Organometallics 2009, 28, 4894–4897. 10.1021/om900461s.

[ref40] Garcia-Simon C.; Garcia-Borras M.; Gomez L.; Garcia-Bosch I.; Osuna S.; Swart M.; Luis J. M.; Rovira C.; Almeida M.; Imaz I.; Maspoch D.; Costas M.; Ribas X. Self-Assembled Tetragonal Prismatic Molecular Cage Highly Selective for Anionic π Guests. Chem. - Eur. J. 2013, 19, 1445–1456. 10.1002/chem.201203376. 23212936

[ref41] Garcia-Simon C.; Garcia-Borras M.; Gomez L.; Parella T.; Osuna S.; Juanhuix J.; Imaz I.; Maspoch D.; Costas M.; Ribas X. Sponge-like molecular cage for purification of fullerenes. Nat. Commun. 2014, 5, 5557 10.1038/ncomms6557. 25424201

[ref42] Wang Y.; Ang P. L.; Wong C.-Y.; Yip J. H. K. Gold-Clip-Assisted Self-Assembly and Proton-Coupled Expansion–Contraction of a Cofacial Fe^III^–Porphyrin Cage. Chem. - Eur. J. 2018, 24, 18623–18628. 10.1002/chem.201803501. 30218534

[ref43] Garcia-Simon C.; Monferrer A.; Garcia-Borras M.; Imaz I.; Maspoch D.; Costas M.; Ribas X. Size-selective encapsulation of C_60_ and C_60_-derivatives within an adaptable naphthalene-based tetragonal prismatic supramolecular nanocapsule. Chem. Commun. 2019, 55, 798–801. 10.1039/C8CC07886F. 30570641

[ref44] Sun Y.; Chen C.; Liu J.; Liu L.; Tuo W.; Zhu H.; Lu S.; Li X.; Stang P. J. Self-Assembly of Porphyrin-Based Metallacages into Octahedra. J. Am. Chem. Soc. 2020, 142, 17903–17907. 10.1021/jacs.0c08058. 32830970

[ref45] As always observed by us in the past for previous porphyrin–Ru neutral assemblies (see refs (25), (26), and (30)), the ESI-MS spectra of the model complexes and metallacycles of porphyrins showed peaks only deriving from the fragmentation (ESI mass spectra were collected in positive mode on a Perkin-Elmer APII spectrometer at 5600 eV). No molecular ion peak could be detected despite the many attempts. Similarly, elemental analysis, unless it is performed on the crystal samples (such as those used for X-ray determinations), is poorly significant for these systems due to the typical presence of crystallization molecules whose nature and number vary from batch to batch. In this work, we found repeatedly consistent elemental analysis only for model compound **1Me**. As a consequence, unambiguous characterization of the chromatographically pure products is achieved through extensive NMR, IR, and UV–vis spectroscopic investigations and, whenever possible, through the determination of X-ray structures.

[ref46] Compound **6** typically replaces the two adjacent dmso-O ligands without geometrical changes; i.e., it is the precursor of a neutral *cis*-protected metal fragment.

[ref47] As detailed in the Supporting Information, in a CDCl_3_ solution the crude product was actually an equilibrium mixture of **7** and the corresponding aqua species *t*,*c*,*c*-[RuCl_2_(CO)_2_(OH_2_)(4′MPyP)] (**7H**_**2**_**O**), suggesting that the dmso-O is easily replaced by adventitious water in the deuterated solvent.

[ref48] Bratsos I.; Alessio E. The pivotal role of Ru-dmso compounds in the discovery of well-behaved precursors. Eur. J. Inorg. Chem. 2018, 2018, 2996–3013. 10.1002/ejic.201800469.

[ref49] Like **3**, **3Me** and **4** were also obtained mainly as aqua species and could be transformed into the dmso-O species upon recrystallization. The labels **3Me** and **4** correspond to mixtures of H_2_O and dmso-O species.

[ref50] Homoleptic metallacycle **1Me** was prepared for comparative purposes. We managed to obtain its single-crystal X-ray structure, which is reported in the Supporting Information.

[ref51] Kabsch W. XDS. Acta Crystallogr., Sect. D: Biol. Crystallogr. 2010, 66, 125–132. 10.1107/S0907444909047337. 20124692 PMC2815665

[ref52] Sheldrick G. M. SHELXT - Integrated Space-Group and Crystal-Structure Determination. Acta Crystallogr., Sect. A: Found. Adv. 2015, 71, 3–8. 10.1107/S2053273314026370. 25537383 PMC4283466

[ref53] Sheldrick G. M. A Short History of SHELX. Acta Crystallogr., Sect. A: Found. Crystallogr. 2008, 64, 112–122. 10.1107/S0108767307043930. 18156677

[ref54] Emsley P.; Cowtan K. Coot: Model-Building Tools for Molecular Graphics. Acta Crystallogr., Sect. D: Biol. Crystallogr. 2004, 60, 2126–2132. 10.1107/S0907444904019158. 15572765

[ref55] Huebschle C. B.; Sheldrick G. M.; Dittrich B. ShelXle: a Qt graphical user interface for SHELXL. J. Appl. Crystallogr. 2011, 44, 1281–1284. 10.1107/S0021889811043202. 22477785 PMC3246833

[ref56] Giannozzi P.; Baroni S.; Bonini N.; Calandra M.; Car R.; Cavazzoni C.; Ceresoli D.; Chiarotti G. L.; Cococcioni M.; Dabo I.; Dal Corso A.; de Gironcoli S.; Fabris S.; Fratesi G.; Gebauer R.; Gerstmann U.; Gougoussis C.; Kokalj A.; Lazzeri M.; Martin-Samos L.; Marzari N.; Mauri F.; Mazzarello R.; Paolini S.; Pasquarello A.; Paulatto L.; Sbraccia C.; Scandolo S.; Sclauzero G.; Seitsonen A. P.; Smogunov A.; Umari P.; Wentzcovitch R. M. Quantum Espresso: a modular and open-source software project for quantum simulations of materials. J. Phys.: Condens. Matter 2009, 21, 395502 10.1088/0953-8984/21/39/395502. 21832390

[ref57] Giannozzi P.; Andreussi O.; Brumme T.; Bunau O.; Buongiorno Nardelli M.; Calandra M.; Car R.; Cavazzoni C.; Ceresoli D.; Cococcioni M.; Colonna N.; Carnimeo I.; Dal Corso A.; de Gironcoli S.; Delugas P.; DiStasio R. A.; Ferretti A.; Floris A.; Fratesi G.; Fugallo G.; Gebauer R.; Gerstmann U.; Giustino F.; Gorni T.; Jia J.; Kawamura M.; Ko H.-Y.; Kokalj A.; Küçükbenli E.; Lazzeri M.; Marsili M.; Marzari N.; Mauri F.; Nguyen N. L.; Nguyen H.-V.; Otero de la Roza A.; Paulatto L.; Poncé S.; Rocca D.; Sabatini R.; Santra B.; Schlipf M.; Seitsonen A. P.; Smogunov A.; Timrov I.; Thonhauser T.; Umari P.; Vast N.; Wu X.; Baroni S. Advanced capabilities for materials modelling with Quantum ESPRESSO. J. Phys.: Condens. Matter 2017, 29, 465901 10.1088/1361-648X/aa8f79. 29064822

[ref58] Blöchl P. E. Projector augmented-wave method. Phys. Rev. B: Condens. Matter Mater. Phys. 1994, 50, 17953–17979. 10.1103/PhysRevB.50.17953. 9976227

[ref59] Perdew J. P.; Burke K.; Ernzerhof M. Generalized gradient approximation made simple. Phys. Rev. Lett. 1996, 77, 3865–3868. 10.1103/PhysRevLett.77.3865. 10062328

[ref60] Makov G.; Payne M. C. Periodic boundary conditions in ab initio calculations. Phys. Rev. B: Condens. Matter Mater. Phys. 1995, 51, 4014–4022. 10.1103/PhysRevB.51.4014. 9979237

