# Supplementary material for: A Flexible Synthetic Strategy for the Preparation of Heteroleptic Metallacycles of Porphyrins
Source: Inorg Chem. 2021 Jul 15;60(15):11503–13. doi: 10.1021/acs.inorgchem.1c01511 (PMC8389808; doi:10.1021/acs.inorgchem.1c01511)
Supplement: Supplementary file 1 — ic1c01511_si_001.pdf [file ic1c01511_si_001.pdf]

A flexible synthetic strategy for the preparation of heteroleptic metallacycles of porphyrins.

Alessio Vidal,[a] Federica Battistin,[a,†] Gabriele Balducci,[a] Elisabetta Iengo,[a] Enzo Alessio[a]*

[a] Department of Chemical and Pharmaceutical Sciences, University of Trieste, Via L. Giorgieri 1, 34127 Trieste, Italy.

[†]Current address: Department of Chemistry, University of Zurich, Winterthurerstrasse 190, CH-8057 Zurich, Switzerland.

Supporting Information

Model systems with 4′MPyP

Synthesis of [*t,c,c*-$RuCl_2(CO)_2$(dmso-O)(4′MPyP)] (7)

Treatment of 4′MPyP with an excess of *t,c,c*-[$RuCl_2(CO)_2$(dmso-O)$_2$] (**6**) (4 equiv.) in chloroform at room temperature smoothly afforded the intermediate *t,c,c*-[$RuCl_2(CO)_2$(dmso-O)(4′MPyP)] (**7**) in excellent yield (Scheme S1). After solvent removal, the dark purple solid was washed with MeOH to remove the unreacted ruthenium complex and the released DMSO.

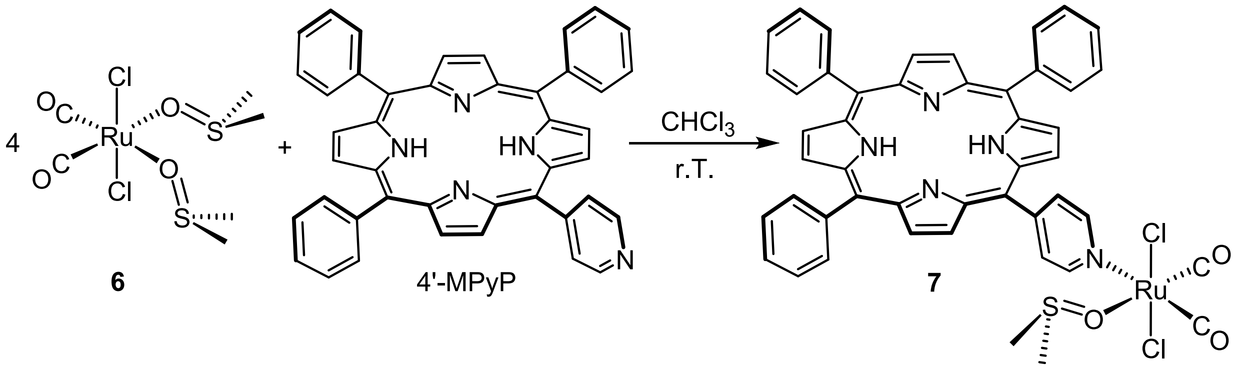

Scheme S1. Synthesis of *t,c,c*-[$RuCl_2(CO)_2$(dmso-O)(4′MPyP)] (**7**).

The ^{1}H NMR spectrum of the crude reaction product in $CDCl_3$ (Figures S1 and S2) presents two sets of partially overlapped resonances, with the exception of the signals of the pyridyl ring (H2,6 and H3,5) which are shifted to higher frequencies compared to the free ligand, consistent with 4′MPyP being coordinated to the {*trans,cis*-$RuCl_2(CO)_2$(L)} fragment (**7**, L = dmso-O; **7aq**, L = H_2O).

We suggest that the set of signals at lower frequencies belongs to **7** and the other to **7aq** in which the dmso molecule is replaced by a molecule of water of the deuterated solvent. In fact, the resonance of dmso-O (a singlet at 2.94 ppm) is in 3:1 ratio with the resonances of the H2,6 and H3,5 pyridyl protons that fall at 9.45 and 8.36 ppm, respectively. In agreement with this hypothesis, *i*) upon addition of DMSO the signals of **7** increased in intensity at the expenses of those of **7aq**; *ii)* the ^{1}H NMR spectrum in DMSO-d_6 presents only one set of signals, consistent with the equilibrium between **7aq** and **7** being completely shifted towards **7**. The singlet for the dmso-O at 2.97 ppm decreases with time due to exchange with the deuterated DMSO.

Synthesis of [{*t,c,c*-$RuCl_2(CO)_2$(4′MPyP)}$_2$(4,4′-bpy)].

Treatment of **7** with 0.5 eq of the linear ditopic ligand 4,4′-bipyridine (4,4′-bpy) in $CHCl_3$ at room temperature afforded to [{*t,c,c*-$RuCl_2(CO)_2$(4′MPyP)}$_2$(4,4′-bpy)] (Scheme S2).

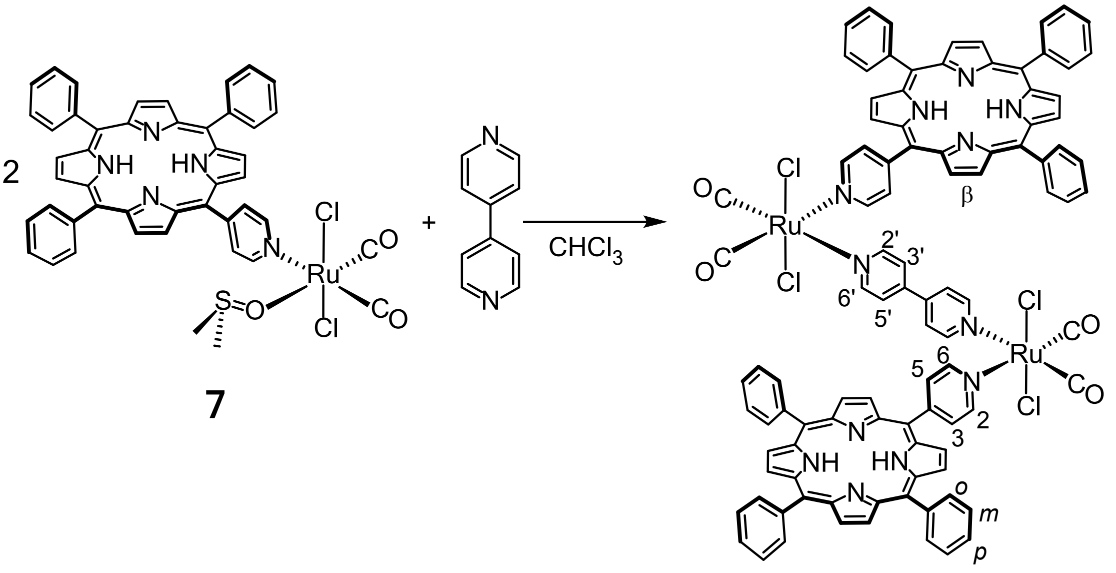

Scheme S2: Synthesis of [{*t,c,c*-$RuCl_2(CO)_2$(4′MPyP)}$_2$(4,4′-bpy)].

The ^{1}H NMR spectrum of the crude reaction product in $CDCl_3$ (Figure S4) shows two downfield-shifted resonances for 4,4′-bpy (consistent with 4,4′-bpy being symmetrically coordinated to ruthenium) and one set of resonances for the two equivalent porphyrins. Assignments were done through a COSY spectrum (Figure S5). Overall, the spectrum, including integration, is in agreement with the structure of [{*t,c,c*-$RuCl_2(CO)_2$(4′MPyP)}$_2$(4,4′-bpy)]. The resonances of the phenyl protons of the porphyrins are splitted in two multiplets in 1:2 ratio: This pattern is consistent with a free rotation around the Ru–N bonds: the phenyl rings *cis* to the coordinated pyridyl ring (positions 10 and 20) fall into the shielding cone of the adjacent 4,4′-bpy so their signals get shifted to lower frequencies compared to those of the other phenyl ring (position 15).[S1,S2]

The IR spectrum presented two stretching bands for the CO respectively at 2074 and 2015 cm^{-1}, in agreement with two *cis* carbonyls.

Synthesis of [{*t,c,c*-$RuCl_2(CO)_2$(4′MPyP)}$_2$(4′*trans*DPyP)]

The three-porphyrin adduct [{*t,c,c*-$RuCl_2(CO)_2$(4′MPyP)}$_2$(4′*trans*DPyP)] was obtained by treatment of **7** with ca. 0.5 equiv. of 4′*trans*DPyP, another linear ditopic ligand. The reaction was performed on a small scale in $CDCl_3$, in an NMR tube (Scheme S3).

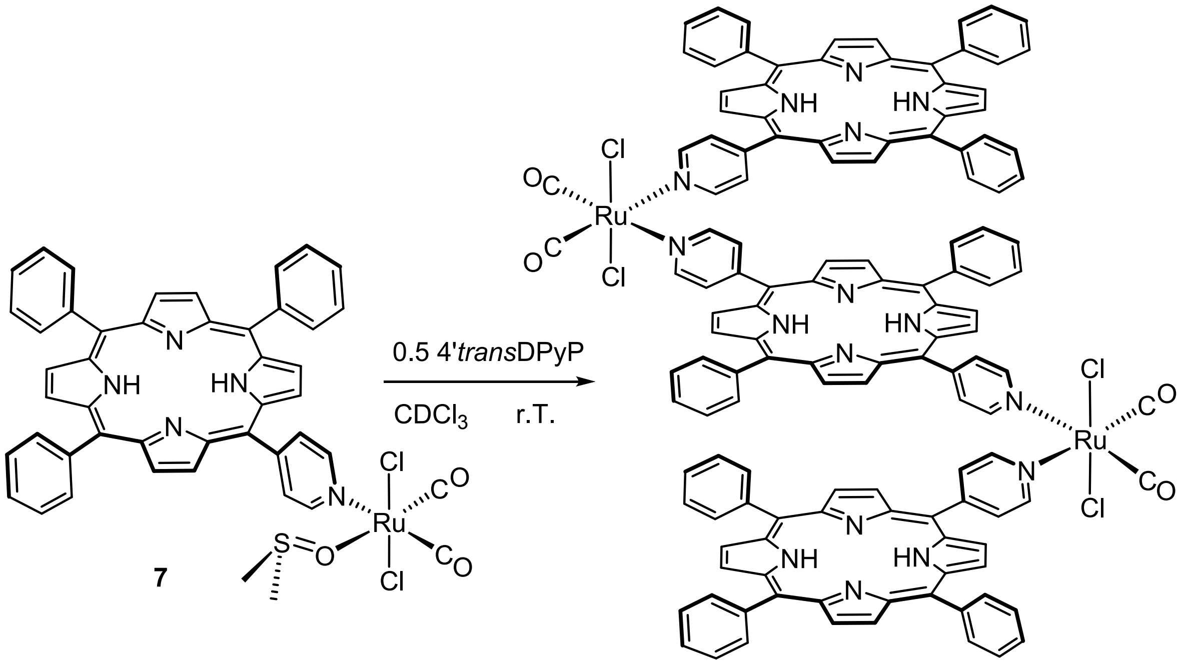

Scheme S3. Synthesis of [{*t,c,c*-$RuCl_2(CO)_2$(4′MPyP)}$_2$(4′*trans*DPyP)].

In the ^{1}H NMR spectrum of the product (Figure S6) most protons of the two different porphyrins give partially overlapping resonances, with the notable exception of the NH protons that give two well-resolved singlets at –2.81 ppm (4H, 4′MPyP) and –2.87 ppm (2H, 4′*trans*DPyP), whose intensity is consistent with the 1:2 stoichiometry of the product. The resonance of 4′*trans*DPyP falls at slightly lower frequencies since its NH protons fall into the shielding cone of the two equivalent 4′MPyPs. The resonances of the *ortho* and *meta+para* protons of the eight phenyl rings are splitted into three well resolved multiplets in 1:2:1 ratio, which are pairwise connected in the COSY spectrum (Figure S7). Due to mutual shielding in each group the multiplet at the lowest frequency belongs to the protons of 4′*trans*DPyP, which fall into the shielding cone of the adjacent 4′MPyPs, whereas the multiplet at highest frequency belongs to the protons of the phenyl ring in position 15 on the two 4′MPyPs.

Experimental section

Synthesis of the complexes

***t,c,c*-[$RuCl_2(CO)_2$(dmso-O)(4′MPyP)] (7).** A 40.0 mg amount of *t,c,c*-[$RuCl_2(CO)_2$(dmso-O)$_2$] (**6**) (0.10 mmol) was dissolved in 10 mL of chloroform. Addition to the yellow solution of 16.0 mg of 4′MPyP (0.025 mmol, Ru/MPyP 4:1 ratio) afforded a purple solution that was stirred at room temperature for 5h. The solvent was removed by rotary evaporation and the purple powder was washed with methanol (suspended in 4 mL of MeOH and centrifuged for 10 min, × 3). The resulting purple powder was dried *in vacuo*. (Yield: 18 mg, 78%). ^{1}H NMR ($CDCl_3$), δ (ppm) (as said above, in this solvent the complex releases part of the dmso-O, probably replaced by

adventitious water): 9.67 (d, 2H, H2,6 **7aq**), 9.45 (d, 2H, H2,6 **7**), 8.90 (m, 16H, Hβ **7** + **7aq**), 8.63 (d, 2H, H3,5 **7aq**), 8.36 (d, 2H, H3,5 **7**), 8.23 (m, 12H, H*o* **7** + **7aq**), 7.76 (m, 18H, H*m*+*p* **7** + **7aq**), 2.94 (s, 6H, C*H*$_3$ **7**), –2.66 (br s, 4H, NH **7** + **7aq**). (DMSO-*d*$_6$), δ (ppm): 9.35 (d, 2H, H2,6 **7**), 8.86 (m, 8H, Hβ **7**), 8.62 (d, 2H, H3,5 **7**), 8.21 (d, 6H, H*o* **7**), 7.85 (m, 9H, H*m*+*p* **7**), 2.97 (s, 6H, C*H*$_3$ dmso-O **7**), –2.95 (br s, 2H, NH **7**). ^{13}C NMR (CDCl$_3$ + 2 μL DMSO-*d*$_6$), δ (ppm): 195.80 (*C*O), 194.18 (*C*O), 150.54 (C2,6), 141.80 (Cβ), 134.59 (C*o*), 130.57 (C3,5), 127.83 (C*m*+*p*), 39.40 (*C*H$_3$). Selected IR absorption (chloroform solution, cm^{-1}): 2077 (ν_{CO}), 2016 (ν_{CO}).

[*t,c,c*-{RuCl$_2$(CO)$_2$(4′MPyP)}$_2$(4,4′-bpy)]. A 10.0 mg amount of *t,c,c*-[RuCl$_2$(CO)$_2$(dmso-O)(4′MPyP)] (**7**) (0.010 mmol) was dissolved in 2 mL of chloroform. After addition of 0.8 mg of 4,4′-bpy (0.005 mmol, Ru/4,4′-bpy = 2) the purple solution was stirred in the dark. The reaction was monitored by TLC (silica gel, CHCl$_3$). After 48h, when the spot corresponding to **7** (Rf = 0.32) was no longer visible, the solvent was removed by rotary evaporation. The purple solid was cleaned through a small silica gel column (eluted with chloroform, Rf = 0.88). The yield of pure [*t,c,c*-{RuCl$_2$(CO)$_2$(4′MPyP)}$_2$(4,4′-bpy)] was not measured, but it was sufficient for NMR and IR analysis. ^{1}H NMR (CDCl$_3$), δ (ppm): 9.42 (d, 4H, H2,6 bpy), 9.34 (d, 4H, H2,6 4′MPyP), 8.82 (m, 16H, Hβ), 8.29 (d, 4H, H3,5 4′MPyP), 8.15 (m, 12H, H*o*), 7.88 (d, 4H, H3,5 bpy), 7.71 (m, 18H, H*m*+*p*), –2.83 (br s, 4H, NH). ^{13}C NMR from the HSQC spectrum (CDCl$_3$), δ (ppm): 153.9 (C2,6 4′MPyP), 150.6 (C2,6 bpy), 134.5 (C*o*), 131.0 (C3,5 4′MPyP), 127.3 (C*m*+*p*), 123.1 (C3,5 bpy). Selected IR absorption bands (chloroform solution, cm^{-1}): 2074 (ν_{CO}), 2015 (ν_{CO}).

[{*t,c,c*-RuCl$_2$(CO)$_2$(4′MPyP)}$_2$(4′*trans*DPyP)]. The explorative reaction was performed on a small scale in an NMR tube only. A 4.5 mg amount of *t,c,c*-[RuCl$_2$(CO)$_2$(dmso-O)(4′MPyP)] (**7**) (0.0049 mmol) was dissolved in 0.6 mL of CDCl$_3$ in an NMR tube. After addition of 1.5 mg of 4′*trans*DPyP (0.0024 mmol, Ru/4′*trans*DPyP = 2) the purple solution was stored in the dark. The reaction was monitored by TLC (silica gel, CHCl$_3$) and ^{1}H NMR spectroscopy after 5, 24 and 48h. According to the TLC and NMR analysis, after 48h there was no residual unreacted 4′*trans*DPyP (Rf = 0.08). The mixture was run through a small silica gel column eluted with a CHCl$_3$/*n*-hexane mixture (99/1), collecting three fractions. According to TLC and ^{1}H NMR analysis, the first fraction contained the know compound *t,c,c*-[RuCl$_2$(CO)$_2$(4′MPyP)$_2$] (TLC Rf = 0.92, chloroform), which indicate the occurrence of partial scrambling of the 4′MPyP ligand. The second band (TLC Rf = 0.83, chloroform) contained the desired product [{*t,c,c*-RuCl$_2$(CO)$_2$(4′MPyP)}$_2$(4′*trans*DPyP)] (with a minor amount of *t,c,c*-[RuCl$_2$(CO)$_2$(4′-MPyP)$_2$]). The yield was not determined. ^{1}H NMR (CDCl$_3$), δ (ppm): 9.58 (m, 8H, H2,6), 8.86 (m, 24H, Hβ), 8.43 (m, 8H, H3,5), 8.14 (m, 16H, H*o*), 7.68 (m, 24H, H*m*+*p*), –2.81 (br s, 4H, NH 4′MPyP), –2.87 (br s, 2H, NH 4′*trans*DPyP). Selected IR absorption bands (chloroform solution, cm^{-1}): 2076 (ν_{CO}), 2015 (ν_{CO}).

References

S1) Alessio, E.; Macchi, M.; Heath, S. L.; Marzilli, L. G. Ordered supramolecular porphyrin arrays from a building block approach utilizing pyridylporphyrins and peripheral ruthenium complexes and identification of a new type of mixed-metal building block *Inorg. Chem.* **1997**, *36*, 5614-5623.

S2) Vidal, A.; Battistin, F.; Balducci, G.; Demitri, N.; Iengo, E.; Alessio E. The rare example of stereoisomeric 2+2 metallacycles of porphyrins featuring chiral-at-metal octahedral ruthenium corners *Inorg. Chem.* **2019**, *58*, 7357-7367.

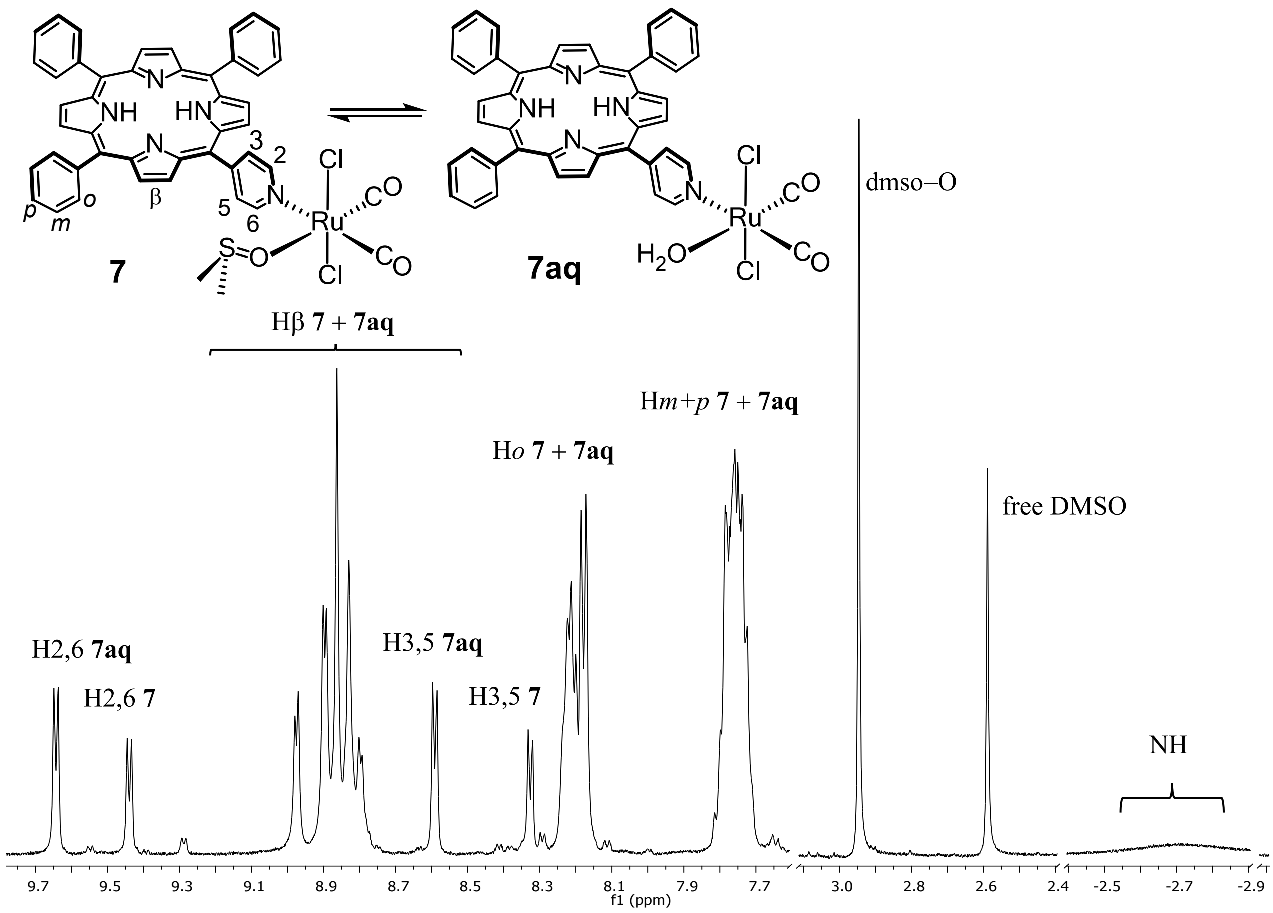

Figure S1. ^{1}H NMR spectrum in $CDCl_3$ of an equilibrium mixture of *t,c,c*-[$RuCl_2(CO)_2$(dmso-O)(4′MPyP)] (**7**) and *t,c,c*-[$RuCl_2(CO)_2(OH_2)$(4′MPyP)] (**7H$_2$O**), crude of the reaction between the Ru precursor **6** and an excess of 4′MPyP.

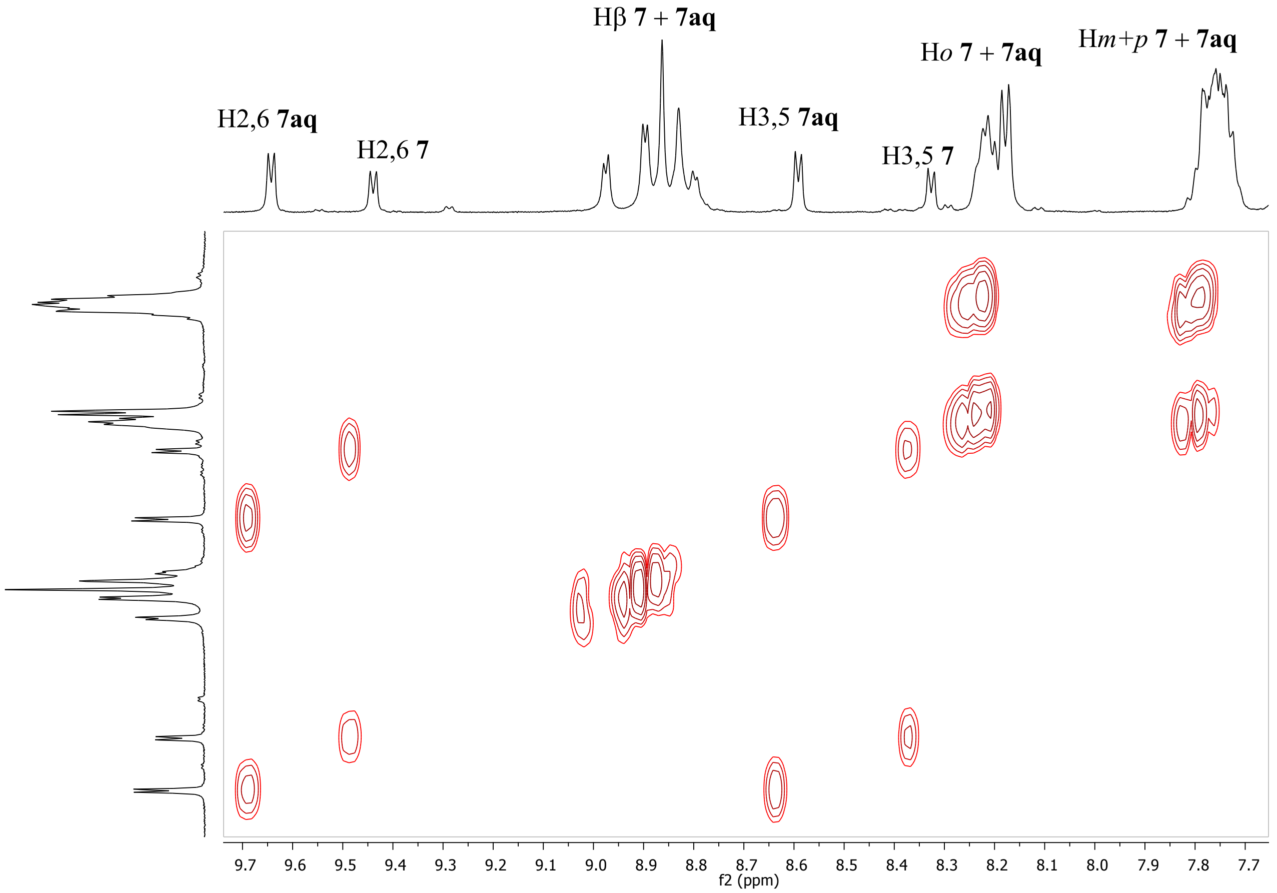

Figure S2. ^{1}H-^{1}H COSY spectrum in $CDCl_3$ of the equilibrium mixture of *t,c,c*-[$RuCl_2(CO)_2$(dmso-O)(4′MPyP)] (**7**) and *t,c,c*-[$RuCl_2(CO)_2(OH_2)$(4′MPyP)] (**7H$_2$O**) of Figure S1. See Fig. S1 for labelling scheme.

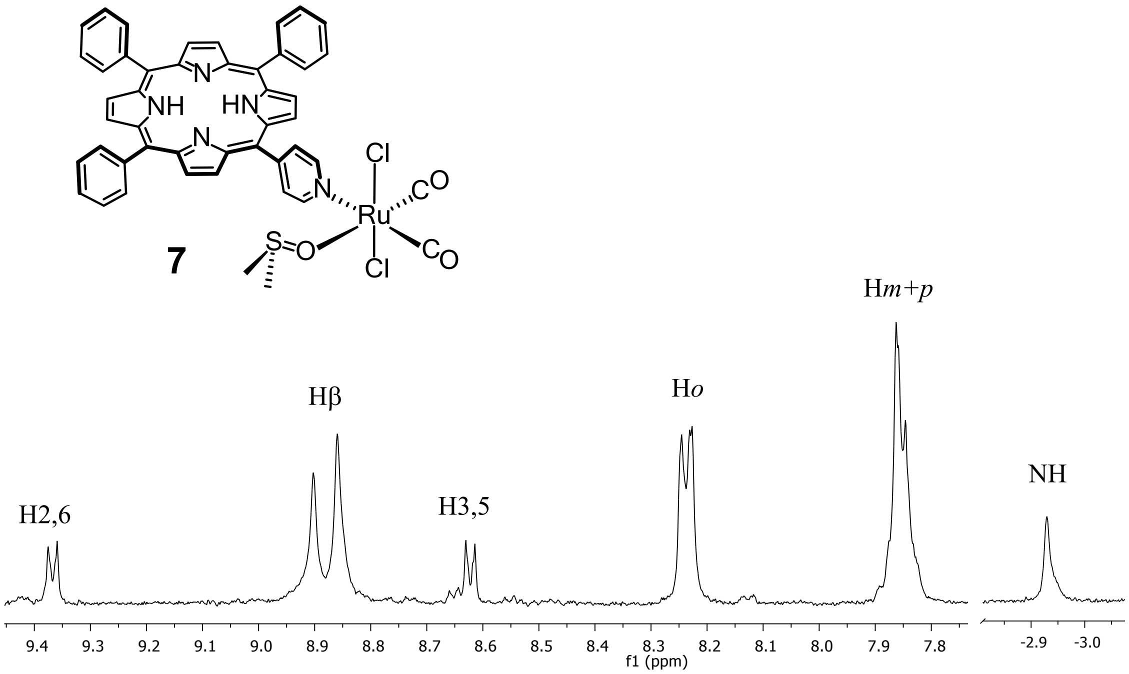

Figure S3. ^{1}H NMR spectrum in DMSO-d_6 of the crude of the reaction between the Ru precursor **6** and an excess of 4′MPyP. See Fig. S1 for labelling scheme.

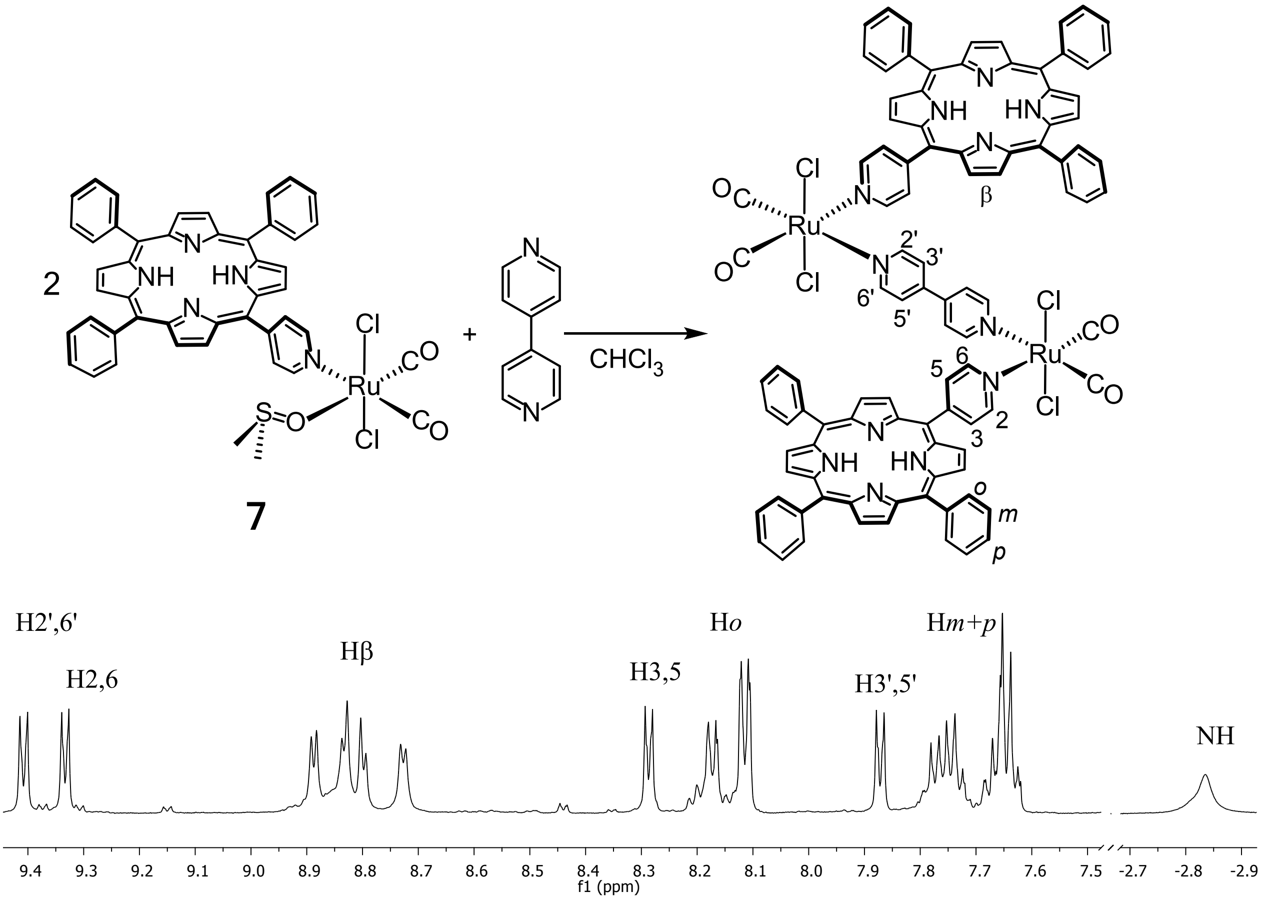

Figure S4. ^{1}H NMR spectrum in $CDCl_3$ of [{*t,c,c*-$RuCl_2(CO)_2$(4′MPyP)}$_2$(4,4′-bpy)] with labelling scheme.

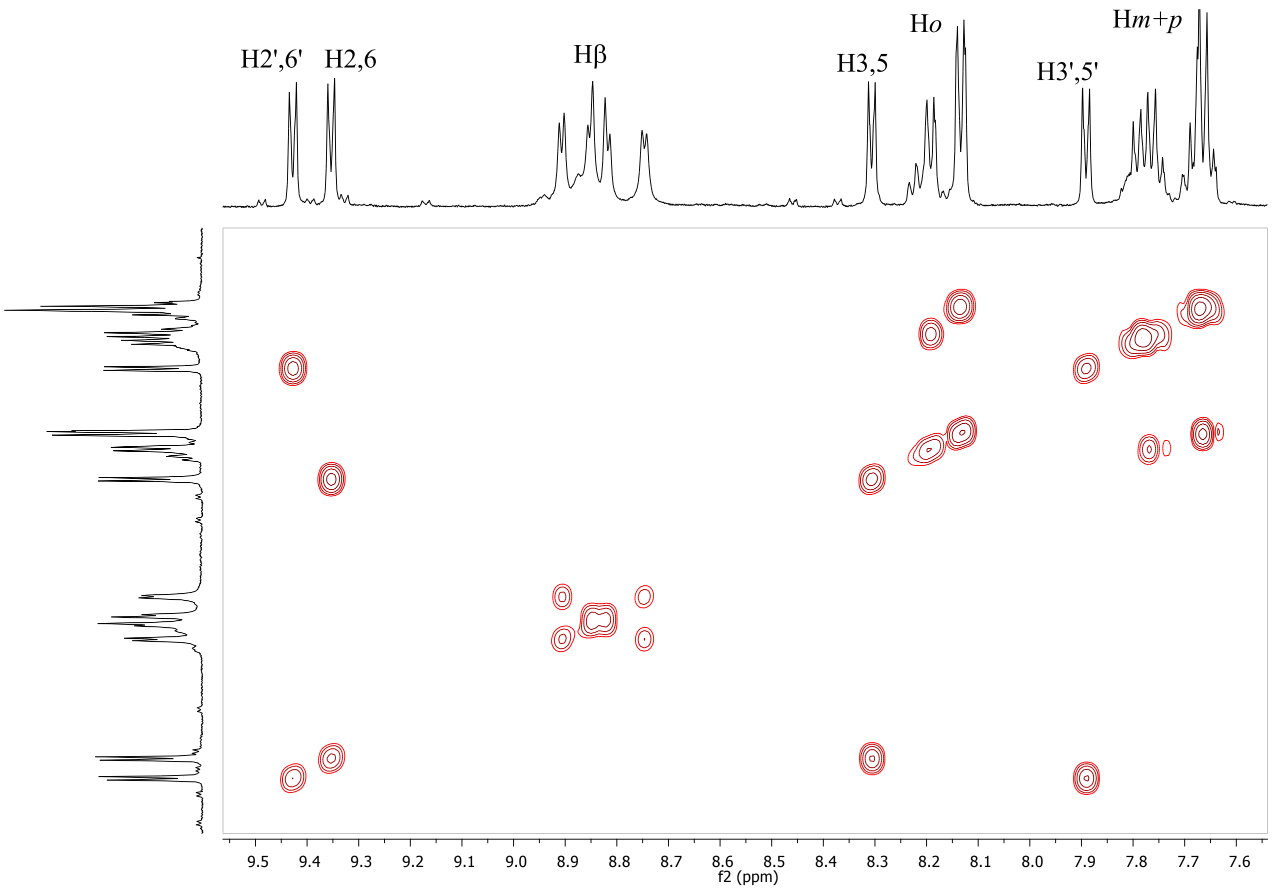

Figure S5. ^{1}H-^{1}H COSY spectrum (aromatic region) in $CDCl_3$ of [{*t,c,c*-$RuCl_2(CO)_2$(4′MPyP)}$_2$(4,4′-bpy)]; see Figure S4 for the labelling scheme.

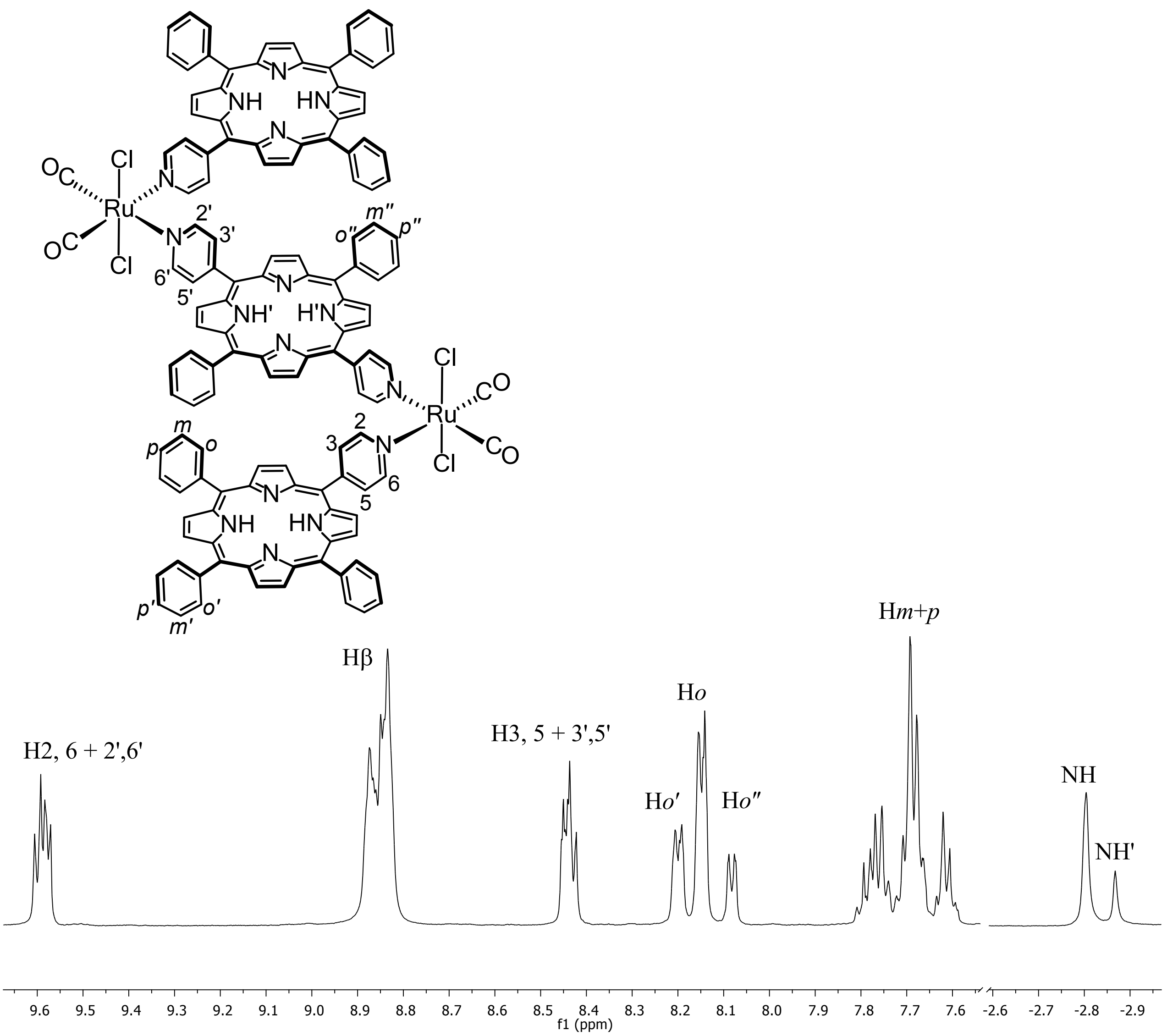

Figure S6. ^{1}H NMR spectrum in $CDCl_3$ of [{*t,c,c*-$RuCl_2(CO)_2$(4′MPyP)}$_2$(4′*trans*DPyP)] with labelling scheme.

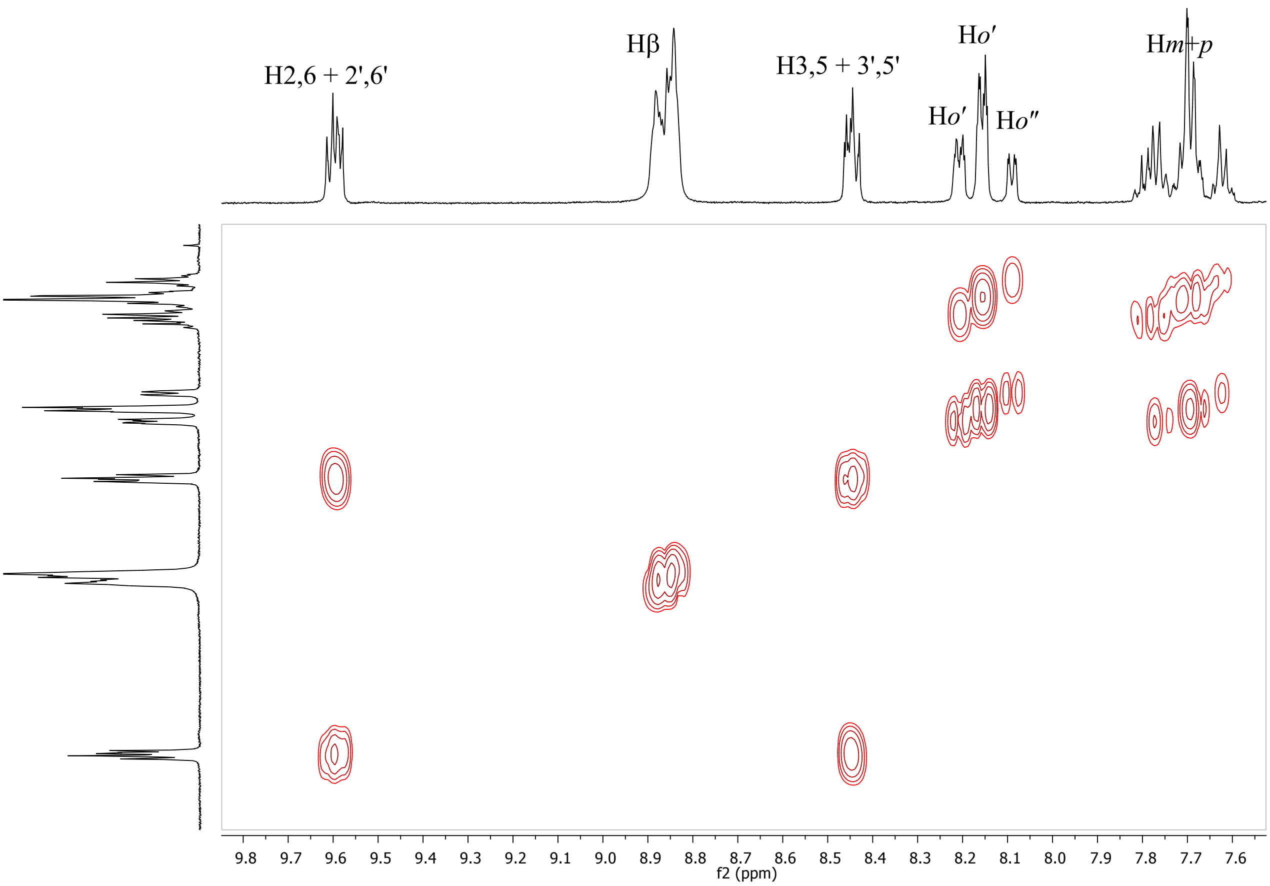

Figure S7. ^{1}H-^{1}H COSY spectrum (aromatic region) in $CDCl_3$ of [{*t,c,c*-$RuCl_2(CO)_2$(4′MPyP)}$_2$(4′*trans*DPyP)]; see Figure S6 for the labelling scheme.

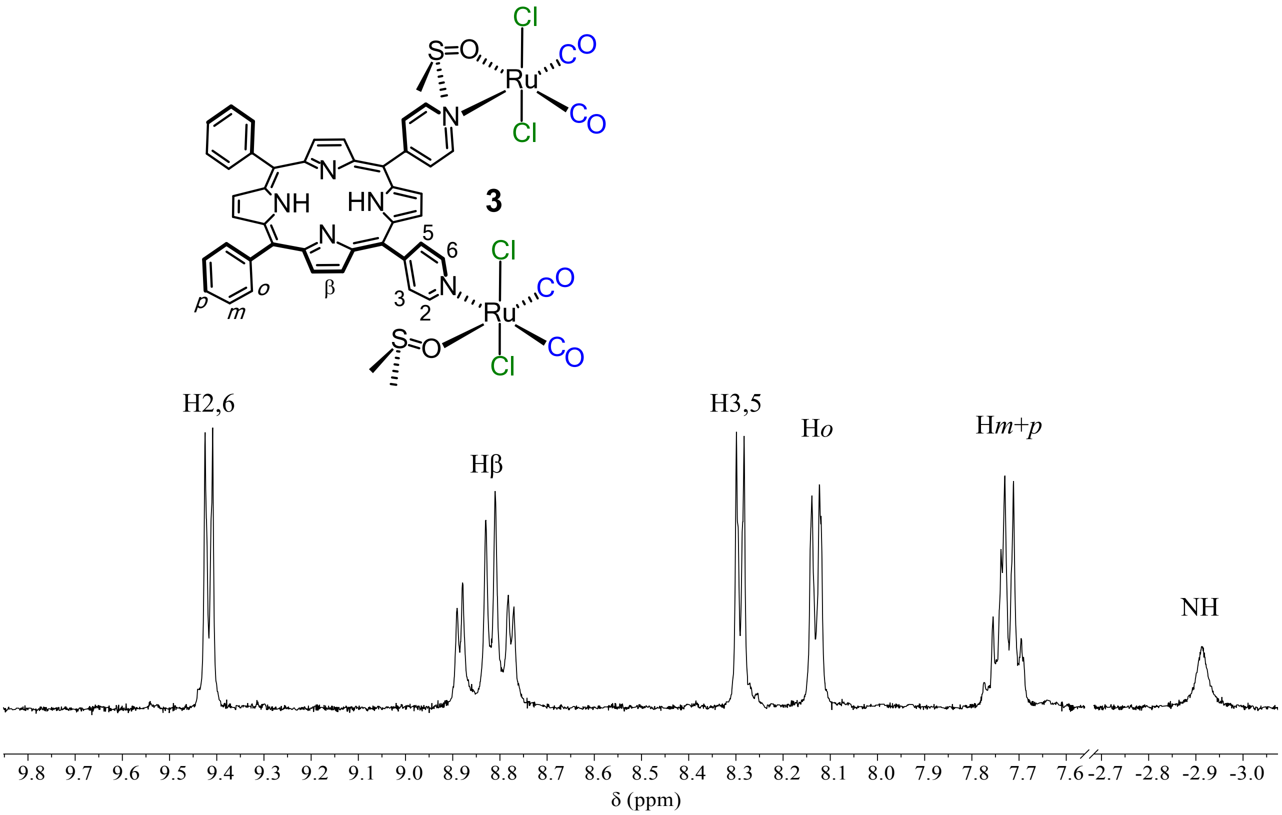

Figure S8. ^{1}H NMR spectrum of crude **3**, i.e. a mixture of [{*t*,*c*,*c*-$RuCl_2(CO)_2(OH_2)$}$_2$(4′*cis*DPyP)] (**4H$_2$O**) and [{*t*,*c*,*c*-$RuCl_2(CO)_2$(dmso-O)}$_2$(4′*cis*DPyP)] (**3**), in DMSO-d_6 with labeling scheme. In this solvent the coordinated water in **3H$_2$O** is replaced by DMSO yielding pure **3**.

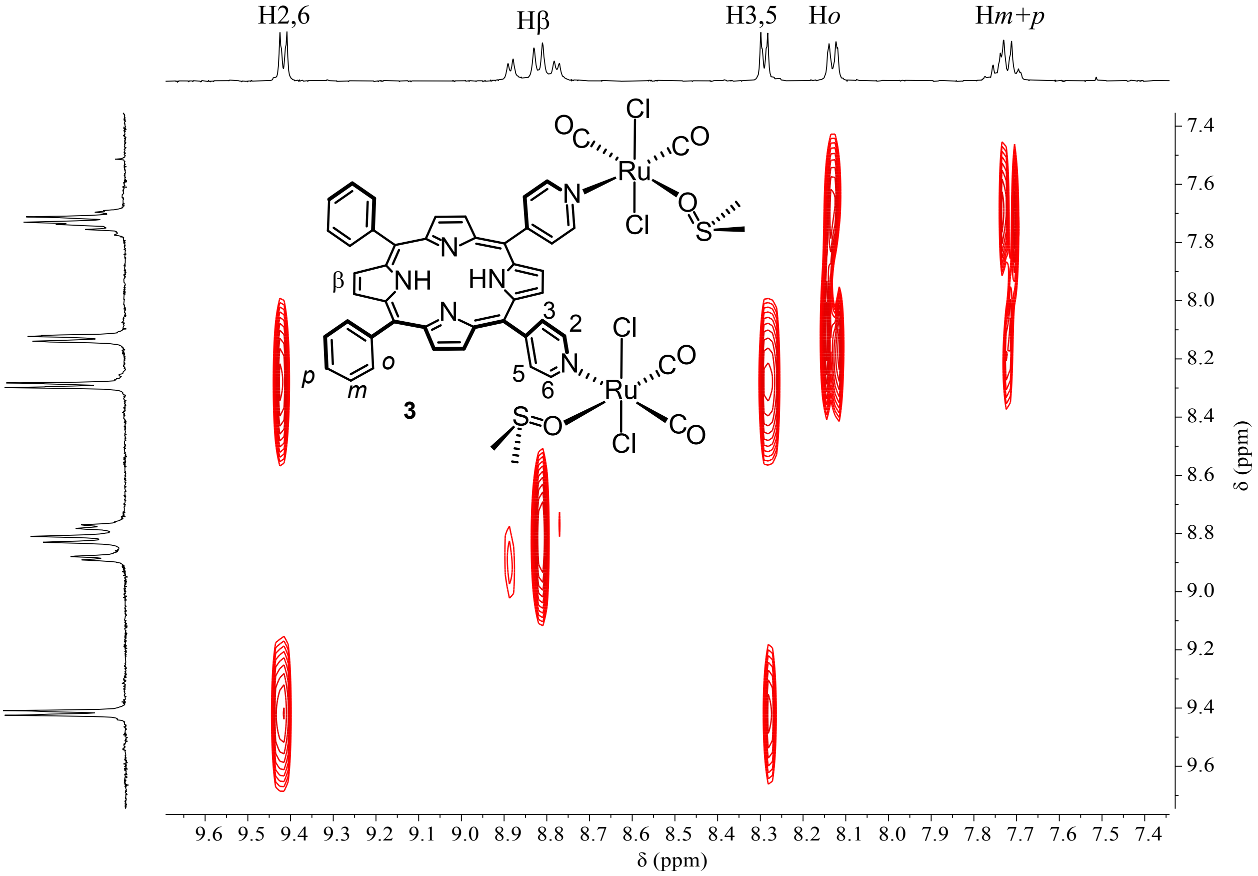

Figure S9. ^{1}H-^{1}H COSY spectrum (aromatic region) in DMSO-d_6 of [{*t*,*c*,*c*-$RuCl_2(CO)_2$(dmso-O)}$_2$(4′*cis*DPyP)] (**3**).

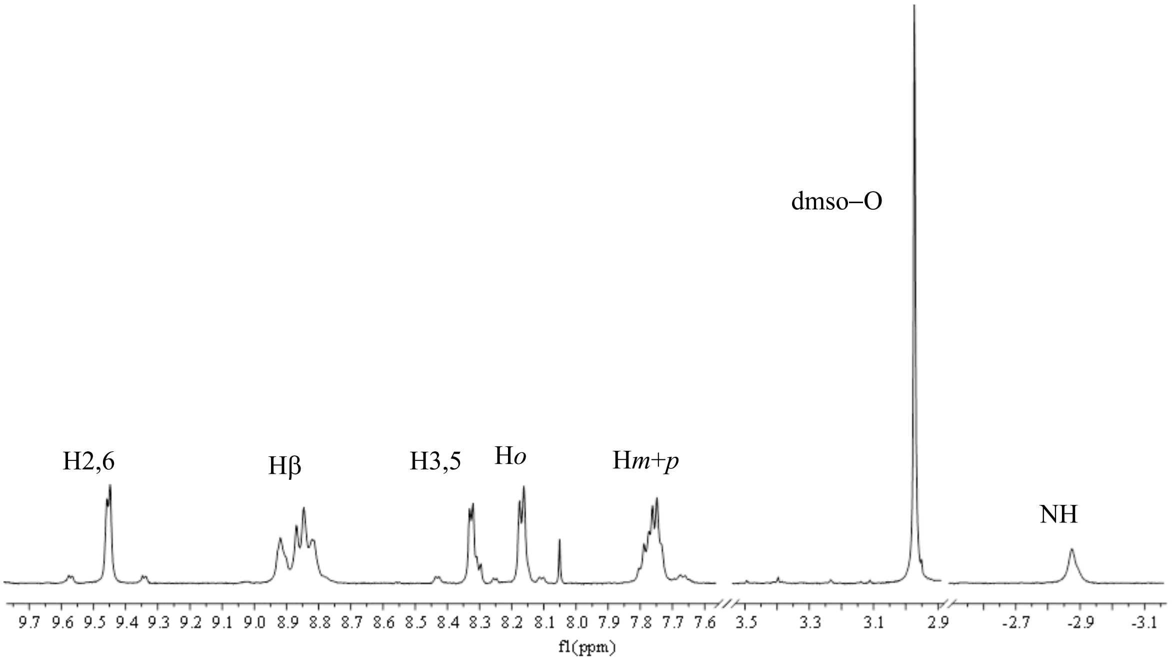

Figure S10. ^{1}H NMR spectrum in $CDCl_3$ of recrystallized [{*t*,*c*,*c*-$RuCl_2(CO)_2$(dmso-O)}$_2$(4′*cis*DPyP)] (**3**). See Figure S8 for labelling scheme.

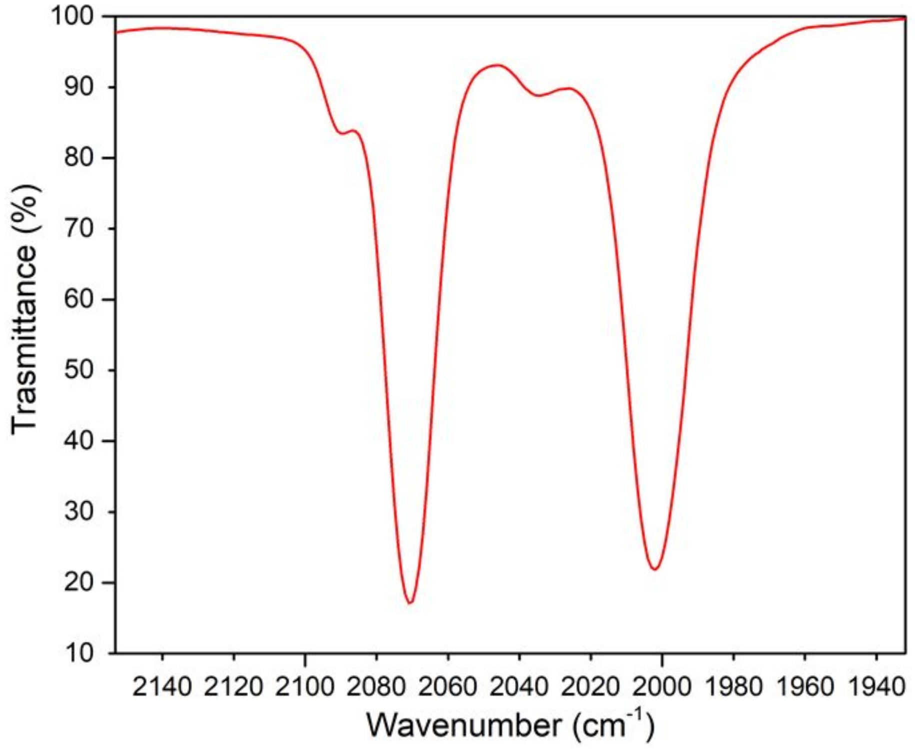

Figure S11. CO stretching region of the IR spectrum in chloroform solution of [{*t*,*c*,*c*-$RuCl_2(CO)_2$(dmso-O)}$_2$(4′*cis*DPyP)] (**3**).

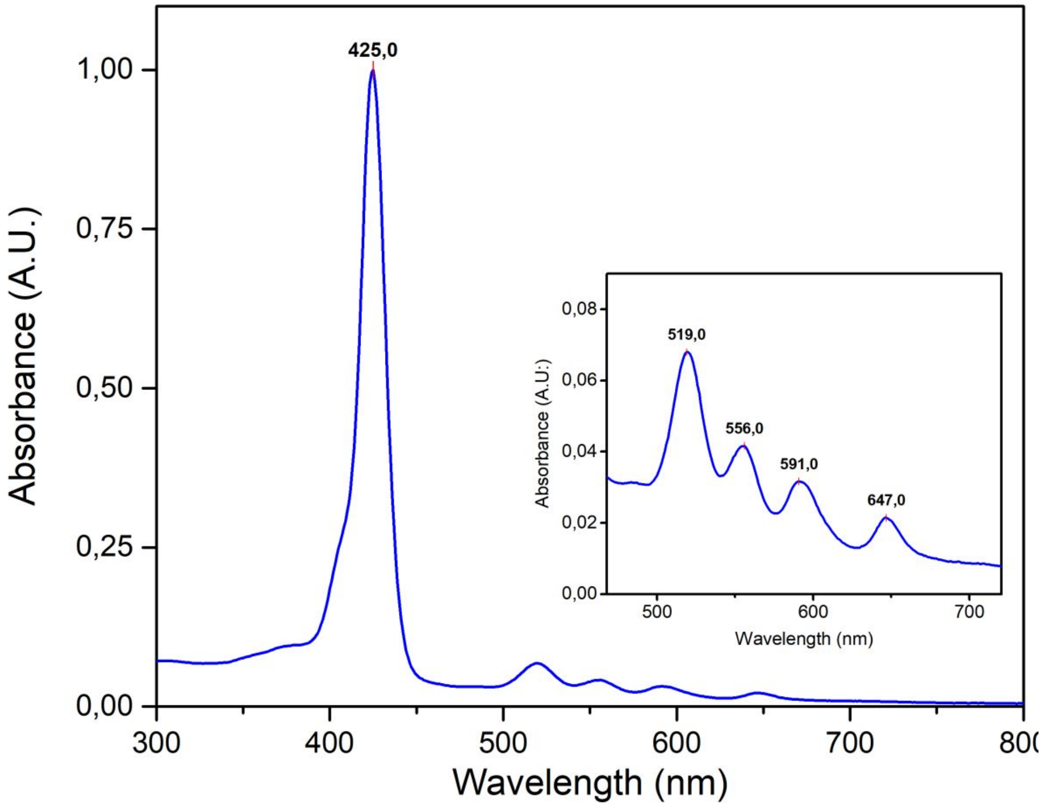

Figure S12. Normalized UV-vis absorption spectrum ($CHCl_3$) of [{*t*,*c*,*c*-$RuCl_2(CO)_2$(dmso-O)}$_2$(4′*cis*DPyP)] (**3**).

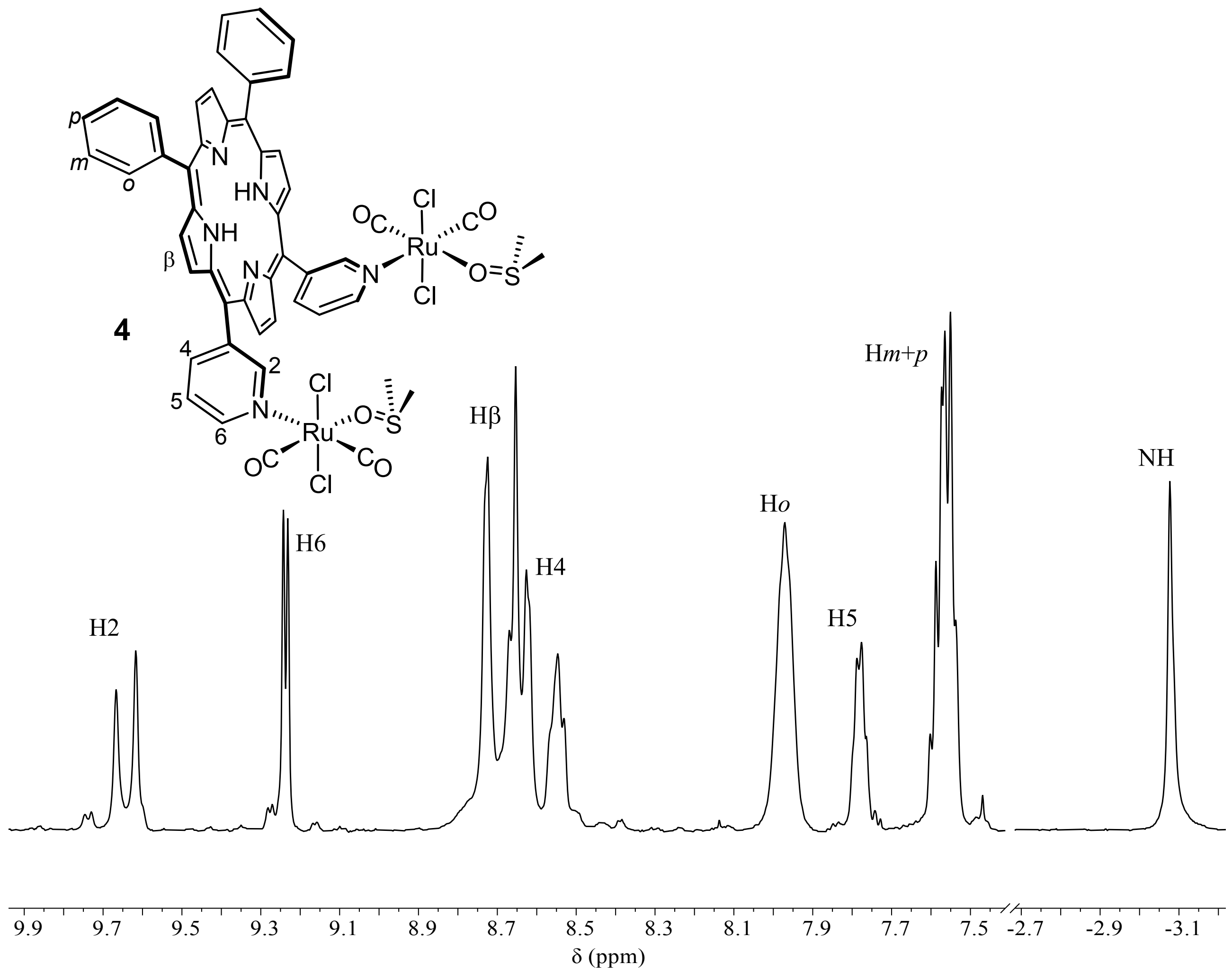

Figure S13. ^{1}H-NMR spectrum of [{*t,c,c*-$RuCl_2(CO)_2$(dmso-O)}$_2$(3′*cis*DPyP)] (**4**) in $CDCl_3$ + DMSO-d_6 with labelling scheme.

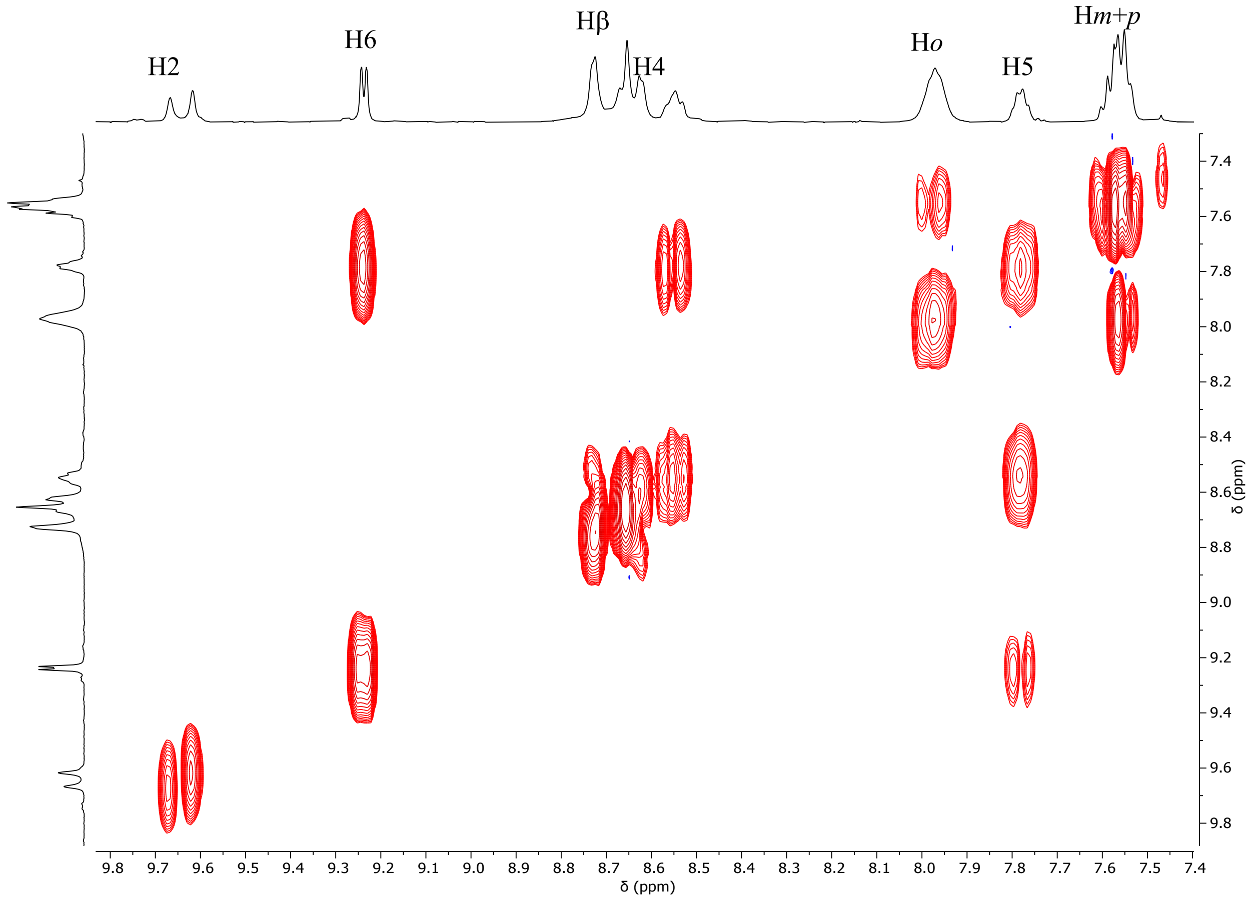

Figure S14. ^{1}H-^{1}H COSY spectrum (aromatic region) in $CDCl_3$ + DMSO-d_6 of [{*t*,*c*,*c*-$RuCl_2(CO)_2$(dmso-O)}$_2$(3′*cis*DPyP)] (**4**). See Fig. S13 for labelling scheme.

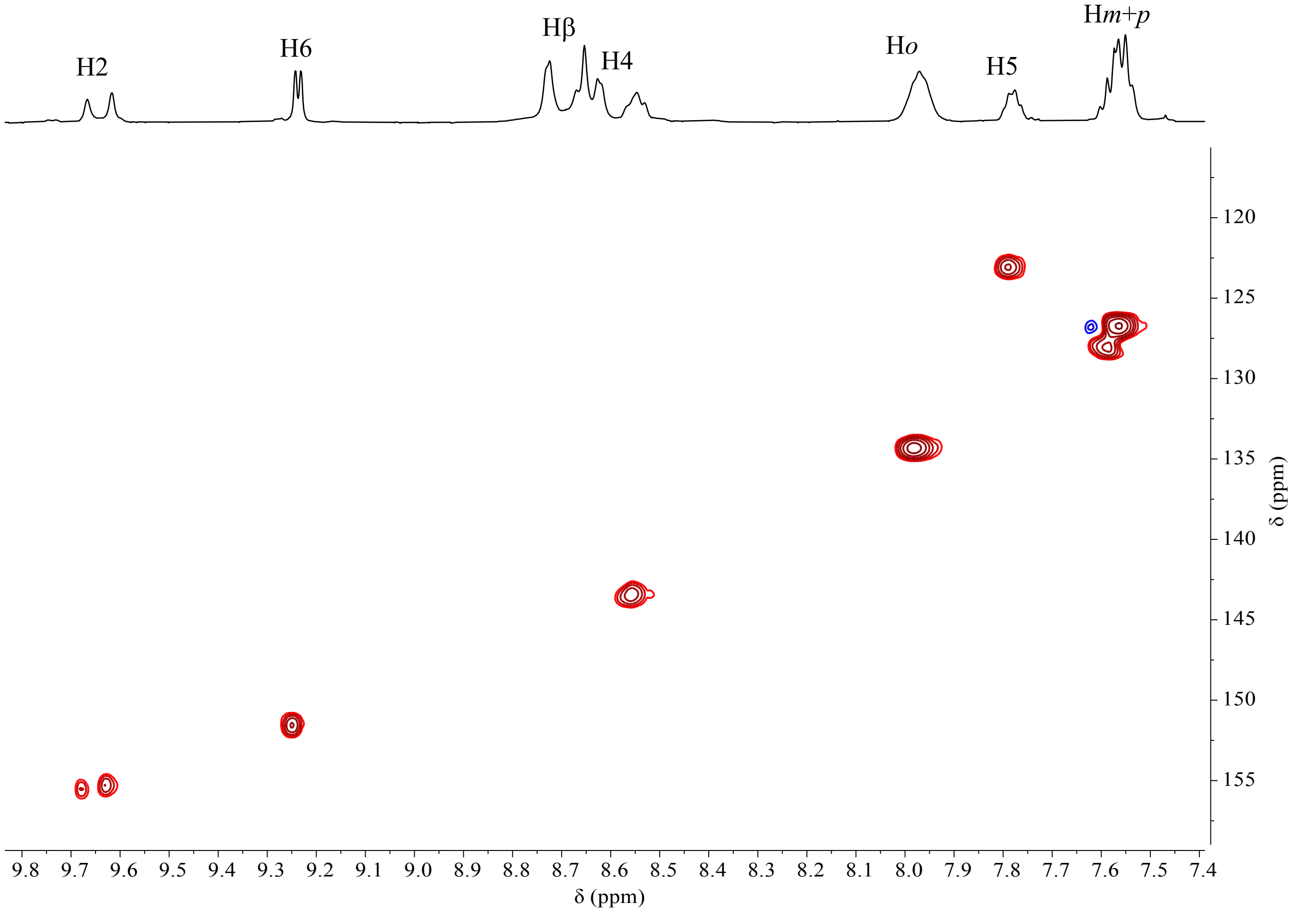

Figure S15. ^{1}H-^{13}C HSQC spectrum (aromatic region) in $CDCl_3$ + DMSO-d_6 of [{*t*,*c*,*c*-$RuCl_2(CO)_2$(dmso-O)}$_2$(3′*cis*DPyP)] (**4**).

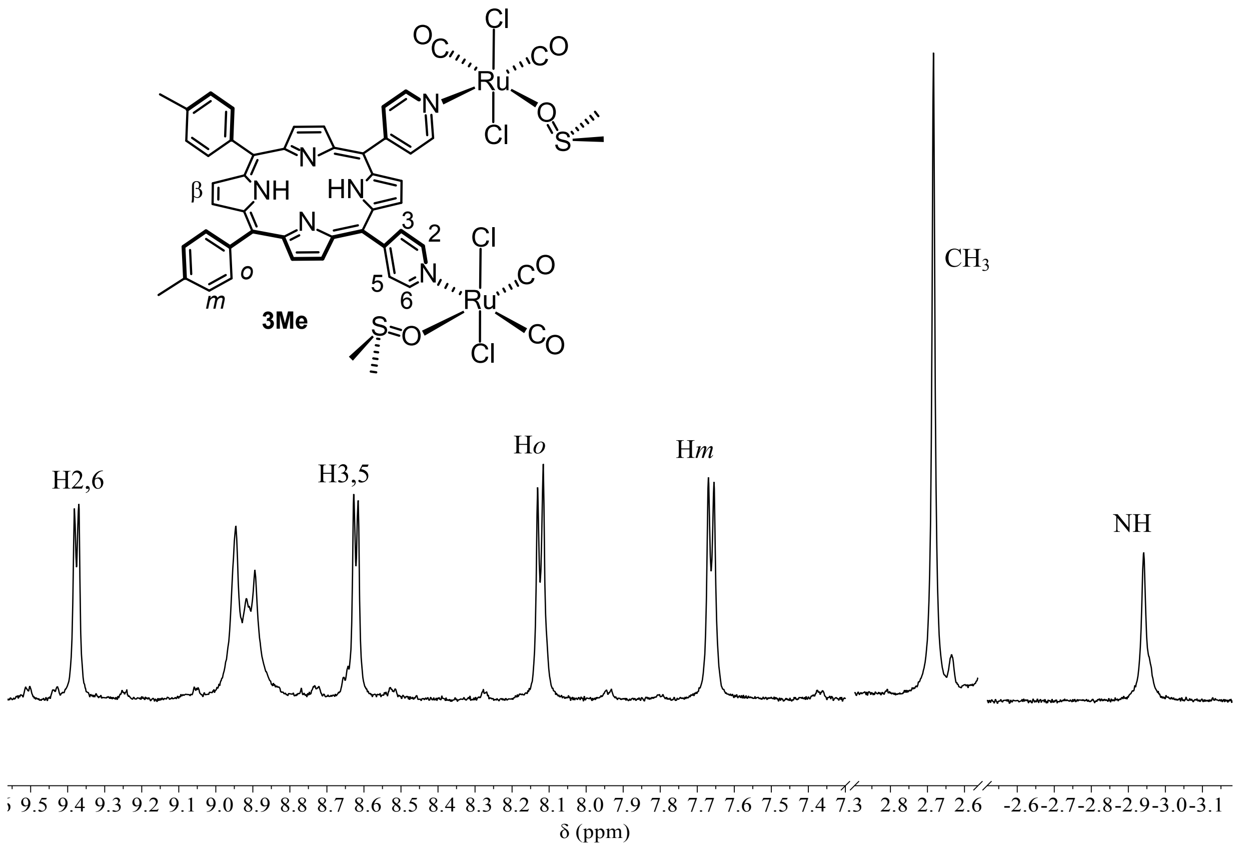

Figure S16. ^{1}H-NMR spectrum of [{*t,c,c*-RuCl$_2$(CO)$_2$(dmso-O)}$_2$(4′*cis*DPyMP)] (**3Me**) in CDCl$_3$ + DMSO-d_6 with labelling scheme.

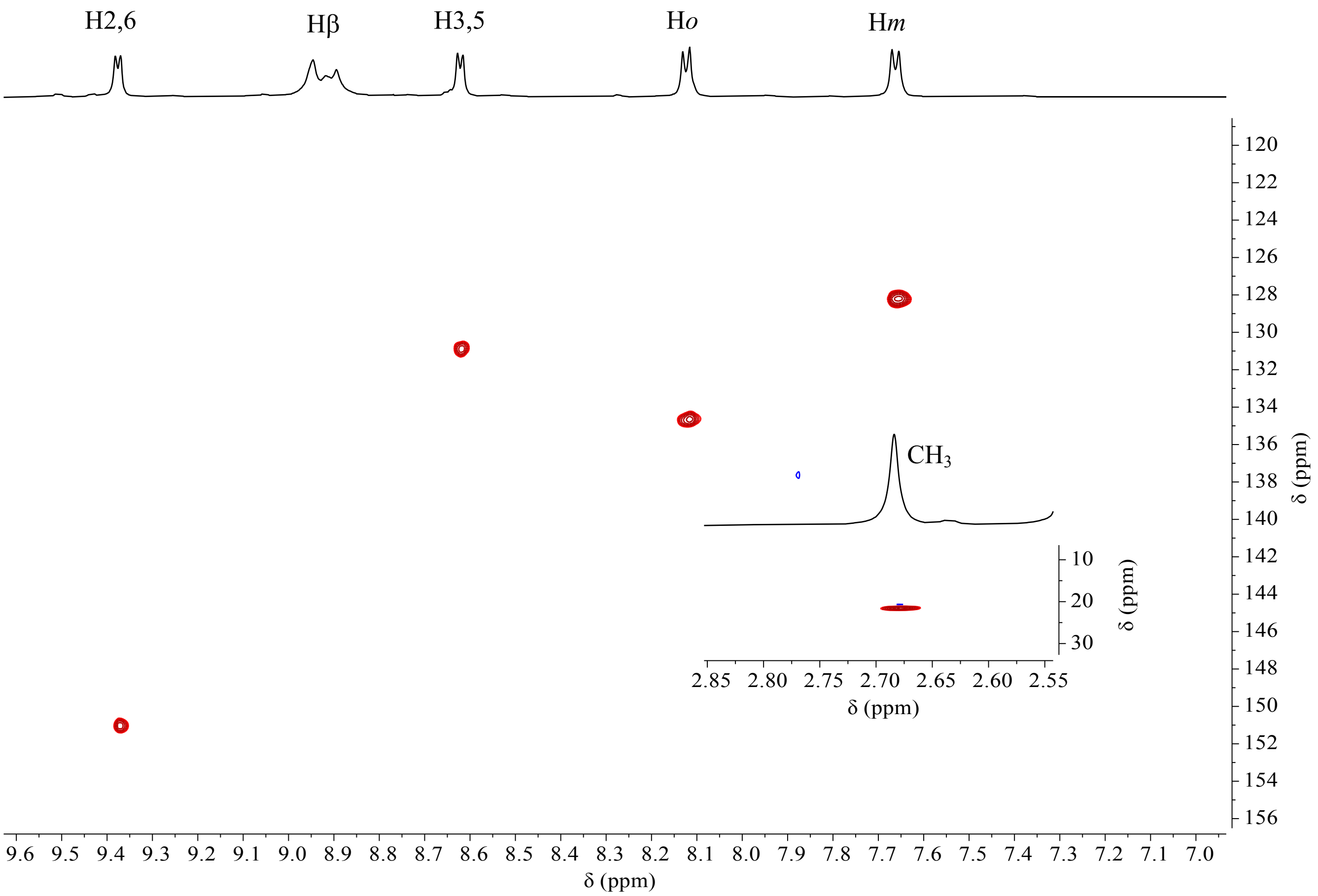

Figure S17. ^{1}H-^{13}C HSQC spectrum (aromatic region) in $CDCl_3$ + DMSO-d_6 of [{*t*,*c*,*c*-$RuCl_2(CO)_2$(dmso-O)}$_2$(4′*cis*DPyMP)] (**3Me**). See Fig. S16 for labelling scheme.

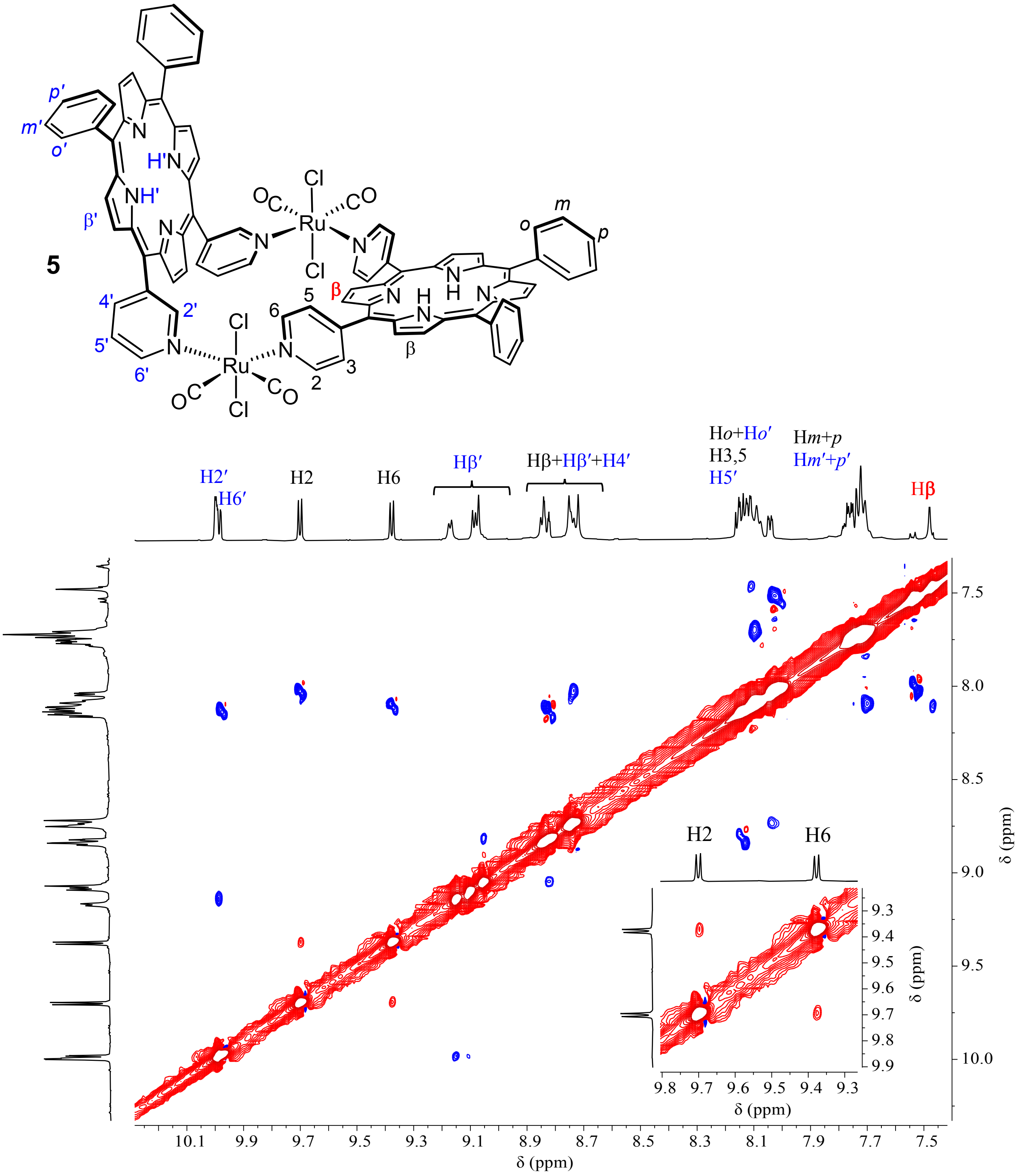

Figure S18. ^{1}H-^{1}H ROESY spectrum ($CDCl_3$) of [{*t,c,c*-$RuCl_2(CO)_2$}$_2$(4′*cis*DPyP)(3′*cis*DPyP)] (**5**) with labelling scheme and enlargement showing the exchange cross peaks (red) between the resonances of H2 and H6.

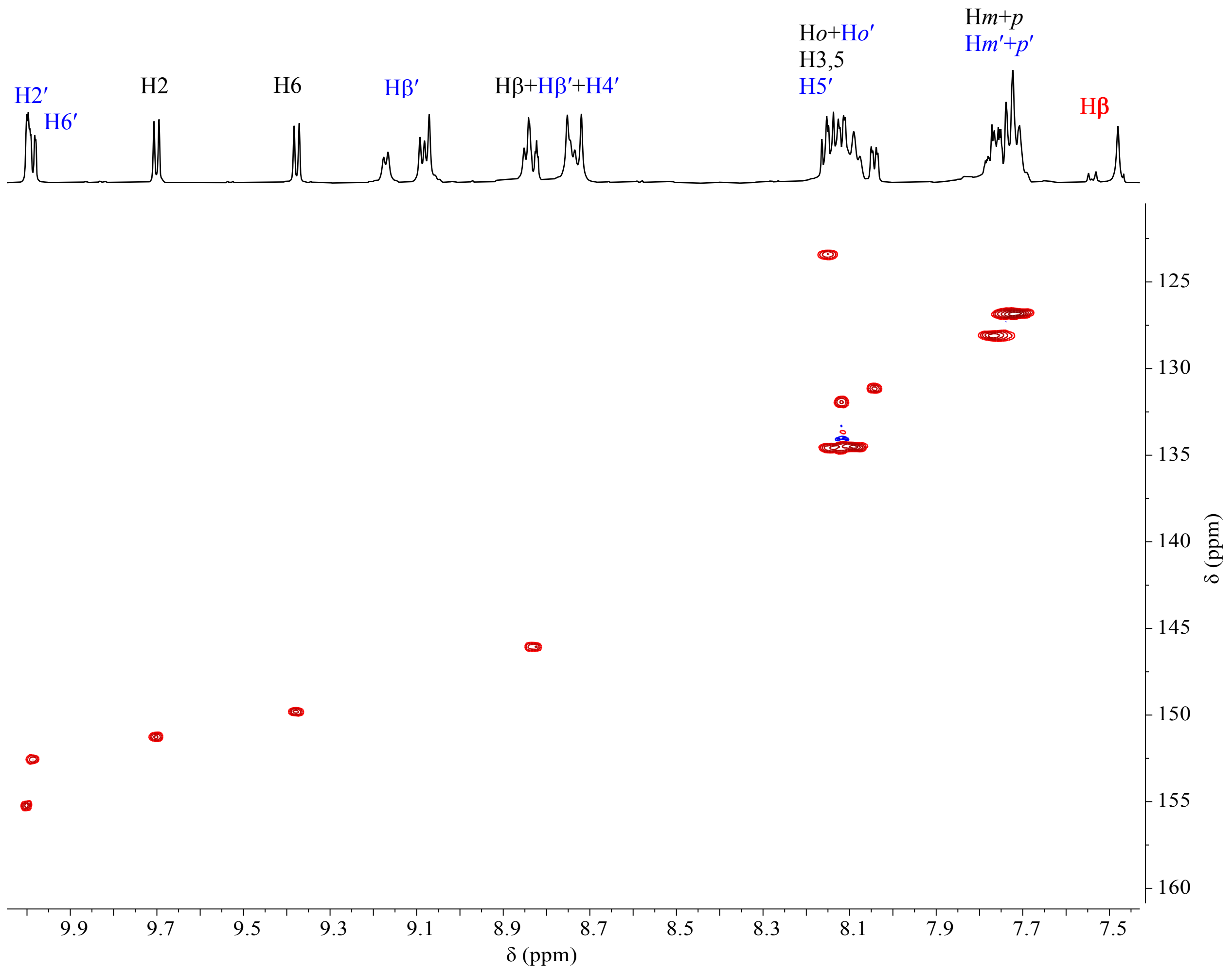

Figure S19. ^{1}H-^{13}C HSQC spectrum ($CDCl_3$) of [{*t,c,c*-$RuCl_2(CO)_2$}$_2$(4′*cis*DPyP)(3′*cis*DPyP)] (**5**), see Figure S18 for the labelling scheme.

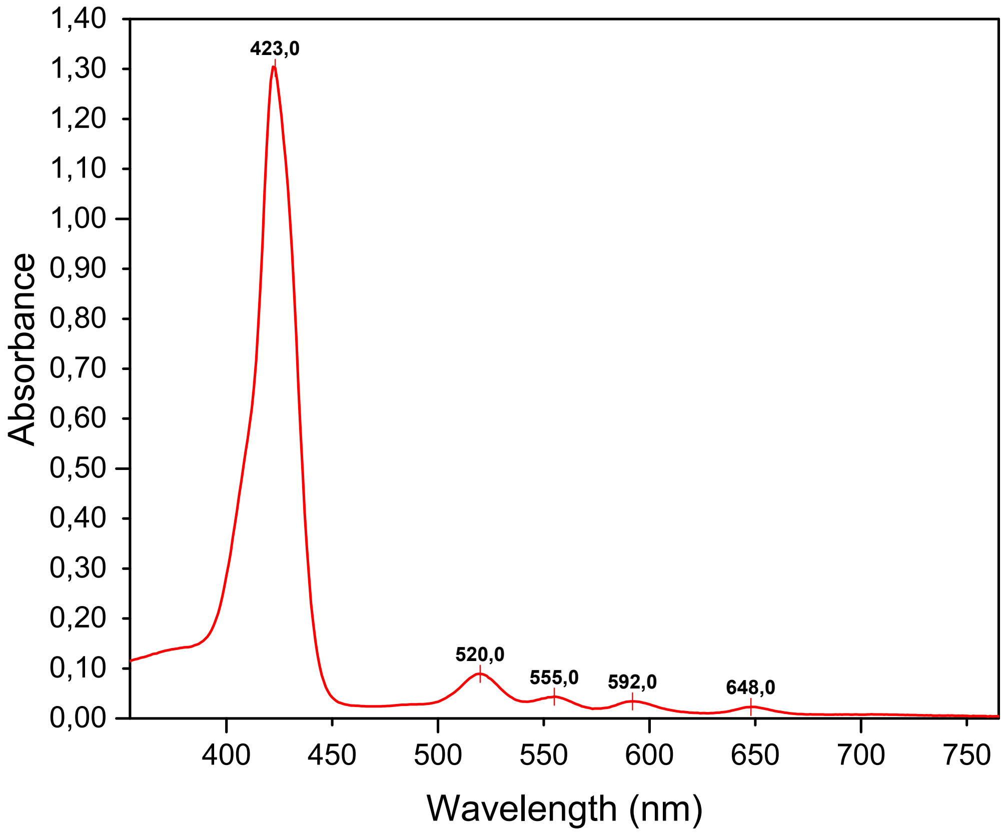

Figure S20. Normalized UV-vis absorption spectrum ($CHCl_3$) of [{*t,c,c*-$RuCl_2(CO)_2$}$_2$(4′*cis*DPyP)(3′*cis*DPyP)] (**5**).

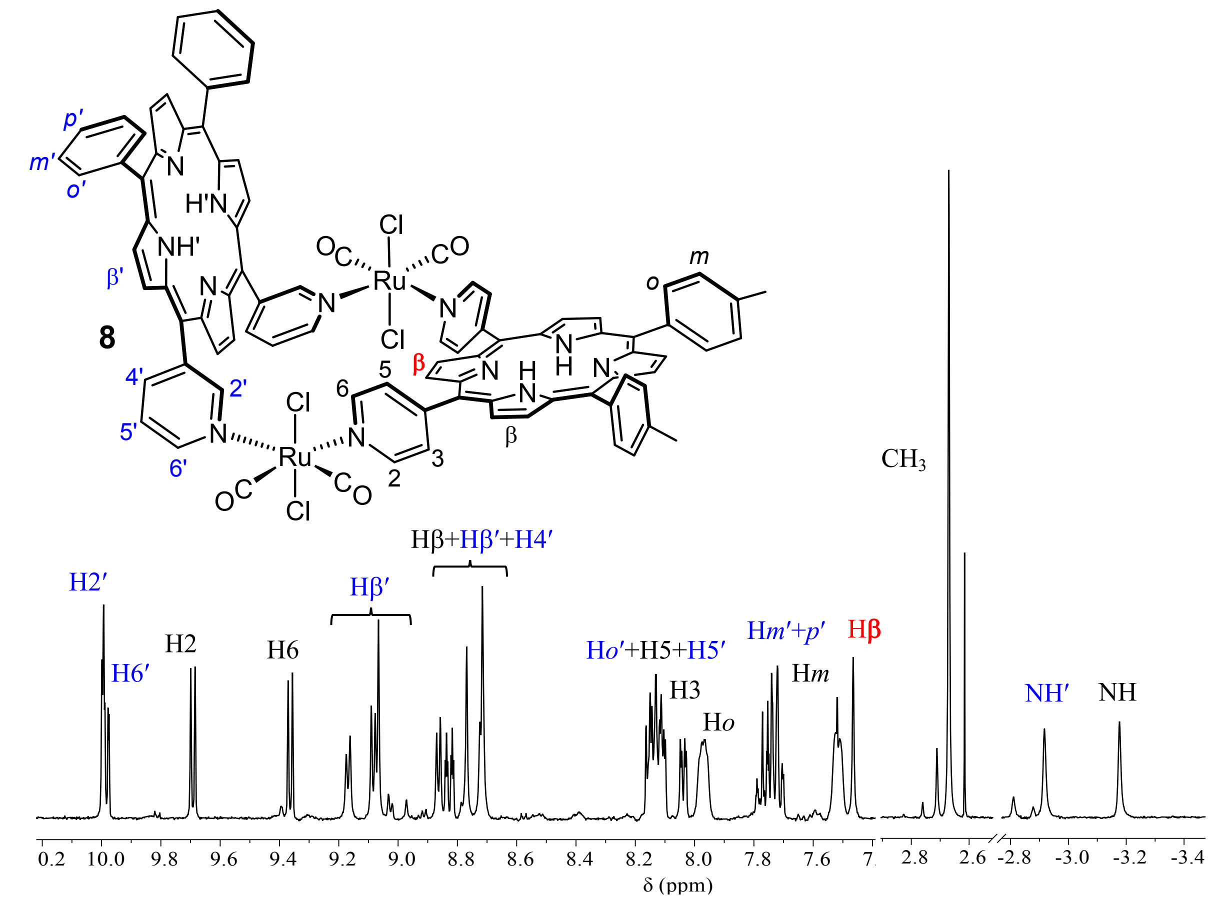

Figure S21. ^{1}H NMR spectrum ($CDCl_3$) of [{*t,c,c*-$RuCl_2(CO)_2$}$_2$(4′*cis*DPyMP)(3′*cis*DPyP)] (**8**) with labelling scheme. The resonances of 3′*cis*DPyP are labelled in blue.

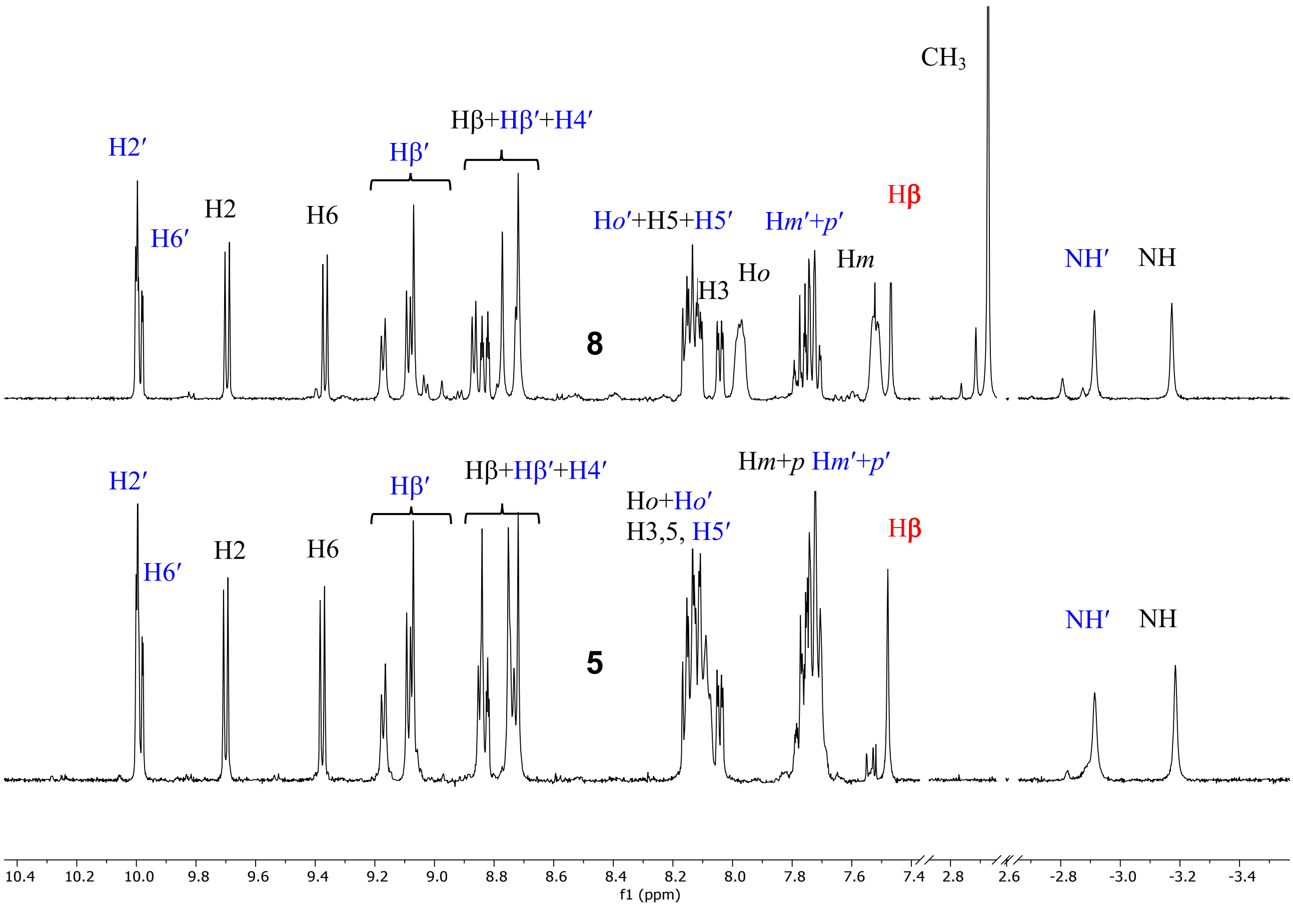

Figure S22. Comparison of the ^{1}H NMR spectra ($CDCl_3$) of [{*t,c,c*-$RuCl_2(CO)_2$}$_2$(4′*cis*DPyP)(3′*cis*DPyP)] (**5**, bottom) and [{*t,c,c*-$RuCl_2(CO)_2$}$_2$(4′*cis*DPyMP)(3′*cis*DPyP)] (**8**, top). See Figures S18 and S21 for the labelling schemes.

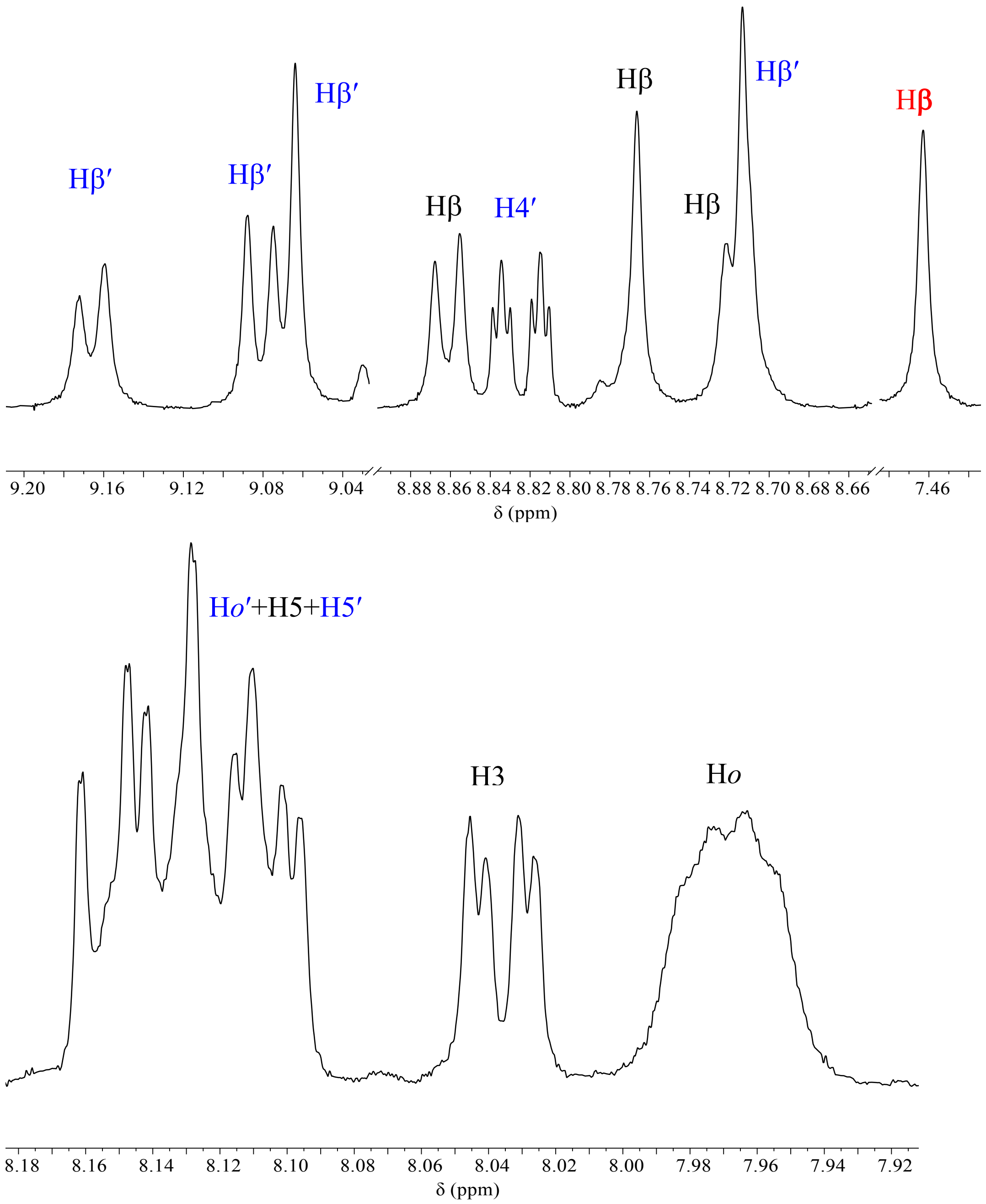

Figure S23. Enlargements of the ^{1}H NMR spectrum ($CDCl_3$) of [{*t,c,c*-$RuCl_2(CO)_2$}$_2$(4′*cis*DPyMP)(3′*cis*DPyP)] (**8**): top, region of the β-pyrrole resonances; bottom, region of the resonances of protons H5, H5', H*o*', H3, H*o*. See Figure S21 for the labelling scheme.

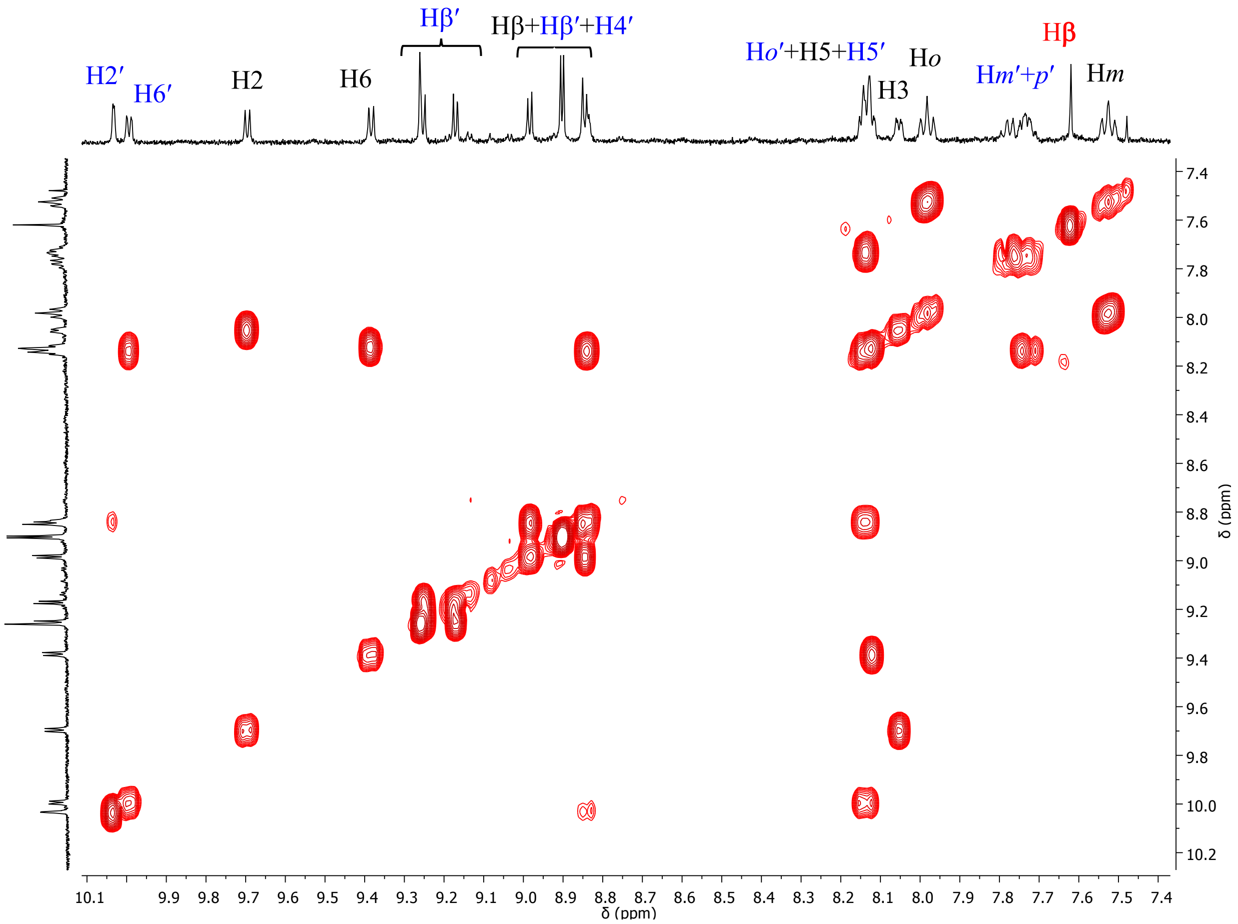

Figure S24. ^{1}H-^{1}H COSY spectrum (aromatic region, $CDCl_3$) of the heteroleptic 2+2 metallacycle [{*t*,*c*,*c*-$RuCl_2(CO)_2$}$_2$(Zn·4′*cis*DPyMP)(Zn·3′*cis*DPyP)] (**8Zn**). See Figure S21 for labelling scheme.

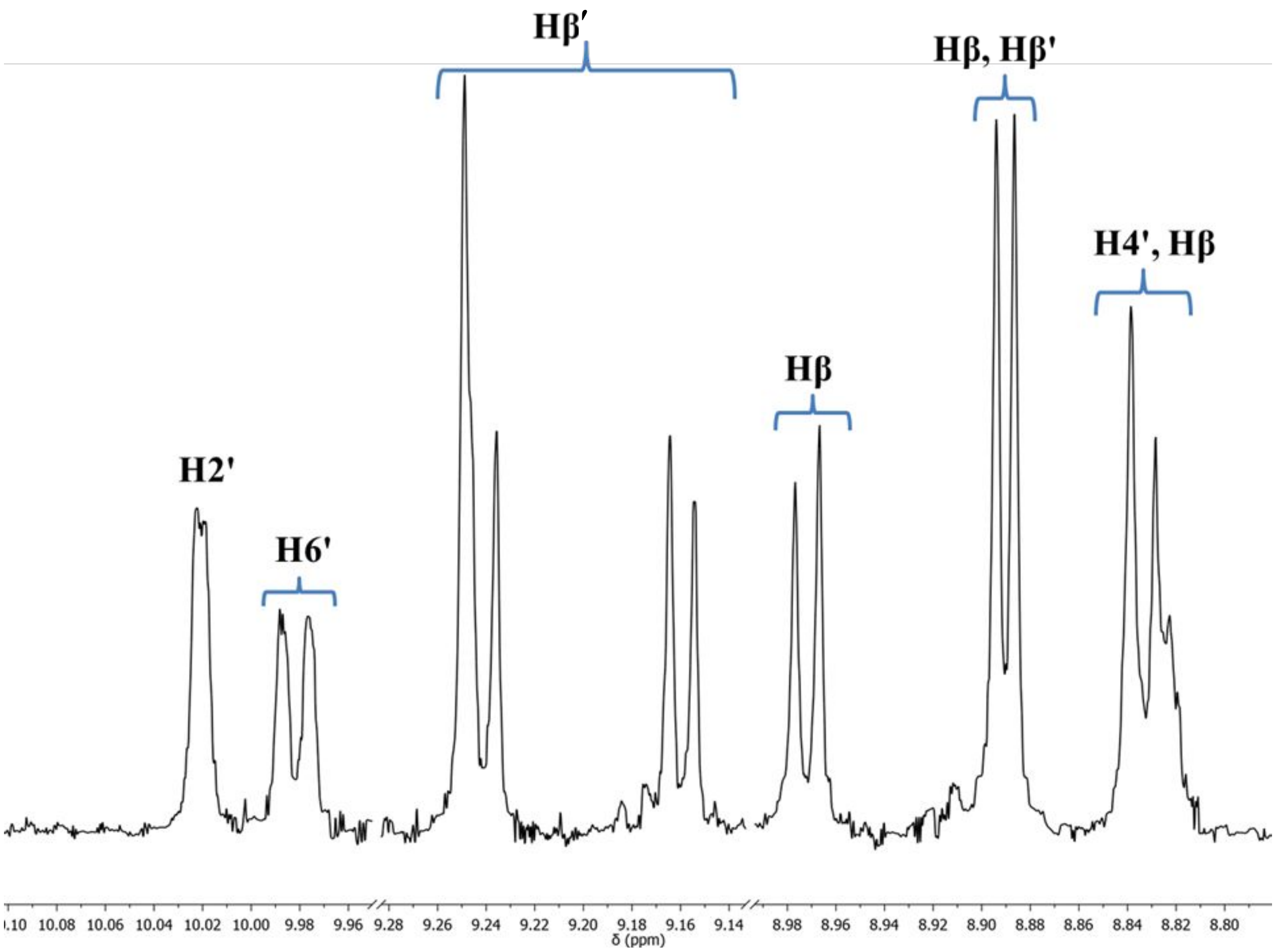

Figure S25. Enlargement of the ^{1}H NMR spectrum ($CDCl_3$) of the heteroleptic 2+2 metallacycle [{*t*,*c*,*c*-$RuCl_2(CO)_2$}$_2$(Zn·4′*cis*DPyMP)(Zn·3′*cis*DPyP)] (**8Zn**). See Figure S21 for labelling scheme.

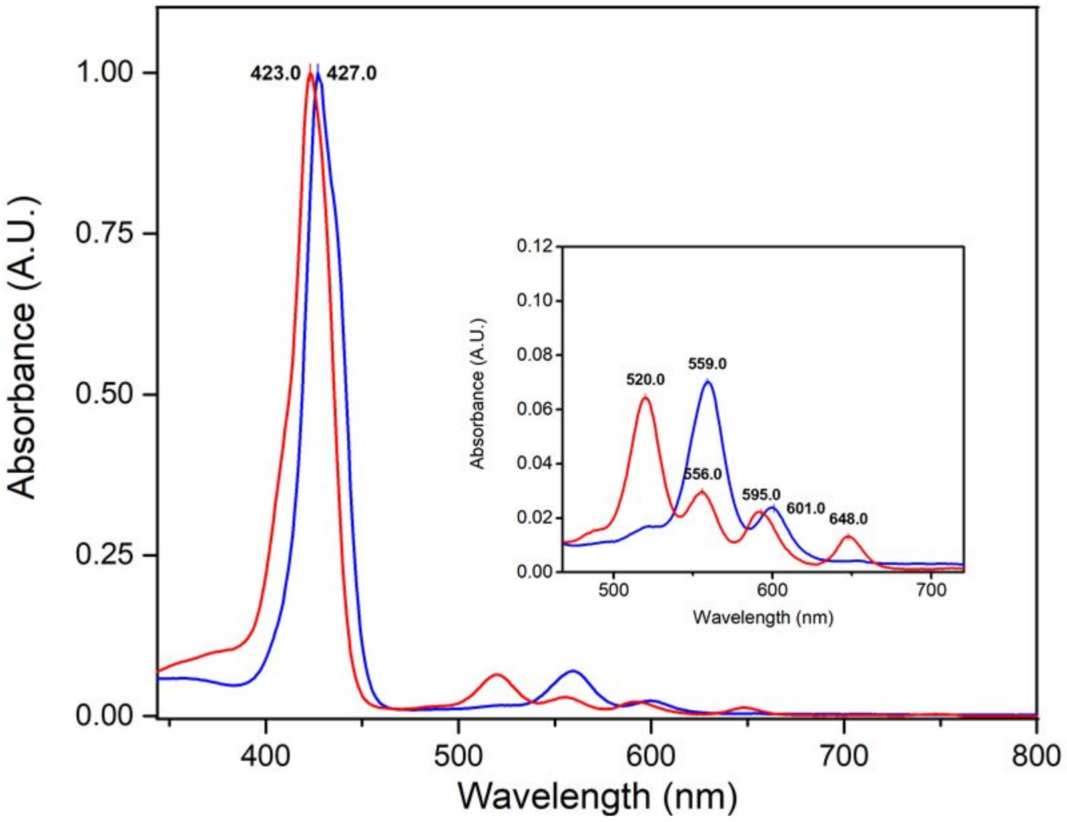

Figure S26. Normalized UV-vis absorption spectra ($CHCl_3$) of [{*t*,*c*,*c*-$RuCl_2(CO)_2$}$_2$(4′*cis*DPyMP)(3′*cis*DPyP)] (**8**, red) and [{*t*,*c*,*c*-$RuCl_2(CO)_2$}$_2$(Zn·4′*cis*DPyMP)(Zn·3′*cis*DPyP)] (**8Zn**, blue); in the insert the region of the Q-bands.

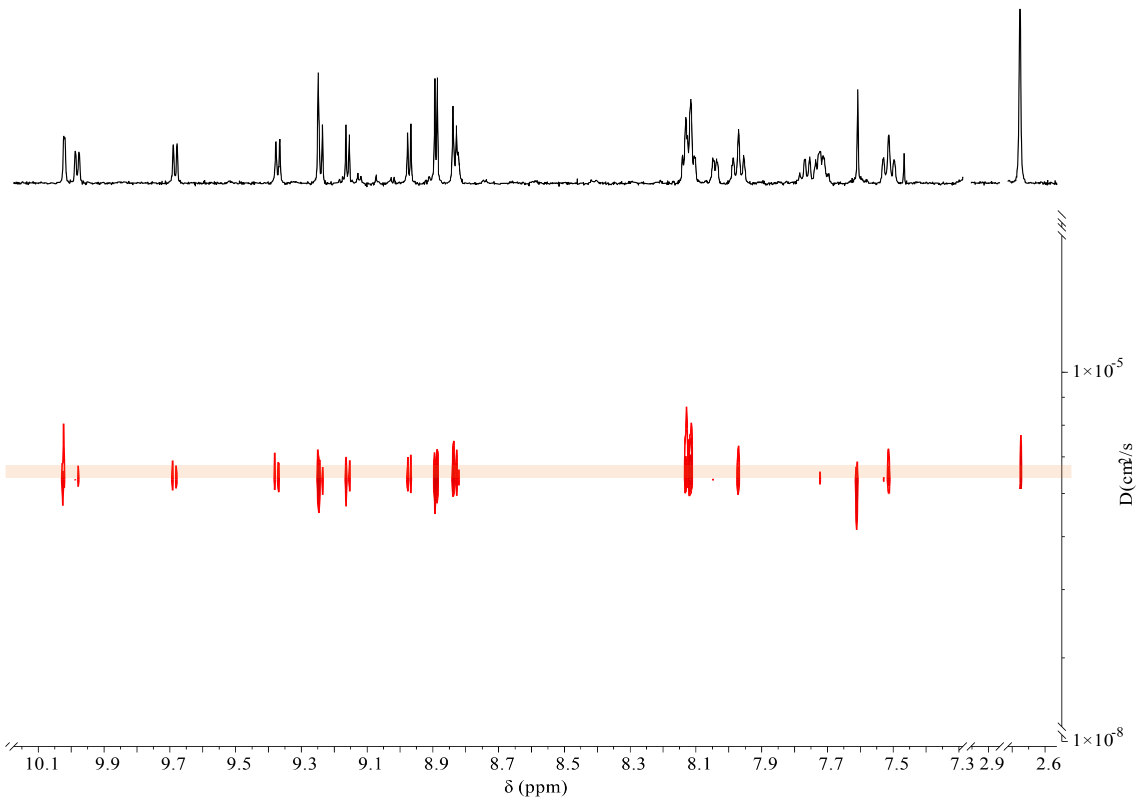

Figure S27. Bidimensional ^{1}H NMR DOSY spectrum of **8Zn**.

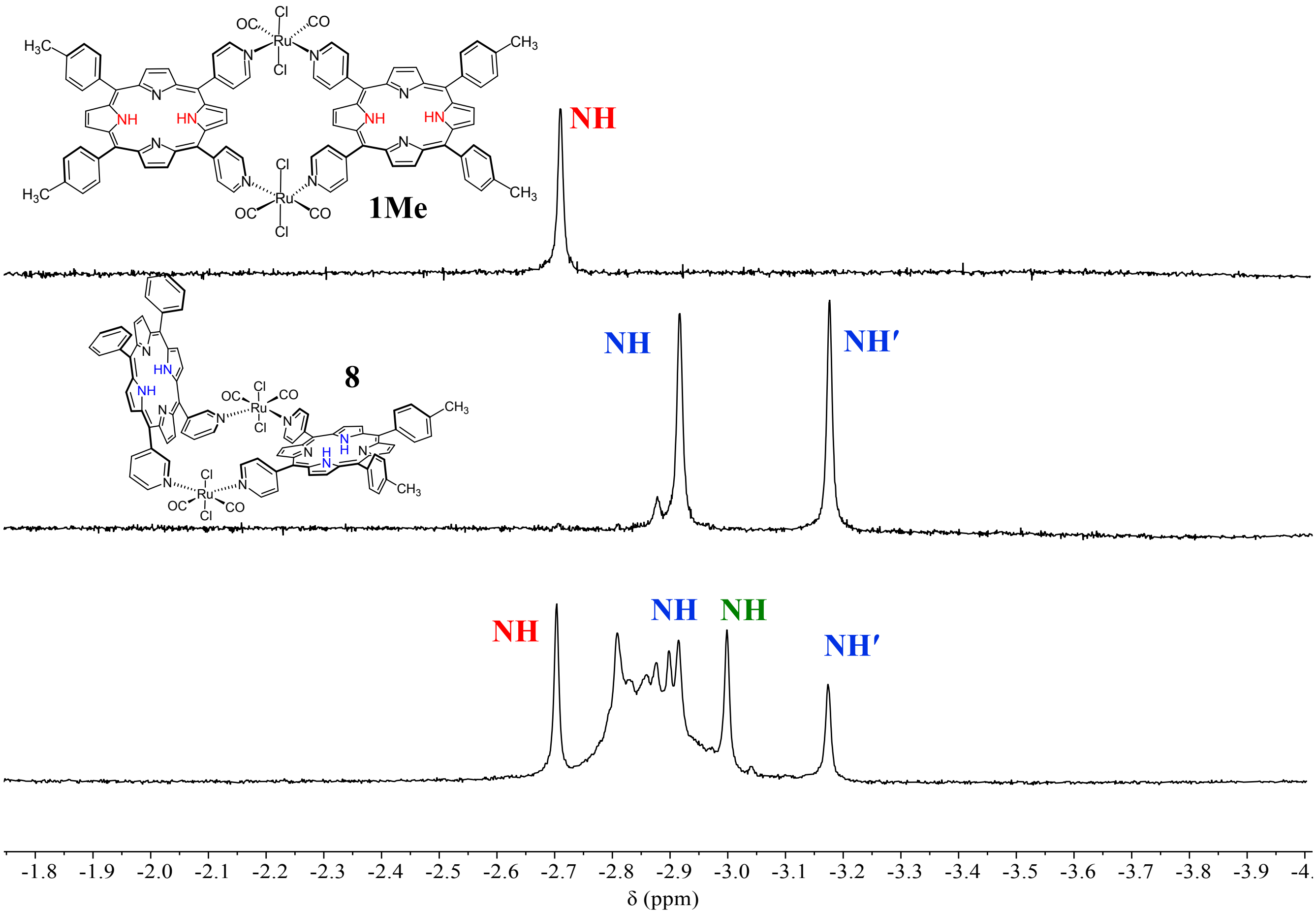

Figure S28. NH region of the ^{1}H NMR spectra of [*t*,*c*,*c*-$RuCl_2(CO)_2$(4′*cis*DPyMP)]$_2$ (**1Me**, top), **8** (middle), and the reaction crude (bottom), where the resonance of [*t*,*c*,*c*-$RuCl_2(CO)_2$(3′*cis*DPyP)]$_2$ (**2**) is labeled in green. The unidentified resonances in the bottom spectrum belong most likely to oligomeric species.

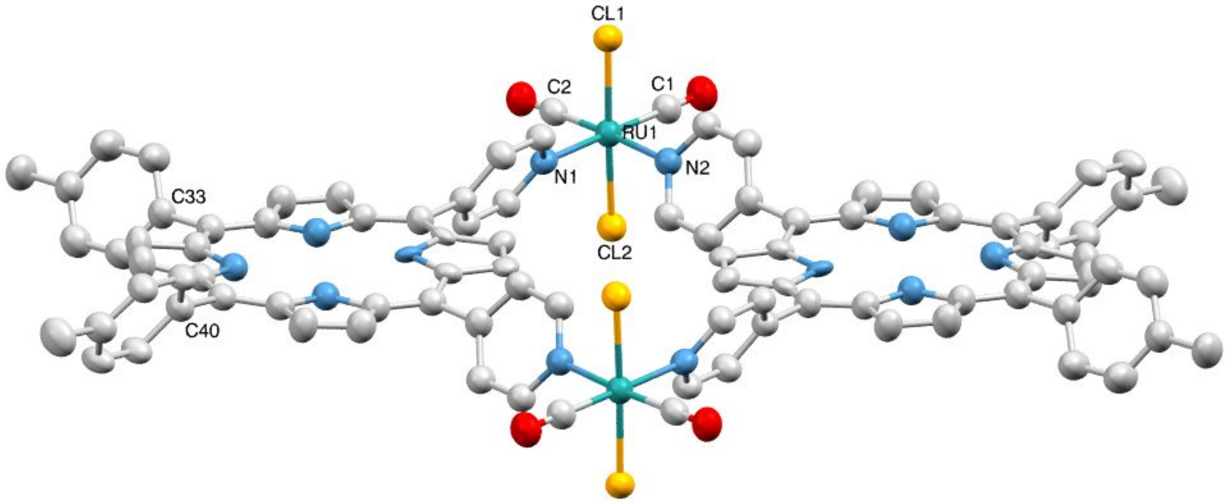

Figure S29. ORTEP representation (50% probability ellipsoids) of the molecule of compound [*t*,*c*,*c*-$RuCl_2(CO)_2$(4′*cis*DPyMP)$]_2 \cdot 2CH_2Cl_2 \cdot 7.3H_2O$ (**1Me**) in the crystal structure. The asymmetric unit is constituted by only one half of the whole metallacycle. H atoms, disordered water molecules and a CH_2Cl_2 solvent have been omitted for clarity. See Table S2 for dihedral angles

Table S1. Crystallographic data and refinement details for compound [*t,c,c*-$RuCl_2(CO)_2$(4′*cis*DPyMP)]$_2$ (**1Me**).

	**1Me**
Formula	$Ru_2Cl_4C_{92}O_4N_{12}H_{64}\cdot 2CH_2Cl_2\cdot 7.3H_2O$
Formula weight (Da)	2046.90
Temperature (K)	100(2)
Wavelength (Å)	0.700
Crystal System	monoclinic
Space Group	P 21/c
a (Å)	17.756(4)
b (Å)	8.737(9)
c (Å)	30.803(5)
α (°)	90
β (°)	94.90(1)
γ (°)	90
V ($Å^3$)	4761(5)
Z	4
ρ (g cm^{-3})	1.428
F(000)	2090
μ (mm^{-1})	0.574
θ_{min}, θ_{max} (°)	1.802,28.650
Resolution (Å)	0.73
Total refl. collctd.	79919
Independent refl.	12731
Obs. Refl. $F_o>4\sigma_{Fo}$	9106
I/σ (all data)	19.84
I/σ (max resltn)	3.11
R_{merge} (all data)	4.8%
R_{merge} (max resltn)	51.4%
Completeness (all data)	0.995
Multiplicity (all data)	6.1
Multiplicity (max resltn)	6.0
Data/restraint/parameters	12731/40/624
$R_{I>2\sigma I}$,$wR_{2,I>2\sigma I}$	0.0850, 0.2162
R (all data), wR_2 (all data)	0.1201, 0.2378
GooF	1.033

Table S2. Selected coordination distances (Å) and angles (°) for compound [*t,c,c*-$RuCl_2(CO)_2$(4′*cis*DPyMP)]$_2$ (**1Me**).

Selected distances (Å)

Ru1	C1	1.873(5)
Ru1	C2	1.884(5)
Ru1	Cl1	2.402(3)
Ru1	Cl2	2.398(3)
Ru1	N1	2.150(4)
Ru1	N2	2.161(4)

Selected angles (°)

C1	Ru1	C2	89.3(2)
C1	Ru1	Cl1	88.3(2)
C1	Ru1	Cl2	89.6(2)
C1	Ru1	N1	177.6(2)
C1	Ru1	N2	93.33(2)
C2	Ru1	Cl1	91.4(2)
C2	Ru1	Cl2	88.6(2)
C2	Ru1	N1	93.0(2)
C2	Ru1	N2	176.9(2)
Cl2	Ru1	Cl1	177.82(4)
N1	Ru1	Cl1	91.2(1)
N1	Ru1	Cl2	91.0(1)
N1	Ru1	N2	84.4(1)
N2	Ru1	Cl1	90.3(1)
N2	Ru1	Cl2	89.8(1)

Selected dihedral angles (°)*

[Por]	[Ru1]	3.1(1)
[Por]	[C33]	58.0(2)
[Por]	[C40]	57.9(2)
[Por]	[N1]	57.2(2)
[Por]	[N2]	60.3(2)

*YY in [YY] is the atom label which allows to identify the planar moiety in Figure S29; [Por] stands for the least squares plane through the N atoms of the porphyrin.
